# A role for triglyceride lipase *brummer* in the regulation of sex differences in *Drosophila* fat storage and breakdown

**DOI:** 10.1371/journal.pbio.3000595

**Published:** 2020-01-21

**Authors:** Lianna W. Wat, Charlotte Chao, Rachael Bartlett, Justin L. Buchanan, Jason W. Millington, Hui Ju Chih, Zahid S. Chowdhury, Puja Biswas, Vivian Huang, Leah J. Shin, Lin Chuan Wang, Marie-Pierre L. Gauthier, Maria C. Barone, Kristi L. Montooth, Michael A. Welte, Elizabeth J. Rideout

**Affiliations:** 1 Department of Cellular and Physiological Sciences, Life Sciences Institute, The University of British Columbia, Vancouver, British Columbia, Canada; 2 School of Biological Sciences, University of Nebraska-Lincoln, Lincoln, Nebraska, United States of America; 3 Department of Biology, University of Rochester, Rochester, New York, United States of America; ICM, FRANCE

## Abstract

Triglycerides are the major form of stored fat in all animals. One important determinant of whole-body fat storage is whether an animal is male or female. Here, we use *Drosophila*, an established model for studies on triglyceride metabolism, to gain insight into the genes and physiological mechanisms that contribute to sex differences in fat storage. Our analysis of triglyceride storage and breakdown in both sexes identified a role for triglyceride lipase *brummer* (*bmm*) in the regulation of sex differences in triglyceride homeostasis. Normally, male flies have higher levels of *bmm* mRNA both under normal culture conditions and in response to starvation, a lipolytic stimulus. We find that loss of *bmm* largely eliminates the sex difference in triglyceride storage and abolishes the sex difference in triglyceride breakdown via strongly male-biased effects. Although we show that *bmm* function in the fat body affects whole-body triglyceride levels in both sexes, in males, we identify an additional role for *bmm* function in the somatic cells of the gonad and in neurons in the regulation of whole-body triglyceride homeostasis. Furthermore, we demonstrate that lipid droplets are normally present in both the somatic cells of the male gonad and in neurons, revealing a previously unrecognized role for *bmm* function, and possibly lipid droplets, in these cell types in the regulation of whole-body triglyceride homeostasis. Taken together, our data reveal a role for *bmm* function in the somatic cells of the gonad and in neurons in the regulation of male–female differences in fat storage and breakdown and identify *bmm* as a link between the regulation of triglyceride homeostasis and biological sex.

## Abstract 2

An investigation of the genetic and physiological mechanisms underlying sex differences in fat storage and breakdown in the fruit fly Drosophila identifies previously unrecognized sex- and cell type-specific roles for the conserved triglyceride lipase brummer.

## Introduction

Triglycerides are the main form of stored fat in animals and are stored in lipid droplets within specialized fat storage organs, such as the adipose tissue in mammals or the fat body in insects [1–5]. One important but often overlooked factor that affects fat storage is whether the animal is male or female [6,7]. In mammals, females store approximately 10% more body fat than males [7–9], whereas in some insect species, females store up to four times more fat than males [10]. An extensive literature has revealed the important role of sex hormones and sex chromosomes in establishing this male–female difference in fat storage [7,11,12]. For example, the female sex steroid estrogen and the presence of two X chromosomes both contribute to the increased fat storage in female mice [7,11]. Although these sex-determining factors in mice, and in other animals, have been shown to promote extensive sex-biased expression of many genes, including genes involved in fat storage [13–16], the downstream metabolic genes that contribute to the sex difference in fat storage are only beginning to be uncovered.

Over the past 15 years, *Drosophila* has emerged as a powerful model to investigate the in vivo function of genes that are involved in the regulation of triglyceride synthesis, storage, and breakdown [4,17–19]. The main pathway of triglyceride synthesis in flies begins with the acylation of glycerol-3-phosphate to produce lysophosphatidic acid, a reaction that is catalyzed by glycerol-3-phosphate acyltransferases (GPATs) [4]. Flies have several genes that encode putative GPATs: *minotaur* (*mino*; FBgn0027579), *Gpat4* (FBgn0034971), and the testis-specific *CG15450* (FBgn0031132). Although previous studies confirmed a role for *mino* in triglyceride synthesis by demonstrating that *mino* overexpression leads to large lipid droplets in the larval salivary gland [20], the functional roles of *Gpat4* and *CG15450* in triglyceride metabolism remain largely unconfirmed. The second step in triglyceride synthesis is catalyzed by 1-acylglycerol-3-phosphate *O*-acyltransferases (AGPATs), which acylate lysophosphatidic acid to produce phosphatidic acid [4]. *Drosophila* has four genes that encode potential AGPAT proteins: *Agpat1* (FBgn0030421), *Agpat2* (FBgn0026718), *Agpat3* (FBgn0036623), and *Agpat4* (FBgn0036622). At present, these predicted AGPAT enzymes are largely uncharacterized [4]; however, *Agpat2*, *Agpat3*, and *Agpat4* are all expressed in the fat body, a critical lipid-storing organ [21]. Once phosphatidic acid is produced, it is dephosphorylated in the third step of triglyceride synthesis by the phosphatase Lipin into diglyceride. The *Drosophila* genome contains a single *Lipin* gene (*Lpin*; FBgn0263593) that is expressed in fat-storing organs in flies [21], and *Lpin* loss alters lipid droplet size and impairs whole-body triglyceride storage [22].

The final step in triglyceride synthesis is the acylation of diglyceride into triglyceride by diacylglycerol *O*-acyltransferases (DGATs) [4]. In *Drosophila*, the only characterized DGAT family member is called *midway* (*mdy*; FBgn0004797) [4]. Studies have shown that loss of *mdy* significantly impairs triglyceride synthesis and reduces whole-body triglyceride levels [23,24], whereas *mdy* overexpression increases the number of small lipid droplets in the larval salivary gland [20]. In addition to *mdy*, the *Drosophila* genome also contains genes that encode proteins from a related family of enzymes that show DGAT activity, which is called the DAGAT family [4]. In flies, there are three members of this DAGAT family: *CG1941* (FBgn0033214), *Dgat2* (FBgn0033215), and *CG1946* (FBgn0033216), all of which are functionally and biochemically uncharacterized. Once triglyceride synthesis is complete, triglycerides accumulate between the two leaflets of the phospholipid bilayer in the endoplasmic reticulum to form a lipid lens [5]. This lipid lens eventually buds off from the endoplasmic reticulum to form an organelle called a lipid droplet. The neutral lipid core of the lipid droplet is separated from the cytoplasmic contents of the cell by a phospholipid monolayer. Studies show that many proteins associated with this monolayer play key roles in regulating lipid droplet size, as well as cellular and organismal levels of triglyceride storage [5,25–28]. For example, members of the perilipin (PLIN) family of proteins in *Drosophila* associate with lipid droplets and influence lipid droplet size in vivo [29–31]. The *Drosophila* genome encodes two PLIN family members: *lipid storage droplet-1* (*lsd-1*/*PLIN1*; FBgn0039114) and *lipid storage droplet-2* (*lsd-2*/*PLIN2*; FBgn0030608). Importantly, altered expression of either *lsd-1*/*PLIN1* or *lsd-2*/*PLIN2* impacts lipid droplet size and affects whole-body triglyceride storage [29–31].

Triglyceride breakdown in *Drosophila* occurs in a fixed series of enzymatic reactions [4]. The first step in *Drosophila* triglyceride breakdown is triglyceride hydrolysis, which produces a free fatty acid and diglyceride [4]. In the *Drosophila* genome, the best-characterized triglyceride lipase is *brummer* (*bmm*; FBgn0036449), a member of the Patatin-like domain-containing family that catalyzes triglyceride hydrolysis in vitro and in vivo [32]. Importantly, loss of *bmm* function increases lipid droplet size and augments whole-body triglyceride storage, whereas *bmm* overexpression decreases lipid droplet size and depletes triglyceride levels [32], demonstrating a key role for *bmm* in regulating triglyceride homeostasis in vivo. Other than *bmm*, the *Drosophila* genome contains more than 50 predicted lipases, only a few of which have been characterized [33]. For example, larvae with loss of *hormone-sensitive lipase* (*hsl*; FBgn0034491) have larger lipid droplets and higher triglyceride levels than controls [30]. Additional genes with potential effects on triglyceride mobilization include another Patatin-like domain-containing family member called *doppelganger von brummer* (*dob*; FBgn0030607) and *CG5966* (FBgn0029831). In addition to the essential role that lipases such as *bmm* have in promoting triglyceride breakdown, lipid droplet–associated proteins also make important contributions to lipolysis. For example, *lsd-1/PLIN1* and *lsd-2/PLIN2* influence triglyceride breakdown by regulating the access of key lipases such as *bmm* to their triglyceride substrate [4]. Together, these studies highlight the important contribution of *Drosophila* to our current knowledge of the molecular mechanisms underlying the regulation of whole-body triglyceride levels in vivo.

In addition to revealing the mechanisms underlying the regulation of cellular and organismal triglyceride levels, studies in *Drosophila* have significantly advanced our knowledge of how triglyceride homeostasis impacts life span, starvation resistance, and fertility [4]. For example, studies have shown that flies with reduced function of *mdy*, the enzyme that catalyzes the final step of triglyceride synthesis, have impaired egg chamber development and female sterility [23,29,34,35]. Another gene with well-studied effects on cellular and organismal phenotypes is triglyceride lipase *bmm*: male flies with reduced *bmm* function have increased starvation resistance, exaggerated sleep rebound following sleep deprivation [36], and a modest reduction in life span [32,34]. Finally, several phenotypes have been associated with *lsd-1*/*PLIN1* and *lsd-2*/*PLIN2*, such as starvation resistance [29–31] and sleep rebound after sleep deprivation [36]. Thus, the correct regulation of triglyceride storage and breakdown impacts many aspects of *Drosophila* development, physiology, and life history.

In the present study, we aimed to improve our knowledge of the metabolic genes and physiological mechanisms that contribute to male–female differences in *Drosophila* triglyceride homeostasis. Although whole-body triglyceride storage is known to differ between mated female and male flies [37–39], most studies on triglyceride synthesis and breakdown use male flies or mixed-sex groups of larvae to determine how individual genes affect these processes. As a result, the downstream genes and mechanisms that contribute to the sex difference in triglyceride storage, and possibly other aspects of triglyceride homeostasis, remain incompletely understood. Our detailed examination of triglyceride storage and breakdown in adult male and female flies revealed significant sexual dimorphism in both aspects of triglyceride homeostasis and identified a role for one gene, triglyceride lipase *bmm*, in regulating sex differences in triglyceride storage and breakdown. Normally, females have more triglyceride storage than males and slower triglyceride breakdown in response to a lipolytic stimulus. Loss of *bmm* largely abolishes the sex difference in triglyceride storage and eliminates the sex difference in triglyceride breakdown via strongly male-biased effects. Importantly, we discovered that *bmm* function in the somatic cells of the gonad and in neurons, two cell types previously unknown to require *bmm* function, plays a role in regulating sex differences in triglyceride homeostasis. Because we show that lipid droplets, the intracellular fat storage organelle, are present in both cell types under normal physiological conditions, our findings illuminate an unexpected role for *bmm* function, and possibly lipid droplets, in these two cell types in the regulation of sex differences in triglyceride storage and breakdown.

## Results

### Sexual dimorphism in triglyceride storage and breakdown

**Fig 1:**
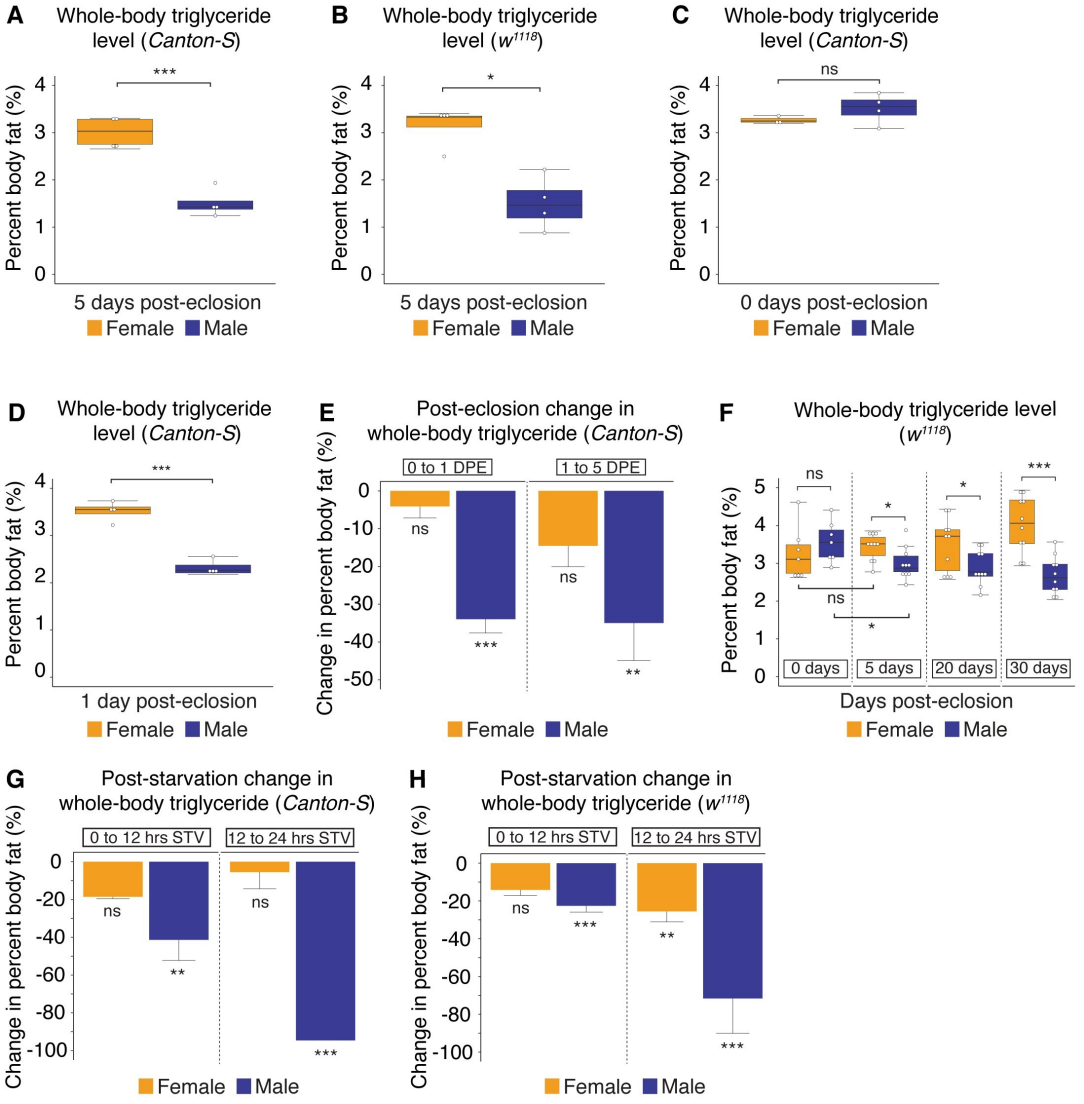
Sexual dimorphism in *Drosophila* triglyceride storage and breakdown. (A) Whole-body triglyceride storage in 5-day-old *Canton-S* virgin females was significantly higher than in age-matched *Canton-S* virgin male flies (*p* = 5.4 × 10^−4^; Student *t* test). (B) Whole-body triglyceride storage in 5-day-old *w*^*1118*^ virgin female flies was significantly higher than in age-matched *w*^*1118*^ virgin male flies (*p* = 2.3 × 10^−2^; Student *t* test). (C) No significant difference in whole-body triglyceride storage was found between newly eclosed virgin *Canton-S* females and age-matched virgin males (*p* = 0.73; Student *t* test). (D) Whole-body triglyceride storage in 1-day-old *Canton-S* virgin females was significantly higher than in age-matched virgin males (*p* = 1.2 × 10^−4^; Student *t* test). (E) In females, whole-body triglyceride levels were not significantly different between newly eclosed flies and flies collected at 1 DPE or between flies collected at 1 DPE and 5 DPE (*p* = 0.91 and 0.38, respectively; one-way ANOVA followed by Tukey HSD test). In males, whole-body triglyceride storage was significantly lower at 1 DPE than in newly eclosed flies, with a further reduction in triglyceride storage between 1 DPE and 5 DPE (*p* = 4.2 × 10^−4^ and 5.7 × 10^−3^, respectively; one-way ANOVA followed by Tukey HSD test). (F) Whole-body triglyceride storage in *w*^*1118*^ virgin females was not significantly higher than males at eclosion, but it was significantly higher by 5 DPE, a sex difference that was maintained in 20- and 30-day-old males and females (*p* = 0.38, 0.024, 0.029, 1.5 × 10^−4^, respectively; Student *t* test at each time point). (G) In 5-day-old *Canton-S* virgin females, there was no significant difference in whole-body triglyceride levels between 0 and 12 hours STV or between 12 and 24 hours STV (*p* = 0.097, 0.92, respectively; one-way ANOVA followed by Tukey HSD test). In males, there was a significant decrease in whole-body triglyceride storage between 0 and 12 hours STV and a further decrease in triglyceride levels between 12 and 24 hours STV (*p* = 2.2 × 10^−3^, 1.7 × 10^−4^, respectively; one-way ANOVA followed by Tukey HSD test). (H) In 5-day-old *w*^*1118*^ virgin females, there was no significant difference in whole-body triglyceride levels between 0 and 12 hours STV and a modest difference between 12 and 24 hours STV (*p* = 0.11, 2.2 × 10^−3^, respectively; one-way ANOVA followed by Tukey HSD test). In 5-day-old *w*^*1118*^ virgin males, we observed a significant decrease in triglyceride levels between 0 and 12 hours STV and a further decrease between 12 and 24 hours STV (*p* = 3.0 × 10^−5^, 0.0, respectively; one-way ANOVA followed by Tukey HSD test). Asterisks indicate a significant difference between two sexes, two genotypes, or two time points (*p < 0.05, **p < 0.01, ***p < 0.001). Error bars on graphs depicting percent body fat represent SEM; error bars on graphs depicting the change in percent body fat represent COE. See S1 Table for all multiple comparisons and *p*-values; quantitative measurements underlying all graphs are available in S1 Data. COE, coefficient of error; DPE, days post-eclosion; HSD, honest significant difference; ns, no significant difference between two sexes, two genotypes, or time points; STV, post-starvation; *w*, *white*.

Adult mated females have increased levels of triglyceride storage compared with males [37–39]. To determine whether this increased triglyceride storage in females reflects a mating-induced change to female physiology or a sexual dimorphism in triglyceride storage, we measured whole-body triglyceride storage in *Canton-S* (*CS*) virgin females and males. In 5-day-old adults, virgin females have increased levels of triglyceride storage compared with virgin males (Fig 1A; see S1 Table for all *p*-values). This difference was also present when we compared whole-body triglyceride storage in *white* (*w*)^*1118*^ virgin males and females (Fig 1B). Because we observed no significant differences in triglyceride storage between 5-day-old *CS* and *w*^*1118*^ virgin females or between 5-day-old *CS* and *w*^*1118*^ virgin males (S2 Table) our findings show that the sexual dimorphism in triglyceride storage persists in multiple genetic backgrounds. Although one obvious explanation for the sexual dimorphism in triglyceride storage is triglyceride contained within the male and female gonads, we confirm previous findings that ovary triglyceride levels represent only a fraction of the whole-body triglyceride level in females (S1A Fig) [37] and show that triglyceride levels in the testis do not significantly contribute to the whole-body triglyceride level in males (S1B Fig). Furthermore, we found that the sexual dimorphism in triglyceride storage was preserved between *w*^*1118*^ virgin male and female carcasses devoid of gonads (S1C Fig). Thus, the sex difference in triglyceride storage cannot be solely attributed to the triglyceride stored in the male and female gonads.

To determine when this sexual dimorphism in triglyceride storage was established, we examined triglyceride levels in adult virgin male and female flies at several times post-eclosion. In newly eclosed flies, where larval fat is still present [40], there was no significant difference in triglyceride levels between *CS* virgin females and males (Fig 1C). By 1 day post-eclosion (DPE), however, triglyceride levels in *CS* virgin females were significantly higher than in age-matched males (Fig 1D), and by 5 DPE, triglyceride levels in *CS* virgin females were approximately 2.2 times higher than in *CS* virgin males (Fig 1A). When we examined triglyceride storage during early adult life within *CS* flies of each sex, we found that whole-body triglyceride storage at 1 DPE in *CS* males was significantly lower than in newly eclosed *CS* males and significantly lower in males at 5 DPE compared with males at 1 DPE (Fig 1E). In females, we found no significant changes to whole-body triglyceride levels during this 5-day period (Fig 1E). Thus, the sexual dimorphism in *CS* triglyceride storage was established over the first 5 days of adult life by a progressive reduction in whole-body triglyceride storage in males, a finding we also confirm in *w*^*1118*^ (Fig 1F and S2 Table). Given that previous studies showed that, in females, approximately 50% of the larval fat cells disappear within 9 hours post-eclosion [40], one possible explanation for the reduction in triglyceride levels in males post-eclosion is a male–female difference in the persistence of larval fat cells. We therefore counted the number of larval fat cells in *CS* and *w*^*1118*^ males and females at 12-hour intervals post-eclosion. We found that larval fat cells were largely eliminated in both sexes between 0 and 24 hours post-eclosion (S1D and S1E Fig); however, there was no obvious sex difference in the timing of larval fat cell loss that would explain the male–female difference in triglyceride storage that is established over the first 5 days of adult life. Once this difference is established, we show that the sexual dimorphism in triglyceride storage persists until at least 30 DPE (Fig 1F).

In addition to sexual dimorphism in triglyceride storage, male–female differences in fat breakdown have also been reported in mammals [7,41]. We therefore examined changes to whole-body triglyceride levels in response to starvation, a lipolytic stimulus, in virgin males and females. In *CS* 5-day-old virgin males, we observed a 41% decrease in whole-body triglyceride levels between 0 (fed flies) and 12 hours post-starvation and a further reduction in triglyceride levels between 12 and 24 hours post-starvation (Fig 1G). As a result of this substantial reduction in whole-body triglyceride levels post-starvation, whole-body triglyceride stores were largely depleted in virgin males by 24 hours post-starvation (S1F Fig), a finding that is in line with previous studies [29,30,32]. In contrast, we found no significant change in whole-body triglyceride levels in 5-day-old virgin *CS* females between either 0 and 12 hours or between 12 and 24 hours post-starvation (Fig 1G). Indeed, whole-body triglyceride levels in starved females remained at 77% of the levels found in fed female flies by 24 hours post-starvation, a time when triglyceride levels in starved males were at only 4% of the levels found in fed males (S1F Fig). Consistent with our data from *CS* flies, we observed a rapid drop in triglyceride levels post-starvation in *w*^*1118*^ virgin males compared with females (Fig 1H), demonstrating that the sexual dimorphism in triglyceride breakdown exists in multiple genetic backgrounds. To determine whether male and female gonads play a role in the sexual dimorphism in triglyceride breakdown, we first measured triglyceride levels post-starvation in ovaries and testes dissected from 5-day-old *w*^*1118*^ males and females. We found that triglyceride levels were unchanged by starvation in both organs (S1A and S1B Fig), suggesting that triglyceride levels in the gonads do not fully account for the male–female difference in triglyceride breakdown. Moreover, when we measured triglyceride levels post-starvation in *w*^*1118*^ male and female carcasses devoid of gonads, we found that triglyceride levels did not change in female carcasses between 0 and 12 hours post-starvation, whereas there was a significant decrease in triglyceride levels in male carcasses during this same interval (S1G Fig). Thus, in addition to the male–female difference in triglyceride storage, our findings reveal a sexual dimorphism in triglyceride breakdown.

### Sexual dimorphism in metabolic rate and macronutrient utilization

**Fig 2:**
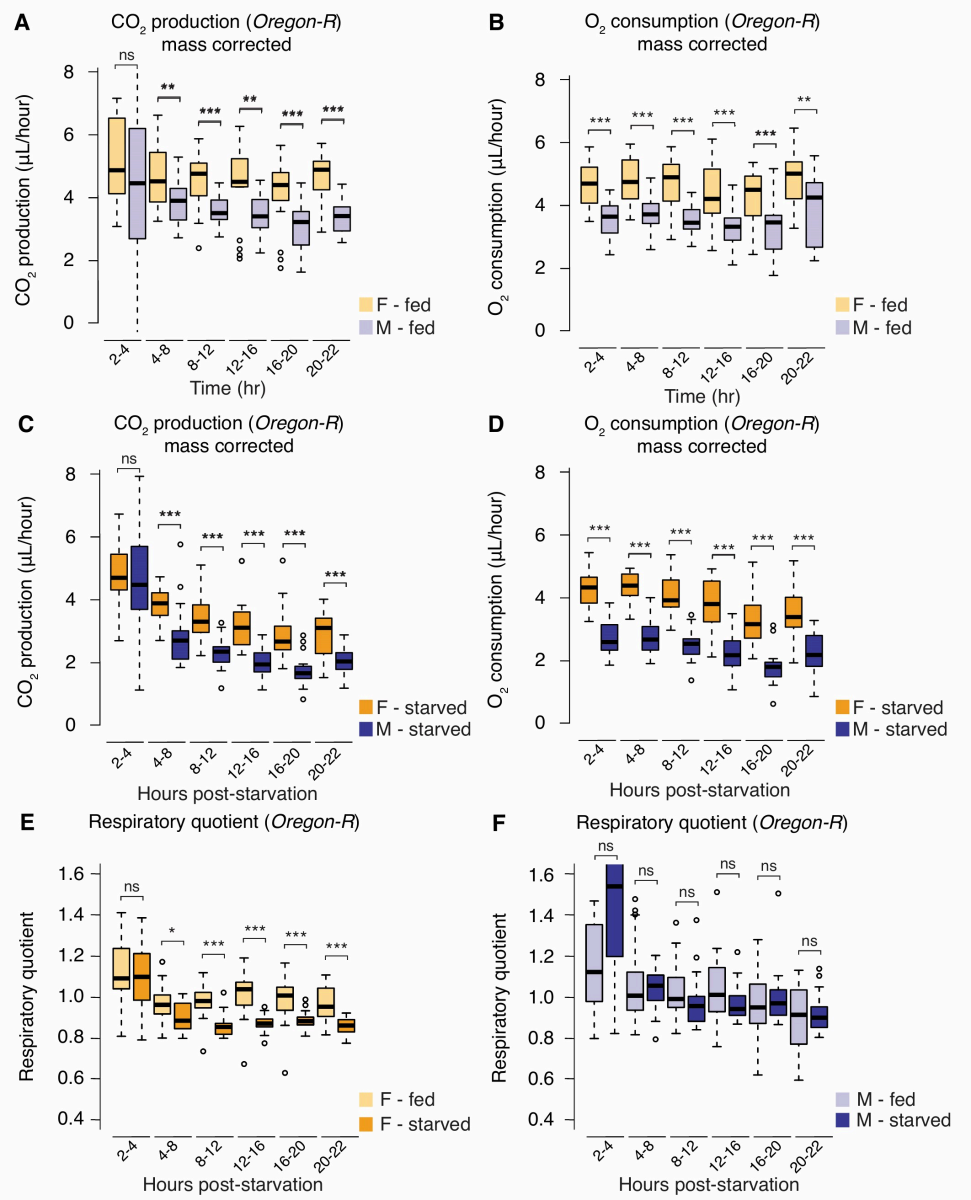
Sex differences in metabolic rate and macronutrient utilization. (A) Mass-corrected CO_2_ production was significantly higher in fed *Oregon-R* virgin females than in virgin males for most intervals during the observation period (*p* = 0.18, 4.5 × 10^−3^, 5.2 × 10^−5^, 4.9 × 10^−3^, 6.6 × 10^−4^, 2.43 × 10^−7^, respectively; Student *t* test at each time point). (B) Mass-corrected O_2_ consumption was significantly higher at each interval in fed *Oregon-R* females than in males during the observation period (*p* = 9.8 × 10^−6^, 1.8 × 10^−6^, 2.2 × 10^−6^, 5.2 × 10^−4^, 7.9 × 10^−4^, 4.3 × 10^−3^, respectively; Student *t* test at each time point). (C) Mass-corrected CO_2_ production post-starvation was significantly higher in females than in males for most intervals during the observation period (*p* = 0.55, 3.5 × 10^−4^, 6.4 × 10^−7^, 2.7 × 10^−6^, 8.0 × 10^−6^, 5.9 × 10^−5^, respectively; Student *t* test at each time interval). (D) Mass-corrected O_2_ consumption post-starvation was significantly higher in females at all intervals during the observation period (*p* = 2.4 × 10^−10^, 3.4 × 10^−11^, 1.4 × 10^−10^, 1.9 × 10^−8^, 1.1 × 10^−7^, 2.5 × 10^−6^, respectively; Student *t* test at each time interval). (E) The RQ was calculated as the ratio between CO_2_ production to O_2_ consumption at defined intervals over a 24-hour observation period in 5-day-old *Oregon-R* virgin females and males that were placed on either standard media or starvation media. In starved females, we observed a significant reduction in RQ compared with control females on standard media from 4 to 8 hours post-starvation onward (*p* = 0.85, 0.014, 6.5 × 10^−6^, 1.3 × 10^−5^, 8 × 10^−4^, 2.2 × 10^−5^, respectively; Student *t* test at each time point). (F) In male flies, we observed no significant change in RQ compared with control males on standard medium at any time during the observation period (*p* = 0.066, 0.89, 0.24, 0.079, 0.39, 0.62, respectively; Student *t* test at each time point). For indirect calorimetry measurements, the *p*-values are listed in the following order: difference between fed and starved animals at 2–4 hours, 4–8 hours, 8–12 hours, 12–16 hours, 16–20 hours, and 20–22 hours. Asterisks indicate a significant difference between two sexes, two genotypes, or two time points (*p < 0.05, **p < 0.01, ***p < 0.001). Error bars on graphs represent SEM. Quantitative measurements underlying all graphs are available in S2 Data. F, female; M, male; ns, no significant difference between two sexes, two genotypes, or time points; RQ, respiratory quotient.

One potential explanation for increased triglyceride storage and reduced triglyceride breakdown post-starvation in females is a lower demand for energy from physical activity or from basal metabolic processes. Because previous studies have shown that female flies are active over a larger portion of the day than males [42,43], we used indirect calorimetry to determine whether females have a lower energy demand due to basal metabolic processes under normal culture conditions and in response to starvation. In 5-day-old adults, mass-corrected CO_2_ production and O_2_ consumption were significantly higher in virgin females than in age-matched virgin males throughout the 24-hour monitoring window (Fig 2A and 2B; see also S2A and S2B Fig for non-mass-corrected data). This sex difference in CO_2_ production and O_2_ consumption persisted post-starvation (Fig 2C and 2D; see S2C and S2D Fig for non-mass-corrected data): although both females and males demonstrated a significant reduction in metabolic rate from 4 hours post-starvation until the end of the 24-hour observation period (S3A–S3D Fig), a change that was independent of any change in mass (S3E–S3H Fig), CO_2_ production and O_2_ consumption in virgin females remained significantly higher post-starvation than in virgin males. Taken together, these results do not support a model in which sexual dimorphism in triglyceride storage and breakdown are caused by lower energy demand in females.

We next asked whether the sex differences in triglyceride homeostasis might be due to male–female differences in the preferential use of macronutrients to fuel basal metabolic processes. We therefore calculated the respiratory quotient (RQ) from the ratio of CO_2_ production to O_2_ consumption in each sex. An RQ of 1 normally indicates the use of carbohydrates as the primary fuel for metabolic processes, and an RQ below 1 indicates a shift toward fat and protein utilization [44]. Under normal culture conditions, the RQ was approximately 1 in both virgin males and females (S4A Fig), indicating that both sexes are using similar macronutrients to fuel basal metabolic processes. Thus, the sexual dimorphism in triglyceride storage was not caused by a male–female difference in overall macronutrient usage under normal conditions. When we calculated the RQ at several time points post-starvation, we saw a significant difference between males and females (S4B Fig). In starved virgin females, we observed a significant decrease in RQ compared with fed virgin females from as early as 4 to 8 hours post-starvation, a change that persisted throughout our 24-hour observation period (Fig 2E). In contrast, the RQ in starved virgin males was not significantly different from fed control virgin males at any time throughout the 24-hour starvation period (Fig 2F). Interestingly, the decreased RQ in virgin females indicates a shift from carbohydrate fuel toward either fat and/or protein catabolism; however, we found no sexual dimorphism in protein breakdown and negligible differences in other macronutrients over the 24-hour starvation period (S5A–S5C Fig). The strong shift in RQ, indicating higher lipid catabolism in females, is surprising in light of our finding that triglyceride breakdown is lower in virgin female flies post-starvation. One possible explanation for this finding is that the amount of ATP generated by one fatty acid molecule is higher than for one molecule of glucose. Because females display a shift toward lipid as the main source of energy post-starvation, this may allow for sufficient ATP production post-starvation despite less overall triglyceride breakdown compared with males. Together, these findings highlight a significant difference in energy physiology between males and females and support a model in which there is a male–female difference in lipid catabolism post-starvation.

### Sex-biased gene expression of triglyceride metabolism genes

**Fig 3:**
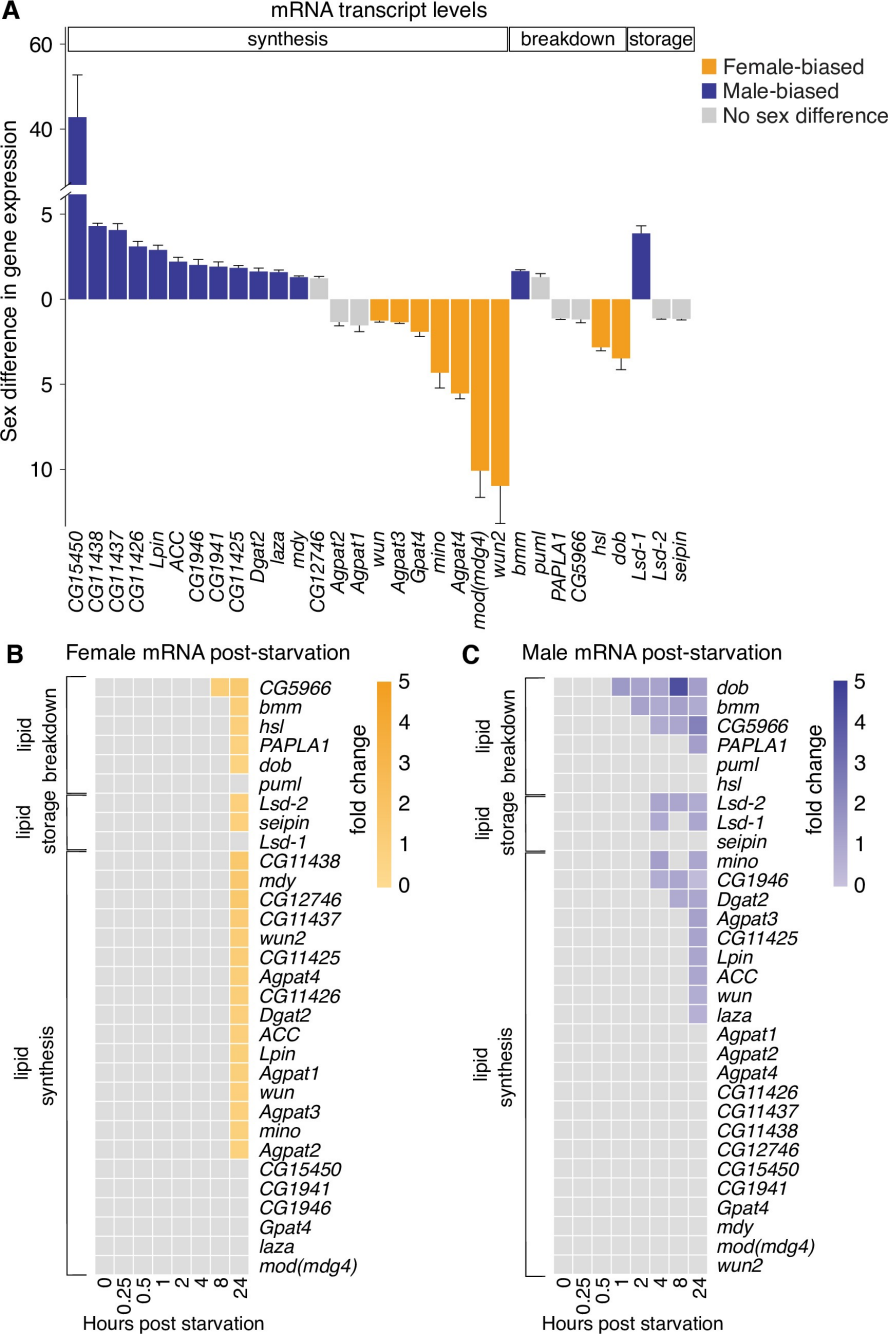
Extensive sex-biased expression of genes involved in maintaining triglyceride homeostasis. (A) Sex-biased mRNA levels of a panel of 31 genes known or predicted to be involved in triglyceride metabolism in 5-day-old virgin *w*^*1118*^ females and males. Gray-colored bars indicate no significant difference in mRNA level between the sexes. Orange-colored bars indicate that mRNA levels are significantly higher in virgin females than in virgin males. Purple-colored bars indicate that mRNA levels are significantly higher in virgin males than in virgin females. (B, C) mRNA levels of a panel of genes involved in triglyceride metabolism in virgin 5-day-old female *w*^*1118*^ flies (B) and virgin 5-day-old male *w*^*1118*^ flies (C) measured at different times post-starvation. Gray boxes indicate that mRNA levels were not significantly different from sex-matched, fed controls; colored boxes indicate that mRNA levels were significantly different from age-matched fed flies, and the intensity of the color corresponds to the fold change in mRNA level (refer to legend). Error bars on graphs represent SEM. See S1 Table for a list of all multiple comparisons and *p*-values; quantitative measurements underlying gene expression data are available in S3 Data. *w*, *white*.

In order to identify genes that contribute to the sexual dimorphisms in triglyceride storage and breakdown, we used quantitative real-time PCR (qPCR) to measure mRNA levels in a subset of genes known or predicted to be involved in lipid synthesis, breakdown, and storage [4,17,19]. Our investigation revealed sex-specific regulation of many genes in 5-day-old *w*^*1118*^ virgin female and male flies cultured under normal conditions: 23 out of 31 (74%) genes we examined showed a sex difference in mRNA levels (Fig 3A). For example, GPAT enzyme *mino*, AGPAT enzyme *Agpat4*, and lipase *hsl* had strongly female-biased expression, whereas triglyceride lipase *bmm* and *lsd-1*/*PLIN1* mRNA levels were approximately 1.8- and 4-fold higher in males than in females, respectively. Some genes, such as AGPAT enzyme *Agpat1*, *lsd-2*/*PLIN2*, and *CG5966* showed no significant difference in mRNA level between the sexes (Fig 3A). Thus, under normal culture conditions, many genes known or predicted to affect triglyceride metabolism display strongly sex-biased expression, trends that persisted when a subset of genes was normalized to a different housekeeping gene (S6A and S6B Fig).

To gain insight into genes that may contribute to the sexual dimorphism in triglyceride breakdown, we measured mRNA levels in virgin males and females at various time points post-starvation. Because we observed a sex difference in phenotype by 12 hours post-starvation, we predicted that the majority of gene expression changes in males and females would precede this critical time point. In females, with the exception of *CG5966*, a gene that may be involved in triglyceride breakdown, no genes showed significant changes in mRNA expression until 24 hours post-starvation, a time at which most genes were significantly different from fed control females (Fig 3B). In males, we found significant changes to mRNA levels starting as early as 1 hour post-starvation (Fig 3C). For example, mRNA levels of predicted triglyceride lipase *dob* were significantly increased from 1 hour post-starvation onward; *bmm* mRNA levels were significantly up-regulated from 2 hours post-starvation onward; and *lsd-1*/*PLIN1*, DAGAT family member *CG1946*, and GPAT enzyme *mino* were significantly increased from 4 hours post-starvation onward (Fig 3C). Importantly, these trends persisted when we normalized a subset of genes to an additional housekeeping gene (S7A–S7L Fig). Taken together, these results demonstrate a marked sex difference in the transcriptional response to starvation: in males, there was a rapid transcriptional response as early as 1–2 hours post-starvation; in females, the transcriptional response was delayed: mRNA levels of most genes were not significantly different until 24 hours post-starvation.

### A role for triglyceride lipase *bmm* in the regulation of sex differences in fat storage and breakdown

**Fig 4:**
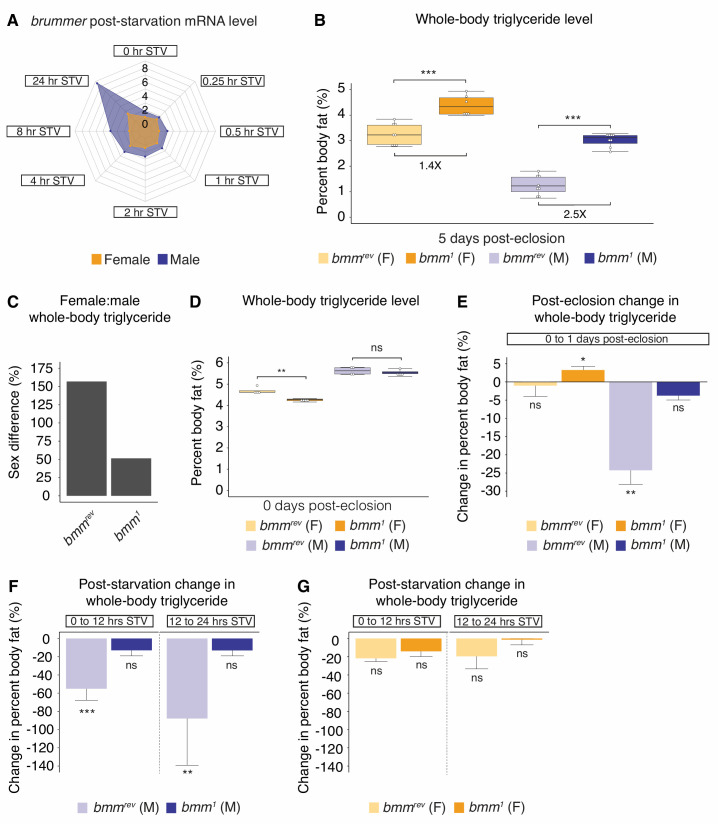
A role for *bmm* in the regulation of sex differences in triglyceride homeostasis. (A) Radar plot showing sex-specific regulation of *bmm* mRNA levels STV in 5-day-old virgin *w*^*1118*^ females and males STV. *bmm* mRNA levels were 1.8-fold higher in 5-day-old fed virgin males than in age-matched virgin females (*p* = 0.016; Student *t* test). At 4 hours STV, *bmm* mRNA levels were 1.6-fold higher in males than females (*p* = 0.019; Student *t* test). By 8 hours STV, mRNA levels were 3.1-fold higher in males than females (*p* = 8.6 × 10^−4^; Student *t* test). (B) Whole-body triglyceride storage was significantly higher in 5-day-old *bmm*^*1*^ homozygous mutant males compared with *bmm*^*rev*^ control males (*p* = 0; one-way ANOVA followed by Tukey HSD). Whole-body triglyceride storage was significantly increased in *bmm*^*1*^ homozygous mutant females compared with *bmm*^*rev*^ female controls (*p* = 1.9 × 10^−6^; one-way ANOVA followed by Tukey HSD). (C) The male-biased effect of *bmm* loss on triglyceride storage reduced the sexual dimorphism in triglyceride storage. (D) Whole-body triglyceride storage at eclosion was not significantly different between *bmm*^*1*^ mutant males compared with *bmm*^*rev*^ control males (*p* = 0.84; Student *t* test). (E) Triglyceride levels were lower in 1-day-old *bmm*^*rev*^ males compared with newly eclosed *bmm*^*rev*^ males (*p* = 0.0013; Student *t* test); however, there was no significant difference in whole-body triglyceride levels between 1-day-old *bmm*^*1*^ males and newly eclosed *bmm*^*1*^ males (*p* = 0.0793; Student *t* test). (F) In 5-day-old *bmm*^*rev*^ males, whole-body triglyceride storage significantly decreased between 0 and 12 hours STV, with a further reduction between 12 and 24 hours STV (*p* = 6.2 × 10^−5^ and 2.4 × 10^−3^, respectively; one-way ANOVA followed by Tukey HSD test). No significant change in whole-body triglyceride levels was observed in *bmm*^*1*^ mutant males between 0 and 12 hours STV, or between 12 and 24 hours STV (*p* = 0.244 and 0.349, respectively; one-way ANOVA followed by Tukey HSD test). (G) There was no significant change in whole-body triglyceride levels in either *bmm*^*rev*^ females or *bmm*^*1*^ females between 0 and 12 hours STV or between 12 and 24 hours STV (*p* = 0.0503 and 0.171 [0–12 hours], 0.244 and 0.998 [12–24 hours], respectively; one-way ANOVA followed by Tukey HSD test). Asterisks indicate a significant difference between two sexes, two genotypes, or two time points (*p < 0.05, **p < 0.01, ***p < 0.001). Error bars on graphs depicting percent body fat represent SEM; error bars on graphs depicting the change in percent body fat represent COE. See S1 Table for a list of all multiple comparisons and *p*-values; quantitative measurements for all data presented are available in S1 and S3 Datas. *bmm*, *brummer*; COE, coefficient of error; F, female; HSD, honest significant difference; M, male; ns, no significant difference between two sexes, two genotypes, or time points; STV, post-starvation; *w*, *white*.

To determine whether any triglyceride metabolism genes contribute to the male–female differences in triglyceride storage and breakdown, we wanted to investigate how individual genes contribute to the sex differences in triglyceride homeostasis. For most genes with sex-biased expression under normal culture conditions (Fig 3A), the magnitude of the sex bias in gene expression remained largely consistent throughout the starvation period (see S8A–S8D Fig for graphs of representative genes). One exception to this trend was triglyceride lipase *bmm*, which showed sex-specific regulation during normal culture conditions (1.8-fold higher in males) and a strong male-specific increase in mRNA levels post-starvation (3.1-fold higher in males by 8 hours post-starvation) (Fig 4A). Given that *bmm* regulation is highly sex specific under both normal culture conditions and post-starvation and that changes to *bmm* expression are known to influence whole-body triglyceride homeostasis [32], we reasoned that *bmm* may play a role in regulating sex differences in triglyceride storage and breakdown. Therefore, we compared triglyceride homeostasis in *bmm*^*1*^ mutant flies to *bmm*^*rev*^ control flies. Because the sex differences in triglyceride storage in *bmm*^*rev*^ flies were perfectly in line with our observations in *CS* and *w*^*1118*^ flies (S2 Table) and because *bmm*^*rev*^ flies and *bmm*^*1*^ mutant flies were derived from the same parental strain [32], this will allow us to investigate whether there is a role for *bmm* in the regulation of sex differences in triglyceride storage and breakdown. In accordance with previous reports [32], triglyceride levels in *bmm*^*1*^ homozygous mutant males were approximately 2.5 times higher than in *bmm*^*rev*^ control males (Fig 4B). In *bmm*^*1*^ mutant females, however, triglyceride storage was increased by only 1.4 times compared with *bmm*^*rev*^ control females (Fig 4B), demonstrating a strongly male-biased effect of *bmm* loss on triglyceride storage. Given that 5-day-old *bmm*^*1*^ mutant females fed a high-fat diet had significantly higher triglyceride levels than *bmm*^*1*^ females fed a normal diet (S9A Fig), the mild increase in triglyceride storage in *bmm*^*1*^ mutant females cannot be attributed to these females achieving a physiological limit of triglyceride storage. In fact, the remaining difference between *bmm*^*1*^ mutant females and males was due to the modest amount of triglyceride stored in the ovary, as no sex difference in triglyceride storage remained between *bmm*^*1*^ mutant females lacking ovaries and *bmm*^*1*^ mutant males (S9B Fig). Importantly, we reproduced these male-biased effects of reduced *bmm* function on triglyceride storage in flies with ubiquitous overexpression of two different *upstream activation sequence* (*UAS*)*-bmm-RNAi* lines driven by *daughterless* (*da*)-*GAL4* (S10A–S10F Fig). These strongly male-biased effects of *bmm* loss on triglyceride storage reduced the sexual dimorphism in triglyceride storage in *bmm*^*1*^ mutants compared with *bmm*^*rev*^ control flies (Fig 4C) and in *da>UAS-bmm-RNAi* flies compared with *da>+* and *+>UAS-bmm-RNAi* controls (S10C and S10F Fig). Together, these data suggest that normal *bmm* function plays a role in the regulation of sexual dimorphism in *Drosophila* triglyceride storage.

*bmm* is an essential gene for embryogenesis [32], in which maternally provided *bmm* allows the survival of *bmm*^*1*^ mutants past this critical period in development. We identified two ways that loss of *bmm* may influence the sex difference in triglyceride storage: first, by increasing the amount of larval fat stored in males during development; or second, by blocking the progressive reduction in body fat over the first 5 days of adult life (Fig 1E). To distinguish between these possibilities, we measured triglyceride levels in *bmm*^*1*^ mutants compared with *bmm*^*rev*^ controls at eclosion and in 1-day-old flies. We found no significant difference in whole-body triglyceride storage in newly eclosed flies between *bmm*^*1*^ mutants and *bmm*^*rev*^ control males (Fig 4D), suggesting that *bmm* does not contribute to the sex difference in triglyceride storage by enhancing fat storage in males during larval development. Instead, the rapid decrease in triglyceride storage normally observed in *bmm*^*rev*^ control males between 0 DPE and 1 DPE was blocked in *bmm*^*1*^ mutant males (Fig 4E). In further support of a role for *bmm* in mediating the male-specific decrease in triglyceride levels over the first few days post-eclosion, we observed an increase in *bmm* mRNA levels between 0- and 5-day-old flies in males but not in females (S10G Fig). We therefore propose that *bmm* plays a role in regulating the sex difference in triglyceride storage by promoting lipolysis in males during the first 5 days of adult life.

To determine whether *bmm* also plays a role in regulating the sex difference in triglyceride breakdown, we measured triglyceride breakdown post-starvation in 5-day-old *bmm*^*1*^ mutant virgin males and females and in *bmm*^*rev*^ control males and females. In control *bmm*^*rev*^ male flies, there was a significant decrease in whole-body triglyceride levels between both 0 and 12 hours post-starvation and between 12 and 24 hours post-starvation (Fig 4F), consistent with our findings in *CS* and *w*^*1118*^ virgin males. In *bmm*^*1*^ mutant males, however, the reduction in triglyceride levels between 0 and 12 hours post-starvation and between 12 and 24 hours post-starvation was abolished (Fig 4F), indicating that *bmm* promotes lipolysis post-starvation in males, as previously reported [32]. In contrast, there was no significant reduction in triglyceride levels between either 0 and 12 hours post-starvation or between 12 and 24 hours post-starvation in either *bmm*^*rev*^ or *bmm*^*1*^ mutant females (Fig 4G). These male-specific effects on triglyceride breakdown were reproduced in flies with *da-GAL4*-mediated expression of two independent *UAS-bmm-RNAi* lines compared with *da>+* and *+>UAS-bmm-RNAi* controls (S10H–S10K Fig), further supporting a role for *bmm* in regulating the sex difference in triglyceride breakdown post-starvation. Because of this male-specific effect of *bmm* loss on triglyceride breakdown, the sex difference in triglyceride breakdown was abolished. Together, these results identify novel roles for triglyceride lipase *bmm* in the regulation of sex differences in *Drosophila* triglyceride storage and breakdown.

### *bmm* function in the somatic cells of the gonad plays a role in regulating the sexual dimorphism in whole-body triglyceride homeostasis

Given that sex differences exist in many tissues throughout the fly [45–55], we wanted to determine the anatomical focus of *bmm*’s effects on the male–female differences in triglyceride homeostasis. *bmm* is highly expressed in the fat body, and previous studies have demonstrated a central role for this tissue in the regulation of triglyceride storage and breakdown in males [29,32]. We therefore compared triglyceride storage and breakdown in virgin males and females with *bmm* inhibition in the fat body. We chose *collagen* (*cg*)*-GAL4* and *r4-GAL4* to overexpress *UAS-bmm-RNAi* in the fat body because these drivers have been used in many fat body studies. We confirm that both GAL4 lines drive strong green fluorescent protein (GFP) expression in the abdominal fat body and have very weak expression in the gonad (S3 Table). In line with previous studies [56], triglyceride storage in *cg>UAS-bmm-RNAi* males was significantly higher than in *cg>+* and *+>UAS-bmm-RNAi* control males (S11A Fig). In females, triglyceride levels in *cg>UAS-bmm-RNAi* females were significantly higher than in *cg>+* and *+>UAS-bmm-RNAi* controls (S11B Fig). Because the increase in triglyceride storage upon *bmm* loss in the abdominal fat body was similar in both sexes, the sex difference in triglyceride storage between *cg>UAS-bmm-RNAi* males and females was unchanged. Likewise, because *bmm* inhibition in the abdominal fat body significantly delayed triglyceride breakdown in both sexes, the sex difference in triglyceride breakdown post-starvation remained (S11C and S11D Fig). When we repeated these experiments with *r4-GAL4*, we observed a male-specific increase in triglyceride storage in *r4>UAS-bmm-RNAi* males compared with *r4>+* and *+>UAS-bmm-RNAi* controls (S11E and S11F Fig); however, *r4-GAL4*-mediated loss of *bmm* in the fat body significantly delayed triglyceride breakdown in both sexes (S11G and S11H Fig). *bmm* loss in the abdominal fat body, therefore, does not fully account for the strongly male-biased effects of whole-body *bmm* loss on the sex differences in triglyceride storage and breakdown. Thus, despite a role for fat body *bmm* in maintaining triglyceride homeostasis in each sex, *bmm* function in additional cell types or tissues must also contribute to the sex differences in triglyceride homeostasis.

**Fig 5:**
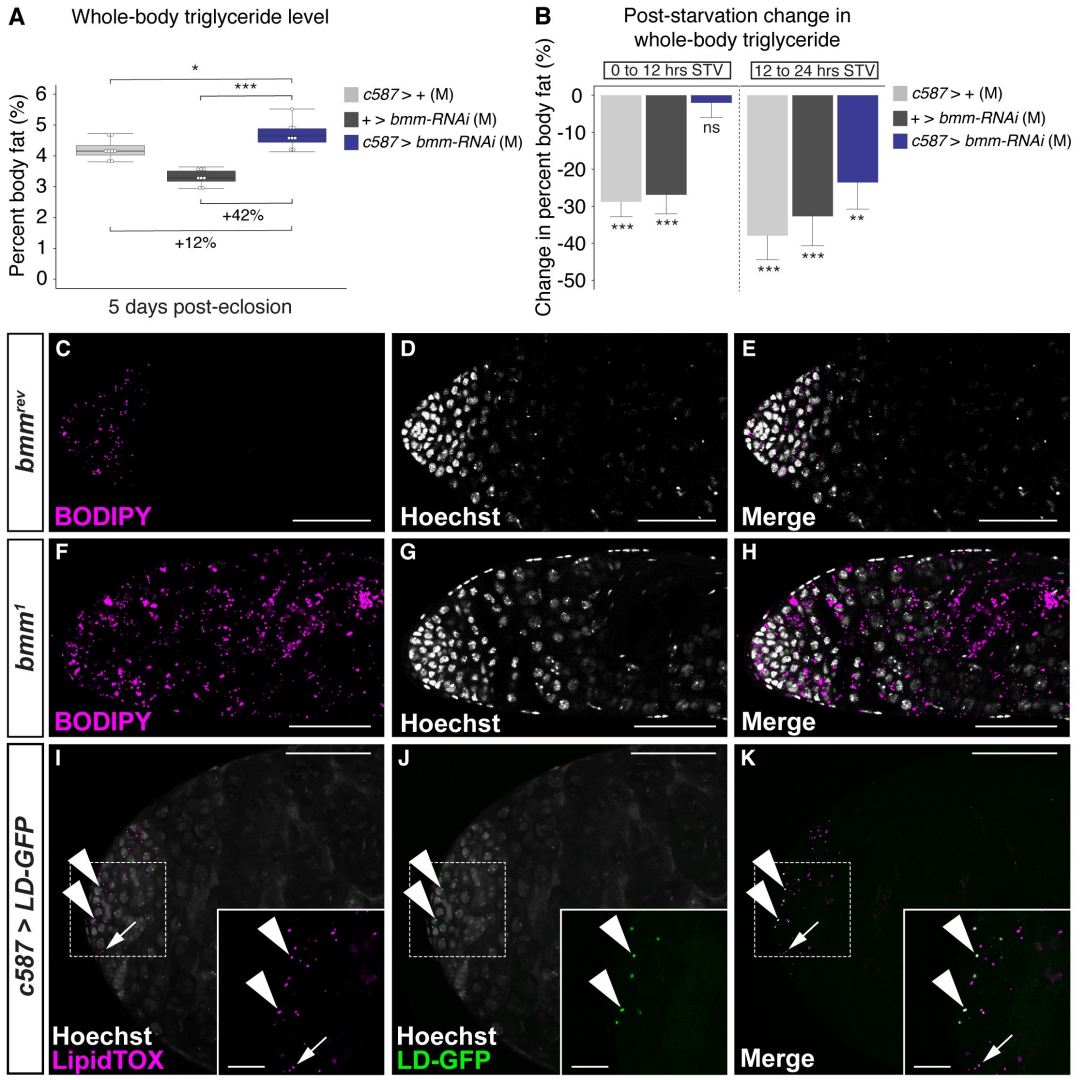
A role for *bmm* function in the somatic cells of the gonad in the regulation of whole-body triglyceride storage and breakdown in males. (A) Whole-body triglyceride storage in males overexpressing the *UAS-bmm-RNAi* transgene in the somatic cells of the male gonad (*c587>UAS-bmm-RNAi*) was significantly higher than in control males (*c587>+* and *+>UAS-bmm-RNAi*) (*p* = 0.027 and 2 × 10^−7^, respectively; one-way ANOVA followed by Tukey HSD test). (B) Whole-body triglyceride levels in *c587>+* and *+>UAS-bmm-RNAi* control males showed a significant decrease between 0 and 12 hours STV (1 × 10^−7^ and 1.1 × 10^−6^, respectively; one-way ANOVA followed by Tukey HSD test), whereas triglyceride levels were not significantly different between 0 and 12 hours STV in *c587>UAS-bmm-RNAi* males (*p* = 0.997; one-way ANOVA followed by Tukey HSD test). (C–H) We detected lipid droplets in testes dissected from 0-day-old *bmm*^*1*^ and *bmm*^*rev*^ virgin male flies using BODIPY, a neutral lipid stain. Dissected testis from 0-day-old virgin *bmm*^*1*^ mutant males (F–H) show a dramatic increase in lipid droplets compared with control *bmm*^*rev*^ males (C–E). (I–K) Using an LD-GFP, we found that a subset of the LipidTOX-positive lipid droplets in the testis (arrowheads) represent droplets in the somatic cells of the gonad. Non-GFP-positive droplets (arrow) likely represent lipid droplets in the germline, another cell type in the testis. Scale bars = 50 μm, except for inset images for (I–K), in which scale bars = 12.5 μm. The *p*-values are listed in the following order: difference between the *GAL4*/*UAS* genotype and the *GAL4* control/difference between the *GAL4*/*UAS* genotype and the *UAS* control. Asterisks indicate a significant difference between two sexes, two genotypes, or two time points (*p < 0.05, **p < 0.01, ***p < 0.001). Error bars on graphs depicting percent body fat represent SEM; error bars on graphs depicting the change in percent body fat represent COE. See S1 Table for a list of all multiple comparisons and *p*-values; quantitative measurements underlying data presented in the figure are available in S1 Data. Original image files corresponding to all images acquired from genotype-matched individuals presented in panels C–K are available upon request. *bmm*, *brummer*; BODIPY, boron-dipyrromethene; COE, coefficient of error; GFP, green fluorescent protein; HSD, honest significant difference; LD-GFP, lipid droplet–targeted GFP; M, male; ns indicates no significant difference between two sexes, two genotypes, or time points; STV, post-starvation; *UAS*, *upstream activation sequence*.

In addition to the fat body, *bmm* mRNA is present in the *Drosophila* intestine, central nervous system (CNS), muscle, neurons, glia, ovary, and testis [55]. To identify additional tissues in which *bmm* function is required to maintain triglyceride homeostasis, we measured triglyceride storage and breakdown in virgin females and males with RNAi-mediated inhibition of *bmm* in several cell types and tissues. Loss of *bmm* in the gut, muscles, and glia had no effect on triglyceride levels in either sex under normal culture conditions (S12A–S12F Fig). However, we identified two additional cell types in which loss of *bmm* function caused significant changes to whole-body triglyceride homeostasis. Using *c587-GAL4*, a driver with strong expression in the somatic cells of the gonad and in a limited number of neurons (S3 Table), we observed a change in whole-body triglyceride storage. Triglyceride levels in *c587>UAS-bmm-RNAi* males were significantly higher than in *c587>+* and *+>UAS-bmm-RNAi* control males (Fig 5A). In addition, triglyceride breakdown was also significantly delayed in *c587>UAS-bmm-RNAi* males compared with *c587>+* and *+>UAS-bmm-RNAi* control males (Fig 5B). These effects on male triglyceride storage and breakdown were specific to *bmm*, as we observed similar results when we used an additional RNAi line to knock down *bmm* function (S13A and S13B Fig), and we rescued both the increased triglyceride storage and reduced triglyceride breakdown by simultaneous overexpression of *UAS-bmm-RNAi* and *UAS-bmm* in the somatic cells of the gonad (S13C and S13D Fig). In females, there were no significant effects on either triglyceride storage or triglyceride breakdown in *c587>UAS-bmm-RNAi* females compared with *c587>+* and *+>UAS-bmm-RNAi* controls for multiple RNAi lines (S14A–S14D Fig), and no significant effect of *c587-GAL4*-mediated coexpression of the *UAS-bmm-RNAi* and *UAS-bmm* transgenes compared with controls (S14E and S14F Fig). Because of these male-specific effects on triglyceride storage and breakdown, the sex differences in triglyceride storage and triglyceride breakdown were reduced. When we used *traffic jam (tj)-GAL4*, a line with expression in the somatic cells of the gonad and in a small number of neurons (S15A–S15D Fig and S3 Table), we reproduced the male-specific effects of *bmm* inhibition on triglyceride breakdown that we observed with *c587-GAL4*-mediated *bmm* inhibition (S15C and S15D Fig). Although we observed no change in whole-body triglyceride storage between *tj>UAS-bmm-RNAi* animals and controls in either sex (S15A and S15B Fig), this may reflect minor differences in the strength or timing of expression between *c587-GAL4* and *tj-GAL4*. Because neither *c587-GAL4* nor *tj-GAL4* drives GFP expression in the fat body (S3 Table), these results suggest a role for *bmm* in the somatic cells of the gonad in regulating whole-body triglyceride storage and breakdown in males.

Although the somatic cells of the gonad have not previously been shown to be an important site for triglyceride storage, high-throughput data sets have detected *bmm* mRNA in the *Drosophila* male testis [55], and *bmm*’s mammalian homolog *adipose triglyceride lipase* (*ATGL*) is present in the murine testis [57,58]. In addition, we show that lipid droplets, a cytoplasmic organelle in which triglycerides are stored and *bmm* protein localizes [32], are present in the *Drosophila* testis (Fig 5C–5E). To determine whether loss of *bmm* affects triglyceride homeostasis in this tissue, we examined lipid droplets in the testis and found a clear increase in the number of lipid droplets in testes isolated from *bmm*^*1*^ males (Fig 5F–5H) compared with control *bmm*^*rev*^ males (Fig 5C–5E). The *Drosophila* testis contains two cell types: the germline cells and the somatic support cells [59]. To determine which cell type contains the lipid droplets, we first took high-magnification images of lipid droplets in the testis of males in which the somatic cells express membrane-bound GFP (*tj>UAS-mCD8*::*GFP*). Given that some of the lipid droplets are present within the GFP boundary of the cell (arrowheads), this suggests that lipid droplets are present in somatic cells (S15E–S15G Fig). To further confirm the presence of lipid droplets in somatic cells, we overexpressed a transgene encoding a lipid droplet–targeted GFP (LD-GFP) in the somatic cells of the gonad [60]. Previous work identified the lipid droplet–targeting domain in transport regulator protein Klarsicht (Klar; FBgn0001316) [60]. When this Klar lipid droplet–targeting domain was fused to GFP (LD-GFP), the LD-GFP fusion protein localized to the surface of lipid droplets [60]. Thus, by expressing *UAS-LD-GFP* in a cell type of interest, lipid droplets in that cell type will be positively marked with LD-GFP [60]. In testes dissected from *c587>UAS-LD-GFP* males, we detected several GFP-positive punctae that were also positive for LipidTOX Red, a neutral lipid dye that marks lipid droplets (Fig 5I–5K). Because *c587-GAL4* does not drive transgene expression in the germline [61], the presence of punctae that are both GFP and LipidTOX Red positive in the testis confirms that lipid droplets are present in the somatic cells of the *Drosophila* gonad. Together, our results identify a new role for *bmm* in regulating lipid droplets in the *Drosophila* testis and suggest a role for *bmm* in the somatic cells of the male gonad in the regulation of whole-body triglyceride storage and breakdown.

### A role for *bmm* function in neurons in the regulation of sex differences in triglyceride breakdown

**Fig 6:**
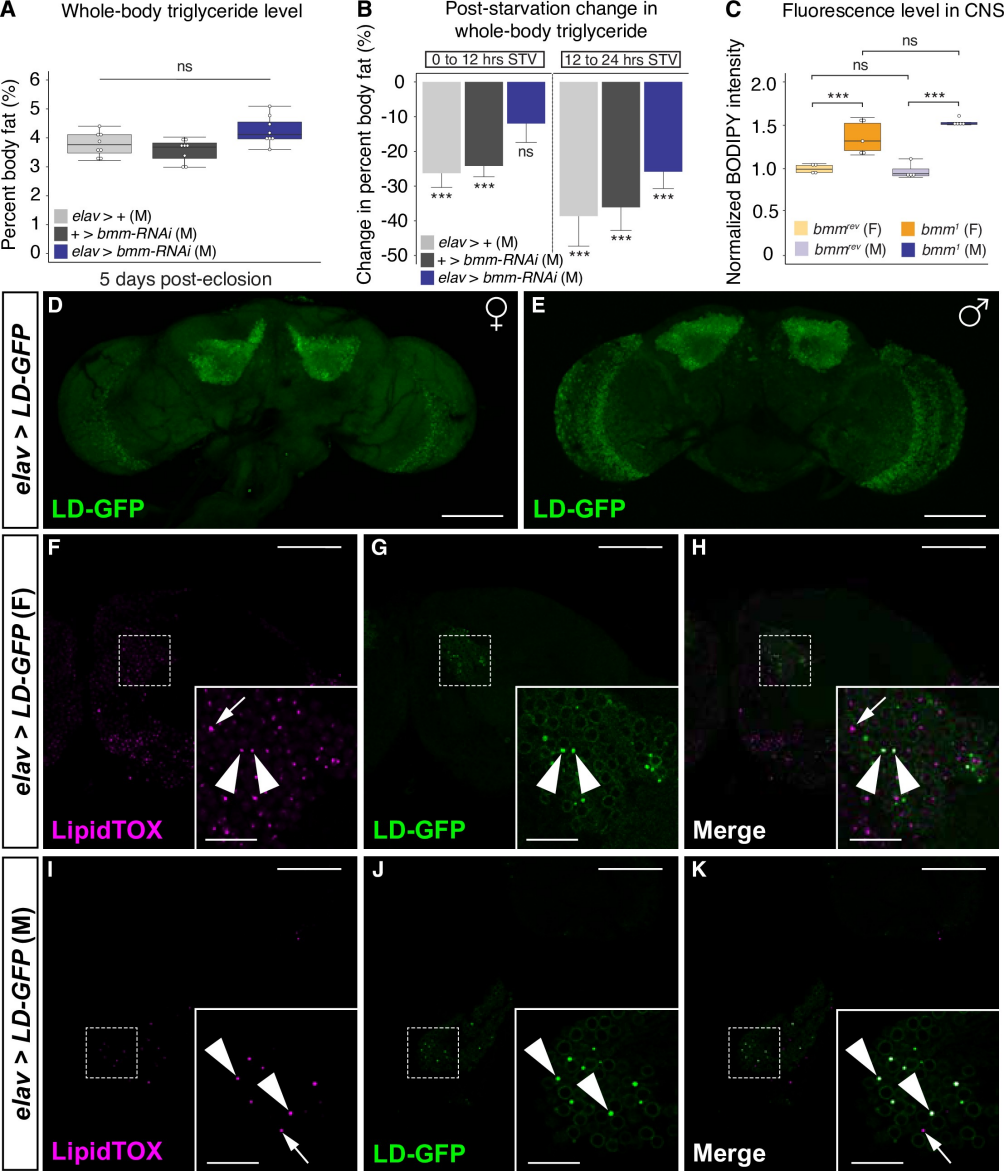
A role for *bmm* function in neurons in the regulation of whole-body triglyceride breakdown in males. (A) Whole-body triglyceride storage in 5-day-old virgin males overexpressing *UAS-bmm-RNAi* in postmitotic neurons (*elav>UAS-bmm-RNAi*) was not significantly different from age-matched control males (*elav>+* and *+>UAS-bmm-RNAi*) (*p* = 0.095 and 0.011; one-way ANOVA followed by Tukey HSD test). (B) There was a significant reduction in whole-body triglyceride levels in 5-day-old *elav>+* and *+>UAS-bmm-RNAi* control males between 0 and 12 hours STV (*p* = 1 × 10^−5^ and 9 × 10^−6^, respectively; one-way ANOVA followed by Tukey HSD test); however, no significant decrease in triglyceride levels was observed between 0 and 12 hours STV in *elav>UAS-bmm-RNAi* males (*p* = 0.13; one-way ANOVA followed by Tukey HSD test). (C) In both sexes, lipid droplet–derived fluorescence in dissected *Drosophila* brains was significantly higher in 5-day-old *bmm*^*1*^ mutants compared with *bmm*^*rev*^ controls (*p* = 2.5 × 10^−5^ and 0.002 in males and females, respectively; one-way ANOVA followed by Tukey HSD). (D, E) Expression of an LD-GFP transgene in neurons revealed GFP-positive punctae throughout the *Drosophila* CNS in females (D) and males (E). Maximum Z-projections, dorsal view, anterior up. Scale bars = 100 μm. (F–K) Expression of LD-GFP in neurons revealed that a subset of the LipidTOX-positive lipid droplets found in the CNS of 5-day-old adult females (F–H) and males (I–K) represent droplets in neurons (arrowheads). (F–K) Non-GFP-positive droplets likely represent lipid droplets in glia, another cell type in the CNS (arrow). White boxes indicate area magnified in inset. Single confocal slice through the *Drosophila* brain, dorsal view, anterior up. Scale bars = 50 μm; scale bars = 12.5 μm in magnified inset images. The *p*-values are listed in the following order: difference between the *GAL4*/*UAS* genotype and the *GAL4* control/difference between the *GAL4*/*UAS* genotype and the *UAS* control. Asterisks indicate a significant difference between two sexes, two genotypes, or two time points (*p < 0.05, **p < 0.01, ***p < 0.001). Error bars on graphs depicting percent body fat or BODIPY intensity represent SEM; error bars on graphs depicting the change in percent body fat represent COE. See S1 Table for a list of all multiple comparisons and *p*-values; quantitative measurements for all data are available in S1 Data. Original image files corresponding to all images acquired from genotype-matched individuals presented in panels D–K are available upon request. *bmm*, *brummer*; BODIPY, boron-dipyrromethene; CNS, central nervous system; COE, coefficient of error; *elav*, *embryonic lethal abnormal vision*; F, female; GFP, green fluorescent protein; HSD, honest significant difference; LD-GFP, lipid droplet–targeted GFP; M, male; ns, no significant difference between two sexes, two genotypes, or time points; STV, post-starvation; *UAS*, *upstream activation sequence*.

In addition to the somatic cells of the gonad, we found a role for *bmm* function in neurons in regulating the sex difference in triglyceride breakdown. We used *embryonic lethal abnormal vision* (*elav)-GAL4* to overexpress the *UAS*-*bmm-RNAi* transgene in postmitotic neurons. Under normal culture conditions, we saw no significant increase in whole-body triglyceride storage in *elav>UAS-bmm-RNAi* males compared with *elav>+* and *+>UAS-bmm-RNAi* control males (Fig 6A), a finding we confirmed using an independent *UAS-bmm-RNAi* line (S16A Fig) and an independent GAL4 line for neurons (*neuronal Synaptobrevin* [*nSyb*]*-GAL4*) (S16B Fig). When we measured triglyceride levels post-starvation, however, we observed a significant delay in triglyceride breakdown. In *elav>+* and *+>UAS-bmm-RNAi* control males, triglyceride levels were significantly lower by 12 hours post-starvation compared with genotype-matched, fed control males (Fig 6B). In contrast, there was no significant reduction in whole-body triglyceride levels between 0 and 12 hours post-starvation in *elav>UAS-bmm-RNAi* males (Fig 6B), a finding we confirmed using an independent *UAS-bmm-RNAi* line (S16C Fig) and an independent neuronal GAL4 line (*nSyb-GAL4*) (S16D Fig). Moreover, we show that *bmm* transcript levels are nearly undetectable in dissected brains from *elav-GAL4>UAS-bmm-RNAi* males compared with control males (S16E Fig) and that simultaneous overexpression of *UAS-bmm* together with *UAS-bmm-RNAi* in neurons rescued the delay in triglyceride breakdown between 0 and 12 hours post-starvation (S16F and S16G Fig). This delay in triglyceride breakdown was largely restricted to early time points post-starvation because the decrease in triglyceride levels in *elav>UAS-bmm-RNAi* males between 12 and 24 hours post-starvation was in line with the reduction we observed in control males during this interval (Fig 6B). Moreover, the delay in triglyceride breakdown was specific to neurons, as triglyceride breakdown in males with loss of *bmm* in glia was indistinguishable from control males (S17A and S17B Fig). Together, these data provide strong evidence of a role for *bmm* in male neurons in regulating triglyceride breakdown. In females, neither triglyceride storage nor triglyceride breakdown was significantly different between *elav>UAS-bmm-RNAi* females and *elav>+* and *+>UAS-bmm-RNAi* control females for either of our RNAi lines (S18A–S18D Fig), between *nSyb>UAS-bmm-RNAi* females and controls (S18E and S18F Fig), or between *elav>UAS-bmm;UAS-bmm-RNAi* females and controls (S18G and S18H Fig). Overall, these male-specific effects reduced the sex difference in triglyceride breakdown, identifying a novel role for *bmm* in male neurons in the regulation of lipolysis post-starvation.

Although the *Drosophila* CNS is not a major site for triglyceride storage, lipids play an essential role in neuronal and glial function [62–64]. Furthermore, a recent single-cell RNA-sequencing analysis of the *Drosophila* brain confirms that *bmm* mRNA is present within both neurons and glia [65], and previous reports identified lipid droplets in the retina of adult flies [66]. To determine whether *bmm* contributes to the regulation of lipid droplets in this tissue, we measured the intensity of lipid droplet–derived fluorescence in the CNS of *bmm*^*1*^ mutants compared with *bmm*^*rev*^ controls. We found a significant increase in fluorescence in *bmm*^*1*^ mutant males and females compared with *bmm*^*rev*^ controls (Fig 6C), suggesting that *bmm* function normally limits neutral lipid accumulation in this tissue. Because the CNS is composed of multiple cell types that are not easily distinguished based on morphological characteristics, we used *elav-GAL4* to drive LD-GFP expression exclusively in neurons to determine whether lipid droplets are present in this cell type [60]. We found punctae that were both GFP and LipidTOX Red positive throughout the CNS in both males and females (Fig 6D–6K). Because the LD-GFP protein is present only in neurons, these GFP-positive punctae represent lipid droplets in neurons, confirming that lipid droplets are normally present in adult *Drosophila* neurons in both sexes. Thus, lipid droplets are present in neurons under normal physiological conditions, and *bmm* function in neurons plays a previously unrecognized role in stimulating whole-body triglyceride breakdown post-starvation in males.

### Loss of *bmm* affects life span and the sex difference in starvation resistance

**Fig 7:**
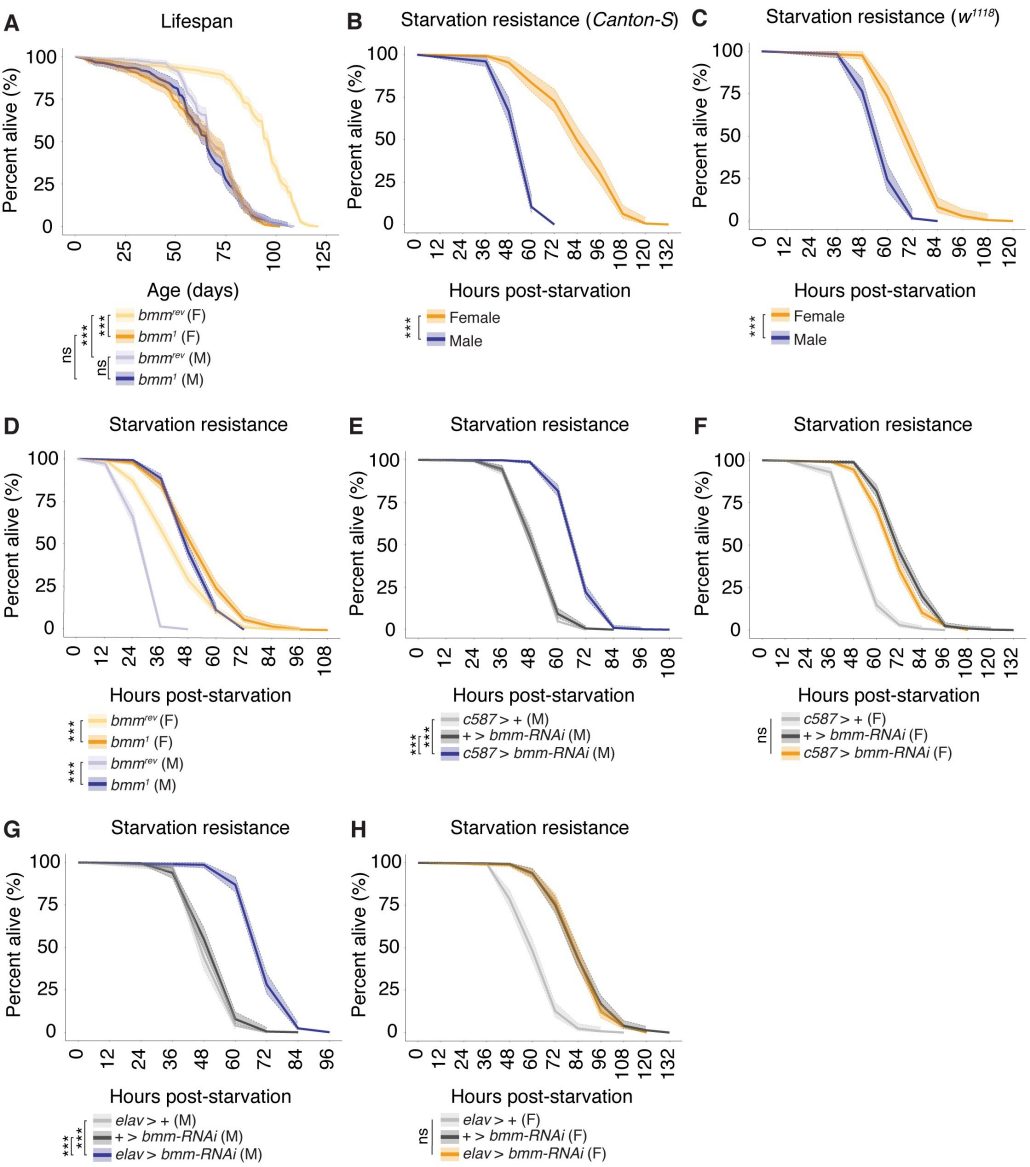
*bmm*-mediated regulation of triglyceride homeostasis affects life span and contributes to the sex difference in starvation resistance. (A) Median life span was significantly higher in *bmm*^*rev*^ virgin females than in *bmm*^*rev*^ virgin males (*p* = 2 × 10^−16^; Log-rank test with Bonferroni correction for multiple comparisons; *n* > 297 for all sexes and genotypes). Median life span was significantly reduced in *bmm*^*1*^ mutant females compared with *bmm*^*rev*^ control females (28-day reduction in survival, *p* = 2 × 10^−16^; Log-rank test with Bonferroni correction for multiple comparisons). No significant decrease was found in *bmm*^*1*^ mutant males compared with control males (*p* = 0.17; Log-rank test with Bonferroni correction for multiple comparisons). (B) Median survival post-starvation was significantly higher in 5-day-old virgin *Canton-S* females than in virgin *Canton-S* males (*p* = 2 × 10^−16^; Log-rank test with Bonferroni correction for multiple comparison; *n* > 154). (C) Median survival post-starvation was significantly higher in 5-day-old *w*^*1118*^ virgin females compared with *w*^*1118*^ virgin males (*p* = 2 × 10^−16^; Log-rank test with Bonferroni correction for multiple comparisons; *n* > 123). (D) Median survival post-starvation was significantly higher in 5-day-old *bmm*^*rev*^ virgin females than in *bmm*^*rev*^ virgin males (*p* = 2 × 10^−16^; Log-rank test with Bonferroni correction for multiple comparisons; *n* > 454 for both sexes and genotypes). Median survival post-starvation was significantly increased in male *bmm*^*1*^ mutants compared with *bmm*^*rev*^ control males (*p* = 2 × 10^−16^; Log-rank test with Bonferroni correction for multiple comparisons) and in *bmm*^*1*^ mutant females compared with *bmm*^*rev*^ controls (*p* = 2 × 10^−16^; Log-rank test with Bonferroni correction for multiple comparisons). The male-biased effects of *bmm* loss on starvation resistance reduced the sex difference in median survival. (E) Median survival was significantly higher in virgin males with *bmm* inhibition in somatic cells of the male gonad (*c587>UAS-bmm-RNAi*) compared with control males (*c587>+* and *+>UAS-bmm-RNAi*) (*p* = 2 × 10^−16^ and 2 × 10^−16^, respectively; Log-rank test with Bonferroni correction for multiple comparisons; *n* > 326 for all genotypes). (F) Median survival post-starvation was not significantly different in *c587>UAS-bmm-RNAi* virgin females compared with *c587>+* and *+>UAS-bmm-RNAi* control females (*p* = 2 × 10^−16^ and 1.5 × 10^−5^, respectively; Log-rank test with Bonferroni correction for multiple comparisons; *n* > 408 for all genotypes). (G) Median survival post-starvation was significantly higher in virgin males with *bmm* inhibition in postmitotic neurons (*elav>UAS-bmm-RNAi*) compared with *elav>+* and *+>UAS-bmm-RNAi* control males (*p* = 2 × 10^−16^ and 2 × 10^−16^, respectively; Log-rank test with Bonferroni correction for multiple comparisons; *n* > 178 for all genotypes). (H) Median survival post-starvation was not significantly higher in *elav>UAS-bmm-RNAi* virgin females compared with *elav>+* and *+>UAS-bmm-RNAi* control females (*p* = 2 × 10^−16^ and 1, respectively; Log-rank test with Bonferroni correction for multiple comparisons; *n* > 253 for all genotypes). The male-specific effects of *bmm* inhibition in neurons reduced the sex difference in median survival. The *p*-values are listed in the following order: difference between the *GAL4*/*UAS* genotype and the *GAL4* control/difference between the *GAL4*/*UAS* genotype and the *UAS* control. Asterisks indicate a significant difference between two sexes, two genotypes, or two time points (*p < 0.05, **p < 0.01, ***p < 0.001). Shaded areas represent the 95% confidence interval. See S1 Table for a list of all multiple comparisons and *p*-values; quantitative measurements corresponding to all data presented in the figure are available in S4 Data. *bmm*, *brummer*; *elav*, *embryonic lethal abnormal vision*; F, female; M, male; ns, no significant difference between two sexes, two genotypes, or time points; *UAS*, *upstream activation sequence*; *w*, *white*.

Previous studies have shown that the correct regulation of triglyceride homeostasis is important for a normal life span [32]. For example, life span was significantly reduced in *bmm*^*1*^ mutant males compared with *bmm*^*rev*^ control males [32]. In light of our findings that loss of *bmm* has male-biased effects on triglyceride homeostasis, we wanted to examine life span in both sexes because previous studies used only male flies [32]. In *bmm*^*rev*^ control females, median life span was 96 days, whereas in *bmm*^*1*^ mutant females, the median life span was only 68 days, a reduction of 29% (Fig 7A). This significant reduction in female life span was unexpected given the relatively modest increase in whole-body triglyceride level in *bmm*^*1*^ mutant females (Fig 4B). In *bmm*^*1*^ mutant males, we observed no significant reduction in life span in males (Fig 7A) despite a 2.5× increase in triglyceride storage (Fig 4B). Although this finding differs from the previously reported 10% reduction in life span in male *bmm*^*1*^ mutants [32], a difference likely due to minor interlaboratory variations in aging regime or diet [67], our study identifies an unexpected female-biased reduction in life span upon loss of *bmm*.

Another phenotype that is closely associated with the regulation of triglyceride homeostasis is starvation resistance [29–32,56,68,69]. In line with previous studies showing increased starvation resistance in mated females compared with males [38,70], we demonstrate that starvation resistance is significantly higher in 5-day-old *CS* virgin females compared with virgin males (Fig 7B). Similar results were obtained with *w*^*1118*^ (Fig 7C), *Oregon-R* (S19A Fig), *Country Mill Winery* (*CMW*) flies (S19B Fig), and two isofemale lines: *Mel c2*.*2* and *Mel c2*.*3* (S19C and S19D Fig). Thus, the sexual dimorphism in starvation resistance persists in multiple genetic backgrounds. To determine whether the *bmm*-mediated regulation of triglyceride homeostasis contributes to the sex difference in starvation resistance, we examined starvation resistance in *bmm*^*1*^ mutants and *bmm*^*rev*^ controls. In line with previous studies in males, survival post-starvation was significantly longer in *bmm*^*1*^ mutants compared with *bmm*^*rev*^ controls (Fig 7D). Although starvation resistance was significantly higher in *bmm*^*1*^ mutant females compared with control females (Fig 7D), the strongly male-biased increase in starvation resistance largely eliminated the sex difference (Fig 7D), an effect we reproduced in males and females with *da-GAL4*-mediated global overexpression of two independent *UAS-bmm-RNAi* transgenes (S19E–S19H Fig). Given that *bmm* is a critical effector of the sex differences in triglyceride homeostasis, these data suggest that the sex-specific control of triglyceride homeostasis by *bmm* makes a key contribution to the sexual dimorphism in starvation resistance. Given that the enhanced starvation resistance in *bmm*^*1*^ mutant males represents a significant benefit to survival for contexts in which food is scarce, we next asked whether there were any disadvantages caused by *bmm* loss in males. Because the correct regulation of triglyceride homeostasis is essential for female fertility [23], we compared the number of offspring produced by *bmm*^*1*^ mutant males and *bmm*^*rev*^ controls. We found that *bmm*^*1*^ mutant males produced significantly fewer offspring compared with *bmm*^*rev*^ control males (S20A Fig): after being left with three females for 6 days, only five of 25 *bmm*^*1*^ mutant males produced progeny (S20A Fig). Thus, despite significant benefits in survival time after nutrient deprivation, loss of *bmm* significantly impairs normal male fertility, demonstrating a previously unrecognized role for *bmm* function in male physiology.

Because *bmm* function in several cell types and tissues plays a role in regulating sexual dimorphism in triglyceride homeostasis, we measured starvation resistance in male and female flies with cell- and tissue-specific *bmm* inhibition. In line with our finding that *bmm* inhibition in the abdominal fat body increased triglyceride storage and decreased triglyceride breakdown in both sexes, survival post-starvation in *cg>UAS-bmm-RNAi* males and females was significantly longer than *cg>+* and *+>UAS-bmm-RNAi* control flies (S21A and S21B Fig). Given that survival post-starvation was increased similarly in both sexes, the male–female difference in starvation resistance remained (S21A and S21B Fig), a finding we confirm using an additional GAL4 driver, *r4-GAL4* (S21C and S21D Fig). Therefore, *bmm* function in the abdominal fat body does not fully explain the sex difference in starvation resistance. In contrast, when we examined starvation resistance in *c587>UAS-bmm-RNAi* flies, we found that median survival post-starvation was significantly longer in *c587>UAS-bmm-RNAi* males (Fig 7E), but not females (Fig 7F), compared with *c587>+* and *+>UAS-bmm-RNAi* control flies. Importantly, we confirm that these male-specific effects on starvation resistance are specific to *bmm* using an additional *UAS-bmm-RNAi* line (S22A and S22B Fig) and by rescuing the male-specific increase in starvation resistance by *c587-GAL4*-mediated coexpression of *UAS-bmm* and *UAS-bmm-RNAi* (S22C and S22D Fig). Moreover, we observed male-biased effects on starvation resistance in *tj>UAS-bmm-RNAi* flies (S22E and S22F Fig). Because of these male-biased or male-specific effects, the sex difference in starvation resistance was reduced. Similarly, when we compared survival post-starvation in animals with neuron-specific *bmm* inhibition, we found that starvation resistance was significantly increased in *elav>UAS-bmm-RNAi* males (Fig 7G), but not females (Fig 7H), compared with control *elav>+* and *+>UAS-bmm-RNAi* control flies. This effect on starvation resistance was reproduced in flies with *elav-GAL4*-mediated expression of an additional *UAS-bmm-RNAi* line (S23A and S23B Fig) and was abolished when we used *elav-GAL4* to coexpress *UAS-bmm* and *UAS-bmm-RNAi* in neurons (S23C and S23D Fig). Furthermore, we found that *nSyb-GAL4*-mediated *bmm* inhibition in neurons strongly extended starvation resistance (S23E and S23F Fig), providing strong support for neuronal *bmm* as a regulator of starvation resistance. Because of these male-specific effects, the sex difference in survival post-starvation was reduced. Inhibition of *bmm* in other tissues had either a very modest effect or no effect on starvation resistance in either sex (S24A–S24F Fig). Together, these findings reveal a previously unrecognized difference between males and females in the physiological mechanisms that govern starvation resistance.

## Discussion

In this study, we used the fruit fly, *Drosophila melanogaster*, as a model to gain insight into the genetic and physiological mechanisms underlying sex differences in triglyceride homeostasis. We describe sexual dimorphisms in triglyceride storage and breakdown and demonstrate extensive sex-biased regulation of many genes involved in maintaining whole-body triglyceride levels. One important outcome from our study was the identification of a role for triglyceride lipase *bmm* in the regulation of sex differences in triglyceride homeostasis: loss of *bmm* largely eliminated the sex difference in triglyceride storage and abolished the sex difference in triglyceride breakdown. This represents a previously unrecognized role for *bmm* in regulating sexual dimorphism in triglyceride storage and breakdown. Another important finding was that *bmm* function in the somatic cells of the gonad and in neurons plays a role in regulating sex differences in triglyceride homeostasis. In females, *bmm* function in the abdominal fat body largely explains its regulation of whole-body triglyceride homeostasis. In contrast, in males, *bmm* acts in the fat body, the somatic cells of the gonad, and in neurons to regulate whole-body triglyceride storage and breakdown. Although we did not confirm whether the requirement for *bmm* function in the somatic cells of the gonad and in neurons affected the development of these important cell types, these findings reveal a previously unappreciated sex difference in the physiological mechanisms that govern the regulation of whole-body triglyceride levels. Moreover, we confirm that lipid droplets are present in two cell types in which knowledge of lipid droplet function is limited. Together with our data on how changes to triglyceride homeostasis affect starvation resistance and life span in each sex, our study highlights how including both sexes can accelerate the discovery of new insights into the regulation of whole-body physiology.

Our study identified many genes with sex-biased expression; however, our detailed analysis of one gene, *bmm*, the *Drosophila* homolog of mammalian *ATGL* [32,57,71], identified a previously unrecognized role for this gene in regulating sexual dimorphism in triglyceride homeostasis. *bmm* is a lipase that influences whole-body fat storage and breakdown in flies and other animals, and we found that under both normal culture conditions and post-starvation, males have higher levels of *bmm* mRNA than females. Yet what factors are responsible for this sex-specific *bmm* regulation? One possible explanation is a sex difference in food intake, as previous studies have shown that mated female flies consume more food than males [37,72]. Although our experiments use virgin males and females, a sex difference in food intake could trigger increased triglyceride storage in females by enhancing the activity of a nutrient-activated pathway, such as the insulin/insulin-like growth factor signaling (IIS) pathway [73–75]. In support of a possible role for food intake and IIS in establishing the sex difference in triglyceride storage via regulation of *bmm*, previous studies have shown that *bmm* mRNA levels are positively regulated by *forkhead box O* (*foxo*; FBgn0038197) [76,77], a transcription factor that is normally repressed by nutrient input and IIS activity [78–80]. Thus, in females, early food intake after eclosion may activate IIS pathway activity to inhibit Foxo, reducing *bmm* mRNA levels to promote triglyceride storage. In males, lower food intake would lead to less IIS signaling, increased Foxo activity, and higher levels of *bmm* mRNA to limit triglyceride storage. Indeed, a recent study in late third instar *Drosophila* larvae demonstrated increased IIS activity in females compared with males [81], and males and females show significant sex differences in gene expression in response to global IIS perturbation [82]. Future studies will therefore be important to confirm whether the sex-specific regulation of *bmm* mRNA under normal culture conditions and post-starvation occurs via IIS and Foxo. Furthermore, it will be important to test whether additional nutrient-responsive pathways contribute to the sex-specific regulation of *bmm* and the male–female difference in triglyceride storage, such as the adipokinetic hormone (AKH; FBgn0004552) pathway [68,83,84], the *sterol response element binding protein* (*SREBP*; FBgn0261283) pathway [85,86], and *spargel*/*peroxisome proliferator–activated receptor γ coactivator 1* (*srl*/*PGC-1*; FBgn0037248) pathway [87,88], as much of our knowledge of these pathways is derived from studies using either a mixed-sex population of larvae or adult male flies.

Another possible explanation for the sex differences in triglyceride homeostasis is that sex determination genes directly establish a “male” or a “female” metabolic state via regulation of triglyceride metabolism genes such as *bmm*. In support of a role for sex determination genes in metabolic regulation, previous studies have shown that at least 15 triglyceride metabolism genes are putative targets of *doublesex* (*dsx*; FBgn0000504) and *fruitless* (*fru;* FBgn0004652) [49,89], two genes that direct many [90,91], but not all [50,81,92], aspects of sexual development and behavior. Indeed, one study showed that the activity of *fru*-expressing neurons normally represses whole-body triglyceride levels in male flies [93]. Thus, *fru* and/or *dsx* may both contribute to the sex-specific regulation of triglyceride metabolism genes, a possibility that will be easily tested in future studies given the availability of viable stocks carrying isoform-specific mutations in *fru* and *dsx* [89,91,94–100]. In addition to *dsx* and *fru*, it will be important to test whether other regulators of sexual development, such as the steroid hormone ecdysone, contribute to the sex-specific regulation of triglyceride homeostasis. Previous studies have shown that changes to ecdysone signaling affect sexual development [101], and a recent study demonstrated an important role for the *ecdysone receptor* (*EcR*; FBgn0000546) in establishing the increased triglyceride storage observed in mated females [37]. Given that ecdysone levels are higher in females than in males [102,103] and the known role of steroid hormones in mammals in creating the sex difference in fat storage [7], this represents an important area for future investigations into the sexual dimorphism in triglyceride homeostasis.

A second key finding from our study was the identification of strongly sex-biased effects of whole-body *bmm* deficiency on triglyceride homeostasis. Although we confirm findings from previous studies that loss of *bmm* dramatically increases triglyceride storage and reduces triglyceride breakdown in male flies [29,32,68], we also show that loss of *bmm* had only modest effects on triglyceride storage and no effect on triglyceride breakdown in female flies. As a result of these strongly male-biased effects, the sex difference in triglyceride storage was largely eliminated, and the sex difference in triglyceride breakdown was abolished in animals with whole-body *bmm* deficiency. This reveals a previously unrecognized role for *bmm* in regulating sex differences in triglyceride homeostasis. In the future, it will be important to determine how other genes with strong sex-specific regulation contribute to male–female differences in triglyceride storage and breakdown. For example, mRNA levels of *lsd-1*/*PLIN1* were 4-fold higher in 5-day-old males compared with age-matched females. Because loss of *lsd-1*/*PLIN1* function in males leads to a significant increase in triglyceride storage compared with control males [29], it will be interesting to determine how loss of *lsd-1*/*PLIN1* affects triglyceride homeostasis in females. Another gene with strongly sex-biased expression was *hsl*, as *hsl* mRNA levels were approximately 3-fold higher in 5-day-old virgin females than in males. In a mixed-sex population of larvae, loss of *hsl* significantly increases lipid droplet size and whole-body triglyceride storage [30]; yet the adult phenotype of *hsl* mutants remains unclear. Future studies will be important to determine how this highly conserved lipase affects triglyceride storage and breakdown in each sex. Moreover, because our data show that loss of *bmm* in additional cell types, such as the somatic cells of the gonad and neurons, influences triglyceride storage and breakdown, future studies on *lsd-1*/*PLIN1*, *hsl*, and other genes will need to define whether there is a general requirement for triglyceride metabolism genes in these important cell types in the control of whole-body triglyceride homeostasis.

In most animals, the majority of whole-body triglycerides are contained within specialized organs dedicated to fat storage, such as the mammalian adipose tissue and insect fat body [3,104]. Interestingly, we show that some of the male-biased effects of *bmm* on triglyceride homeostasis were due to cell types in addition to the fat body, where *bmm* function has been well-described. Although we cannot rule out a role for the *Drosophila* head fat body in mediating some of *bmm*’s sex-specific effects on triglyceride storage and breakdown, as our fat body drivers were specific to the abdominal fat body (S3 Table), we found that *bmm* function in the somatic cells of the male gonad and in male neurons explained at least some of the male-biased effects of whole-body *bmm* loss on triglyceride homeostasis. Given the growing recognition that lipid droplets and triglyceride homeostasis in many cell types and tissues contribute to the normal regulation of whole-body development and physiology [25,66,105–109], our study highlights the importance of exploring how the control of triglyceride metabolism in one cell type impacts whole-body triglyceride homeostasis. For example, how does *bmm* function in the somatic cells of the gonad, a cell type not known to be a major site of triglyceride storage, affect whole-body triglyceride storage and breakdown? In the mammalian gonad, lipid droplets have been detected in both the Leydig and Sertoli cells [110–113]. An important function of the mammalian testis lipid droplets is the storage of cholesterol ester, which can be broken down to release free cholesterol for biosynthesis of the steroid hormone testosterone [114]. In flies, although the precise lipid composition of the testis lipid droplets is unknown, the steroid hormone ecdysone has been detected in the testis [102,115]. Thus, if lipid droplets in the *Drosophila* testis contribute to steroid hormone production in males, the ectopic lipid droplets in animals lacking *bmm* in the somatic cells of the gonad may alter neutral lipid metabolism and affect ecdysone production in male flies. In support of a model in which ectopic ecdysone production influences whole-body triglyceride storage, a previous study showed that ecdysone feeding in adult males was sufficient to enhance fat storage [37]. In the future, it will be important to directly test this model by examining changes to ecdysone titers and ecdysone signaling in males lacking *bmm* in the somatic cells of the gonad. Moreover, given that previous studies have shown important effects of the germline and gonad on whole-body gene expression [103,116] and phenotypes such as aging and immunity [117–120], it will be interesting to examine other aspects of development, physiology, and life span in males lacking *bmm* function in the gonad. Ultimately, a better mechanistic understanding of how changes to *bmm* function in the somatic cells of the gonad affects whole-body fat storage and breakdown will provide insight into how *bmm* function in diverse cell types might impact other aspects of development and physiology and suggest lines of inquiry for studies on *ATGL* in other models.

In addition to the somatic cells of the gonad, we also identified an important role for *bmm* function in *Drosophila* neurons in the regulation of triglyceride breakdown. In many animals, correct regulation of lipid metabolism in neurons is important for membrane synthesis and remodeling and in mediating signaling events within the neuron [62–64]. Although previous studies have detected lipid droplets in cultured neurons and brain sections [121–124] in *Drosophila* larval motor neuron axons [125] and have shown that neuronal dysfunction is associated with abnormal lipid droplet accumulation [126–128], more studies are needed to improve knowledge of the normal physiological roles of lipid droplets in neurons [127–129]. For example, to understand how *bmm* function in neurons promotes whole-body triglyceride breakdown, it will be important to identify which subsets of neurons require *bmm* function to control triglyceride breakdown. One obvious group of neurons are the AKH-producing cells in *Drosophila* [83,84]. Under normal culture conditions, ablation of AKH-producing neurons and loss of AKH peptide have no effect on development [83,84,130–132]; however, AKH neurons and AKH receptor–mediated signaling are required for starvation-mediated triglyceride breakdown [68,131]. This fits with our data that *bmm* function in neurons only affects triglyceride breakdown and suggests a model in which a male-specific increase in *bmm* within AKH neurons post-starvation triggers AKH secretion and rapid lipolysis. In the future, more studies will be needed to confirm whether AKH secretion is responsible for the sex difference in whole-body triglyceride depletion post-starvation. Moreover, because manipulation of insulin-producing cells (IPCs) in the CNS [133], *fru*-expressing neurons in the mushroom bodies [93,134], octopamine- and tyramine-producing neurons [135,136], Taotie neurons [137], short neuropeptide F (sNPF)-producing neurons [138], and central clock pigment dispersing factor (PDF)-positive neurons [139] affects whole-body triglyceride levels, future studies will need to examine whether *bmm* function in any of these neurons contributes to the sex difference in triglyceride breakdown.

Once the neuroanatomical focus of *bmm*’s effects on triglyceride breakdown has been identified, it will be important to investigate how loss of *bmm* affects neuronal development and/or function. For example, whole-body deficiency for *bmm*’s murine homolog *ATGL* causes profound changes to the composition of triglyceride-associated fatty acids in the mouse brain [140]. Loss of *bmm* in neurons may therefore affect fatty acid–mediated signaling in the *Drosophila* brain. Although the changes to fatty acid composition that accompany *bmm* loss have not been characterized in *Drosophila*, one study showed that the *Drosophila* homolog of nuclear hormone receptor hepatocyte nuclear factor 4 (HNF4) is positively regulated by *bmm* activity in the larval fat body [141]. Thus, HNF4 is a promising candidate to mediate the effects of *bmm* loss on neuronal development and/or function. Another possibility includes changes to the activity of SREBP because SREBP processing is normally blocked by the fatty acid palmitate in *Drosophila* [85]. Moreover, given that several studies have identified clear links between lipid droplets and reactive oxygen species (ROS) signaling in the *Drosophila* brain [66,108,109], *bmm*-mediated changes to ROS signaling in neurons will also need to be examined during starvation in both sexes. Overall, although the mechanism underlying the effect of *bmm* function in neurons on whole-body triglyceride breakdown is unknown, our identification of lipid droplets in neurons under normal culture conditions suggests *Drosophila* is a useful model to examine how lipid droplets contribute to the normal function of this important cell type.

In conclusion, our studies identify a role for triglyceride lipase *bmm* in the regulation of sex differences in *Drosophila* triglyceride storage and breakdown. We show that *bmm* function is required in the somatic cells of the gonad and in neurons to maintain whole-body triglyceride homeostasis, provide the first evidence that *bmm* is sex-specifically regulated, and demonstrate the role of this regulation to the male–female difference in triglyceride homeostasis and starvation resistance. Given that the correct regulation of triglyceride homeostasis has also been linked with the regulation of sleep, fertility, reproduction, and feeding [23,36,37,43,72,142–148], our studies raise the possibility that the male–female differences previously noted in at least some of these complex traits may be associated with the sex difference in *Drosophila* triglyceride homeostasis. Looking beyond *Drosophila*, it will be interesting to determine whether *bmm* also contributes to sex differences in triglyceride storage and breakdown in other animals because *bmm* homologs are found in many species [32,57,58,71]. Furthermore, given the sex-biased risk of developing diseases associated with abnormal triglyceride metabolism (e.g., cardiovascular disease, nonalcoholic fatty liver disease) [149–151], future studies on the cell- and tissue-specific regulation of triglyceride metabolism in both sexes will be essential to provide insight into the mechanisms that contribute to disease onset and progression in each sex.

## Materials and methods

### Fly husbandry

Fly stocks were reared at 25°C in a 12:12 light:dark cycle. All transgenic flies were backcrossed into a *w*^*1118*^ genetic background for a minimum of five generations. For all experiments, larvae were raised at a density of 50 larvae per 10 ml of yeast–sugar–cornmeal media [152] (see recipe in S1 Materials and Methods). Pupae were sexed either by gonad size or by the presence of sex combs and placed onto filter paper to complete pupal development. Flies eclosed into single-sex vials at a density of 20 animals per vial, and adult weight was measured by weighing groups of 10 flies in preweighed, 1.5-ml microcentrifuge tubes. Unless otherwise indicated, all assays were performed on 5- to 7-day-old adult flies. For metabolic assays, five flies were collected immediately prior to starvation and afterward at 12-hour intervals post-starvation. Flies were snap frozen on dry ice and stored at –80°C. Each biological replicate represents five flies collected into a 1.5-ml microcentrifuge tube, and each experiment includes four biological replicates for each sex and genotype. All experiments were repeated a minimum of two times for a total number of eight biological replicates per sex and per genotype.

### Fly strains

The following fly strains from the Bloomington *Drosophila* Stock Center were used in this study: *CS* (#64349), *w*^*1118*^ (#3605), *Oregon-R*, *y*^*1*^_,_*v*^*1*^*;UAS-bmm-RNAi* (#25926), *y*^*1*^_,_*v*^*1*^*;attP40* (#36304), *w*^*1118*^*;UAS-nGFP* (#4775); *UAS-mCD8*::*GFP* (#5130). The following fly strains from the Vienna *Drosophila* Resource Center were used in this study: *UAS-bmm-RNAi* (#37880), *UAS-bmm-RNAi* (#37877). We obtained the *bmm*^*1*^ mutants and *bmm*^*rev*^ control strain as a kind gift from R. Kühnlein [32]; *CMW* flies, *Mel c2*.*2*, and *Mel c2*.*3* (wild-caught isofemale lines) as a kind gift from I. Dworkin; and *UAS-LD-GFP* flies from M. Welte. We used the following stocks for tissue-specific overexpression of the *UAS-bmm*-*RNAi* transgene: *da-GAL4* (ubiquitous), *cg-GAL4* (abdominal fat body), *r4-GAL4* (abdominal fat body) *elav-GAL4* (postmitotic neurons), *nSyb-GAL4* (neurons), *repo-GAL4* (glia), *Mex-GAL4* (intestinal enterocytes), *c587-GAL4* (somatic cells of the gonad), *tj-GAL4* (somatic cells of the gonad), and *dMef2-GAL4* (muscle cells).

### Starvation resistance

The 5- to 7-day-old virgin males and females raised on normal media were transferred to vials containing 2 ml of starvation media (0.7% agar in 1× PBS). Dead flies were counted at 12-hour intervals post-starvation. Each experiment used >200 flies per sex and per genotype, and was performed at least twice (total n>400 flies per sex and per genotype).

### Male fertility assay

The 5-day-old virgin males were paired with three 5-day-old virgin *w*^*1118*^ females. On alternate days after mating, the group of four flies was transferred to fresh food vials. The old vials were kept, and male fertility was quantified by counting the number of pupae.

### Triglyceride assay

Flies were collected as described above and homogenized using 100 μl of glass beads (Sigma 11079110) in 200 μl of buffer (0.1% Tween in 1× PBS) at 8.0 m/s for 5 seconds (OMNI International Bead Ruptor 24). Triglyceride concentration was measured using a coupled colorimetric assay as previously described [153]. For a detailed description of methods, refer to S1 Materials and Methods.

### Protein assay

Flies were collected as described above and homogenized using 100 μl of glass beads (Sigma 11079110) in 500 μl of 1× PBS at 8.0 m/s for 5 seconds (OMNI International Bead Ruptor 24). Protein concentration was measured as previously described [154]. For a detailed description of methods, refer to S1 Materials and Methods.

### Glucose and glycogen assay

Flies were collected as described above and homogenized using 100 μl of glass beads (Sigma 11079110) in 500 μl of 1× PBS at 8.0 m/s for 5 seconds (OMNI International Bead Ruptor 24). Glucose or glycogen concentration was measured as previously described [153]. For a detailed description of methods, refer to S1 Materials and Methods.

### RNA extraction and cDNA synthesis

Each biological replicate represents 10 flies that were frozen in a 1.5-ml microcentrifuge tube on dry ice and stored at −80°C until processing. Each experiment contained four biological replicates per sex and per genotype, and each experiment was repeated at least twice. Total RNA was extracted as previously described [81,154]. Genomic DNA was eliminated and cDNA was synthesized using the QuantiTect Reverse Transcription Kit (Qiagen) according to the manufacturer’s instructions. For a detailed description of methods, refer to S1 Material and Methods.

### qPCR

qPCR was performed in a 15-μL reaction volume containing 2 μL of diluted cDNA and final concentrations of 0.6 U of Platinum or 0.3 U of recombinant Taq DNA Polymerase (ThermoFisher Scientific), 0.1× SYBR Green I Nucleic Acid Gel Stain (ThermoFisher Scientific), 0.3 μM of specific primer pairs (Integrated DNA Technologies, Eurofin Genomics, ThermoFisher Scientific), 1× PCR buffer (ThermoFisher Scientific), 125 μM dNTP mix (FroggaBio), and 1.5 mM MgCl_2_ (ThermoFisher Scientific). qPCR was carried out in a CFX384 Touch Real-Time PCR Detection System (BioRad). Thermocycler conditions were as follows: initial denaturation for 3 minutes at 95°C and then 40 cycles of denaturation for 30 seconds at 95°C, annealing for 30 seconds at 60°C, and extension for 45 seconds at 72°C. Data were normalized to the average fold change of β-tubulin. For a full primer list, refer to S1 Material and Methods.

### Sex difference in gene expression

To show the sex bias in gene expression, fold change was calculated as 2^(absolute value of ΔCT). Sex-biased gene expression graph and heat maps were generated in RStudio (version 1.0.153) using the code below.

### Radar plots

The average fold change in *bmm* mRNA (or other mRNA) in males and females between 0 and 24 hours post-starvation was generated by 2^ΔCT. The ΔCT is the difference between the average CT value for each sex and time point compared with the average CT value of females at 0 hours post-starvation.

### Metabolic rate measurements

Virgin males and females were sexed post-eclosion and aged for 5 days at 25°C on a 12:12 light:dark cycle. Two hours after lights on, single flies were lightly anaesthetized with CO_2_, and wet mass per individual was recorded to the nearest microgram. Individual flies were placed into 5-ml syringes that were modified to allow for gas flow along with a small cap filled with standard food (0.88% agar, 8.33% torula yeast, 10% cornmeal, 0.33% Tegosept w/v and 4.66% molasses, 1.66% ethanol (95% v/v), 0.66% propionic acid v/v dH_2_O) or with starvation medium (0.7% agarose in 1× PBS). At least 20 biological replicates per sex per treatment were measured for metabolic rate in a randomly assigned order. We used stop-flow respirometry to estimate metabolic rate as the volume of CO_2_ (VCO_2_) produced and the volume of O_2_ (VO_2_) consumed, which allowed for calculation of the RQ (RQ = VCO_2_/VO_2_), as previously described [155] (Sable Systems International, Las Vegas, NV). Any flies that died during the observation period were excluded from our analysis. All respirometry data were analyzed using the Expedata software package (Sable Systems) as previously described [155]. To test for differences in metabolic rates across treatments within each sex and time point, we estimated the scaling relationship between mass and VCO_2_ and mass and VO_2_ using Type II Model regression in the smatR package in R [156]. If the slopes were significantly different, then no further tests were performed; however, if the slopes were the same, we tested for effects of treatment on the elevation (i.e., on mass-specific metabolic rate) and as a shift along the x-axis (i.e., a difference in mass). In order to generate mass-corrected metabolic rates, we took residuals of these regressions and added back the grand mean for each group [155]. We also tested for differences in VCO_2_, VO_2_, and RQ between treatments within each sex and window of time using Tukey HSD tests. We also tested for differences in mass-corrected VCO_2_ and VO_2_. For a detailed description of methods, refer to S1 Material and Methods.

### Lipid droplet visualization and quantification

Dissected testis from newly eclosed males and CNSs dissected from 5- to 7-day-old adult virgin males and females of the indicated genotypes were fixed for 30 minutes in 4% paraformaldehyde at room temperature. After three 10-minute washes in 1× PBS, the dissected tissues were incubated in 1× PBS with either a 1:50 dilution of HCS LipidTOX (Invitrogen, H34476) or BODIPY 493/503 (ThermoFisher, D3922) for 30 minutes. To visualize nuclei in the testis, Hoechst 33342 (ThermoFisher, 62249) was included with LipidTOX/BODIPY at a dilution of 1:1,000. Images were acquired on a Leica SP5 confocal microscope. Neutral lipid levels within each brain were measured as the sum of fluorescence using ImageJ.

### Statistics

qPCR data were analyzed using one-way ANOVA paired with Tukey’s multiple comparisons test on software package Prism 6 (GraphPad). For all statistical analyses, differences were considered significant if the *p*-value was <0.05. All other data were analyzed using RStudio with the code described below. The lowest *p*-value provided by R is 2 × 10^−16^; therefore, many statistical tests show the same *p*-value. Error bars on graphs representing change in whole-body triglyceride level were calculated using the coefficient of error (COE—standard error of the mean as a percentage of the mean) as described in [157].

Log-rank test (R package “survminer”):

pairwise_survdiff(Surv(time, event) ~ genotype, data, p.adjust.method = "bonferroni")

surv_median(curve)

summary(data)

One-way ANOVA:

Results ← aov(value ~ genotype, data)

TukeyHSD(Results, conf.level = 0.95)

Student *t* test:

t.test(value ~ genotype, data, var.equal = TRUE)

Two-way ANOVA:

aov(percentageTG ~ genotype + sex, data)

### Graphs

All graphs were prepared in R using packages “ggplot2,” “gtable,” “grid,” “survminer,” “survival,” “fmsb,” and “pheatmap” using the code described below.

Survival curve:

library (“survminer”)

library (“survival”)

curve <- survfit(Surv(time, event) ~ genotype, data)

max <- curve$time[which.max(curve$time)]

graph <- ggsurvplot(curve, size = 3, data, fun = "pct", palette = c(" "), surv.geom = geom_line, conf.int = TRUE, conf.int.style = c("step"), xlim = c(0, max), break.time.by = 12) + labs(x = NULL, y = NULL) + theme_survminer(legend = "non", font.tickslab = c(0))

Box and whisker plot:

library (“ggplot2”)

library (“gtable”)

library (“grid”)

roundUP <- ceiling(data$percentageTG[which.max(data$percentageTG)])

ggplot(data = data, aes(x = genotype, y = percentageTG, fill = genotype)) + stat_boxplot(geom = "errorbar", width = 0.5) + geom_boxplot() + scale_fill_manual (values = colours) + geom_dotplot(binaxis = 'y', stackdir = 'center', position = "dodge", pch = 21, col = "black", bg = "white", dotsize = 0.5) + facet_grid(. ~ time) + theme(legend.position = "none", panel.grid.major = element_blank(),panel.grid.minor = element_blank(), panel.background = element_blank(), axis.line = element_line(colour = "black", size = 1), axis.ticks.length = unit(0.25, "cm"), axis.ticks.y = element_line(size = 1), axis.ticks.x = element_blank(), axis.title = element_blank(), axis.text = element_blank()) + scale_y_continuous(breaks = seq(0, roundUP, by = 1), limits = c (0, roundUP)) + theme(strip.background = element_blank(), strip.text = element_blank())

Sex-biased gene expression graph:

library (“ggplot2”)

ggplot (data, aes(x = gene_name, y = average, fill = sex_bias)) + geom_errorbar(aes(ymin = average-data$sem, ymax = average+data$sem), width = 0.5) + geom_bar (position = position_dodge(), stat = "identity") + theme(axis.text.x = element_text(angle = 45, hjust = 1)) + scale_y_continuous (breaks = seq(-20, 60, by = 20), limits = c(-20, 60)) + theme(legend.position = "none", panel.grid.major = element_blank(), panel.grid.minor = element_blank(), panel.background = element_blank(), axis.line = element_line(colour = "black", size = 0.5), axis.ticks.length = unit(0.25, "cm"), axis.ticks.y = element_line(size = 0.5), axis.title = element_blank(), axis.text.y = element_blank()) + scale_fill_manual(values = c("#f39e1f", "#3a3b95", "#cdcdcd"))

Gene expression heat map:

library(“pheatmap”)

pheatmap(measurements.avg.stat[,c(2:9)], cluster_rows = F, cluster_cols = F, color = colour, border_color = “#FFFFFF”, width = 10,height = 8,filename = “heatmap_expression_F.pdf”, na_col = “#DDDDDD”, breaks = seq(0,5,0.001), annotation_row = annotation_row, cellwidth = 15, cellheight = 15)

Radar plot:

library(“fmsb”)

radarchart (data[2:7], maxmin = TRUE, axistype = 0, seg = 9, caxislabels = seq(0, 9, 1), pty = 16, pcol = "black", pfcol = fill, plty = 1, cglty = 1, cglcol = "#bebebe", vlabels = NA)

## Supporting information

S1 Fig Sex differences in triglyceride storage and breakdown are not due to male and female gonads. (A) Ovary triglyceride levels were not significantly different between fed virgin *w*^*1118*^ females and starved virgin *w*^*1118*^ females at all time points STV (*p* = 0.56, 0.44, 0.55, respectively; one-way ANOVA followed by Tukey HSD test). (B) The amount of triglyceride contained in the testes of 5-day-old virgin *w*^*1118*^ males is below the limit of detection for the coupled colorimetric assay; therefore, statistics could not be performed. (C) Triglyceride levels in 5-day-old virgin *w*^*1118*^ female carcasses devoid of ovaries were significantly higher than in age-matched male carcasses devoid of testes (*p* = 0.022; Student *t* test). (D) Larval fat cells in newly eclosed *CS* virgin females and males showed no significant change between 0 and 12 hours post-eclosion (*p* = 0.53 and 0.43 for females and males, respectively; one-way ANOVA followed by Tukey HSD test), but there was a significant decrease in the larval fat cell number in both sexes between 12 and 24 hours post-eclosion (*p* = 0.0 and 0.0 for females and males, respectively; one-way ANOVA followed by Tukey HSD test). (E) The number of larval fat cells significantly decreased in *w*^*1118*^ virgin females and males between 0 and 12 hours post-eclosion (*p* = 0.0071 and 1.0 × 10^−7^ for females and males, respectively; one-way ANOVA followed by Tukey HSD test) and decreased further in females but not males between 12 and 24 hours post-eclosion (*p* = 0.011 and 0.8 for females and males, respectively; one-way ANOVA followed by Tukey HSD test). (F) After 24 hours of starvation, triglyceride levels in virgin *CS* females remain at 77% of the triglyceride level in a fed virgin female (*p =* 0.034; one-way ANOVA followed by Tukey HSD test), whereas virgin *CS* males have only 4% of the triglyceride level in a fed male remaining (*p* = 6 × 10^−7^; one-way ANOVA followed by Tukey HSD test). (G) In 5-day-old virgin female *w*^*1118*^ carcasses devoid of ovaries, there was no significant decrease in triglyceride levels between 0 and 12 hours STV, whereas there was a significant reduction in triglyceride levels in age-matched *w*^*1118*^ virgin male carcasses devoid of testes (*p* = 0.15 and 0.013, respectively; one-way ANOVA followed by Tukey HSD test). Between 12 and 24 hours STV, there was a male-biased decrease in triglyceride levels in male and female carcasses devoid of gonads (*p* = 0.00065 and 0.0051, respectively; one-way ANOVA followed by Tukey HSD test). Asterisks indicate a significant difference between two sexes, two genotypes, or two time points (*p < 0.05, **p < 0.01, ***p < 0.001). Error bars on graphs depicting percent body fat represent SEM; error bars on graphs depicting the change in percent body fat represent COE. See S1 Table for a list of all multiple comparisons and *p*-values; quantitative measurements underlying all graphs are available in S1 Data. COE, coefficient of error; *CS*, *Canton-S*; HSD, honest significant difference; ns, no significant difference between two sexes, two genotypes, or time points; STV, post-starvation; *w*, *white*.
(TIF)

S2 Fig Sex difference in metabolic rate under normal culture and starvation conditions. (A) Non-mass-corrected CO_2_ production was significantly higher in *Oregon-R* fed females compared with fed males for the majority of the intervals during the 24-hour observation period (*p* = 0.067, 0.0031, 2.4 × 10^−4^, 4.5 × 10^−4^, 1.4 × 10^−5^, 1.7 × 10^−7^, respectively; Student *t* test at each time interval). (B) Non-mass-corrected O_2_ consumption was significantly higher in fed females compared with fed males at all intervals during the observation period (*p* = 1.5 × 10^−5^, 1.6 × 10^−5^, 6.0 × 10^−6^, 5.8 × 10^−5^, 1.8 × 10^−5^, 3.8 × 10^−5^, respectively; Student *t* test at each time interval). (C) Non-mass-corrected CO_2_ production was significantly higher in starved females at every interval post-starvation from 4 hours onward (*p* = 0.44, 1.3 × 10^−5^, 5.9 × 10^−13^, 2.4 × 10^−9^, 1.9 × 10^−7^, 1.5 × 10^−4^, respectively; Student *t* test at each time interval). (D) Non-mass-corrected O_2_ consumption was significantly higher in starved females compared with starved males at all time intervals post-starvation (*p* = 6.0 × 10^−12^, 1.1 × 10^−15^, 1.2 × 10^−14^, 4.3 × 10^−10^, 1.7 × 10^−8^, 2.5 × 10^−5^, respectively; Student *t* test at each time interval). For indirect calorimetry measurements, the *p*-values are listed in the following order: difference between the sexes at 2–4 hours, 4–8 hours, 8–12 hours, 12–16 hours, 16–20 hours, and 20–22 hours. Asterisks indicate a significant difference between two sexes, two genotypes, or two time points (*p < 0.05, **p < 0.01, ***p < 0.001). Error bars on graphs represent SEM. Quantitative measurements underlying all graphs are available in S2 Data. ns, no significant difference between two sexes, two genotypes, or time points.
(TIF)

S3 Fig Starvation changes metabolic function in both females and males. (A) Non-mass-corrected CO_2_ production was significantly higher in *Oregon-R* fed females compared with starved females for most intervals post-starvation during the observation period (*p* = 0.20, 0.0024, 4.9 × 10^−5^, 5.2 × 10^−5^, 1.4 × 10^−6^, 1.6 × 10^−9^, respectively; Student *t* test at each time interval). (B) Non-mass-corrected CO_2_ production was significantly higher in *Oregon-R* fed males compared with starved males for most intervals post-starvation during the observation period (*p* = 0.99, 6.1 × 10^−5^, 4.7 × 10^−10^, 2.2 × 10^−9^, 1.3 × 10^−5^, 4.1 × 10^−9^, respectively; Student *t* test at each time interval). (C) Non-mass-corrected O_2_ consumption was significantly higher in fed females compared with starved females for most intervals post-starvation during the observation period (*p* = 0.072, 0.0080, 0.0013, 8.1 × 10^−4^, 7.7 × 10^−6^, 6.6 × 10^−8^, respectively; Student *t* test at each time interval). (D) Non-mass-corrected O_2_ consumption was significantly higher in fed males compared with starved males at all intervals post-starvation (*p* = 1.6 × 10^−4^, 9.6 × 10^−6^, 1.5 × 10^−7^, 3.6 × 10^−6^, 9.3 × 10^−6^, 4.8 × 10^−6^, respectively; Student *t* test at each time interval). (E) Mass-corrected CO_2_ production was significantly higher in fed females compared with starved females for most intervals post-starvation during the observation period (*p* = 0.55, 0.0026, 4.9 × 10^−5^, 0.0016, 1.3 × 10^−4^, 8.1 × 10^−9^, respectively; Student *t* test at each time interval). (F) Mass-corrected CO_2_ production was significantly higher in fed males compared with starved males for most intervals post-starvation during the observation period (*p* = 0.59, 4.4 × 10^−4^, 7.5 × 10^−10^, 2.0 × 10^−9^, 7.0 × 10^−7^, 3.0 × 10^−10^, respectively; Student *t* test at each time interval). (G) Mass-corrected O_2_ consumption was significantly higher in fed females compared with starved females for most intervals post-starvation during the observation period (*p* = 0.053, 0.014, 0.0098, 0.063, 7.6 × 10^−4^, 6.2 × 10^−6^, respectively; Student *t* test at each time interval). (H) Mass-corrected O_2_ consumption was significantly higher in fed males compared with starved males at all intervals post-starvation (*p* = 5.0 × 10^−5^, 2.6 × 10^−6^, 4.7 × 10^−8^, 1.0 × 10^−6^, 8.3 × 10^−7^, 1.1 × 10^−6^, respectively; Student *t* test at each time interval). For indirect calorimetry measurements, the *p*-values are listed in the following order: difference between the treatments at 2–4 hours, 4–8 hours, 8–12 hours, 12–16 hours, 16–20 hours, and 20–22 hours. Asterisks indicate a significant difference between two sexes, two genotypes, or two time points (*p < 0.05, **p < 0.01, ***p < 0.001). Error bars on graphs represent SEM. Quantitative measurements underlying all graphs are available in S2 Data. ns, no significant difference between two sexes, two genotypes, or time points.
(TIF)

S4 Fig Sexual dimorphism in macronutrient usage under starvation conditions. (A) In fed *Oregon-R* females and males, we observed no significant differences in the RQ throughout most of the observation period, with the exception of the 4- to 8-hour interval (*p* = 0.17, 0.031, 0.13, 0.43, 0.58, 0.15, respectively; Student *t* test at each time interval). (B) In starved *Oregon-R* females and males, starved males have a significantly higher RQ at all time intervals post-starvation (*p* = 0.0012, 0.0013, 7.7 × 10^−4^, 6.6 × 10^−5^, 0.0013, 0.0032, respectively; Student *t* test at each time interval). For indirect calorimetry measurements, the *p*-values are listed in the following order: difference between the sexes at 2–4 hours, 4–8 hours, 8–12 hours, 12–16 hours, 16–20 hours, and 20–22 hours. Asterisks indicate a significant difference between two sexes, two genotypes, or two time points (*p < 0.05, **p < 0.01, ***p < 0.001). Error bars on graphs represent SEM. Quantitative measurements underlying all graphs are available in S2 Data. ns, no significant difference between two sexes, two genotypes, or time points; RQ, respiratory quotient.
(TIF)

S5 Fig Post-starvation macronutrient breakdown in males and females. (A) Whole-body protein levels were not significantly different between 5-day-old virgin *w*^*1118*^ males and females at any time point STV (*p* = 0.16, 0.19, 0.37, respectively; Student *t* test at each time point). (B) Whole-body glucose levels were not significantly different between the sexes at 0 and 12 hours STV but were significantly higher in females compared with males by 24 hours STV (*p* = 0.87, 0.48, 0.034, respectively; Student *t* test at each time point). (C) Whole-body glycogen levels were not significantly different between the sexes at 0 or 12 hours STV but were significantly higher in females compared with males by 24 hours STV (*p* = 0.86, 0.063, 0.033, respectively; Student *t* test at each time point). The *p*-values are listed in the following order: difference between females and males at 0 hours, 12 hours, and 24 hours STV. Asterisks indicate a significant difference between two sexes, two genotypes, or two time points (*p < 0.05; **p < 0.01, ***p < 0.001). Error bars on graphs represent SEM. Quantitative measurements underlying all graphs are available in S1 Data. ns, no significant difference between two sexes, two genotypes, or time points; STV, post-starvation; *w*, *white*.
(TIF)

S6 Fig Sex-specific expression of a selection of triglyceride metabolism genes normalized to an additional housekeeping gene. (A) In normal culture conditions, *Agpat4* is female-biased, *mdy* is male-biased, and *PAPLA1* is not sex biasedly expressed when normalized to *β-tubulin* (*p* = <0.0001, 0.0079, and 0.25, respectively; Student *t* test for each gene). (B) In normal culture conditions, *Agpat4* is female biased and *mdy* and *PAPLA1* are male biased when normalized to both *β-tubulin* and *β-cop* (*p* = <0.0001, 0.0015, and 0.048, respectively; Student *t* test for each gene). Asterisks indicate a significant difference between two sexes, two genotypes, or two time points (*p < 0.05; **p < 0.01, ***p < 0.001). Error bars on graphs represent SEM. Quantitative measurements underlying all graphs are available in S3 Data. *β-cop*, *Coat Protein (coatomer) β*; *Agpat*, 1-acylglycerol-3-phosphate *O*-acyltransferase; ns, no significant difference between two sexes, two genotypes, or time points; *mdy*, *midway*; *PAPLA1*, *phosphatidic acid phospholipase A1*.
(TIF)

S7 Fig Gene expression during starvation normalized to an additional housekeeping gene. (A–C) In starvation conditions, *Agpat4*, *mdy*, and *PAPLA1* female gene expression is significantly increased at 24 hours post-starvation when normalized to *β-tubulin* (*p* = <0.0001, <0.0001, and <0.0001, respectively at 24 hours post-starvation; one-way ANOVA followed by Tukey HSD test for each gene). (D–F) In starvation conditions, *Agpat4* and *PAPLA1* female gene expression is not significantly increased at 24 hours post-starvation, whereas *mdy* is significantly increased at 24 hours post-starvation when normalized to *β-tubulin* and *β-cop* (*p* = >0.05, >0.05, and <0.01 respectively at 24 hours post-starvation; one-way ANOVA followed by Tukey HSD test for each gene). (G–I) In starvation conditions, *Agpat4* and *PAPLA1* male gene expression is not significantly increased at 24 hours post-starvation, whereas *mdy* is significantly increased at 24 hours post-starvation when normalized to *β-tubulin* (*p* = >0.05, >0.05, and <0.001, respectively, at 24 hours post-starvation; one-way ANOVA followed by Tukey HSD test for each gene). (J–L) In starvation conditions, *Agpat4* and *PAPLA1* male gene expression is not significantly increased at 24 hours post-starvation, whereas *mdy* is significantly increased at 24 hours post-starvation when normalized to *β-tubulin* and *β-cop* (*p* = >0.05, >0.05, and <0.05, respectively, at 24 hours post-starvation; one-way ANOVA followed by Tukey HSD test for each gene). Asterisks indicate a significant difference between two sexes, two genotypes, or two time points (*p < 0.05, **p < 0.01, ***p < 0.001). Error bars on graphs represent SEM. See S1 Table for list of all comparisons and *p*-values; quantitative measurements underlying all graphs are available in S3 Data. *β-cop*, *Coat Protein (coatomer) β*; *Agpat*, 1-acylglycerol-3-phosphate *O*-acyltransferase; HSD, honest significant difference; *mdy*, *midway*; ns, no significant difference between two sexes, two genotypes, or time points; *PAPLA1*, *phosphatidic acid phospholipase A1*.
(TIF)

S8 Fig Sex differences in gene expression are stable throughout the post-starvation period. Radar plots demonstrating gene expression in males and females throughout the starvation period for representative genes. (A, B) mRNA levels of two genes with male-biased expression throughout the starvation period in both males and females (*Lpin*, *CG1941*). (C, D) mRNA levels of two genes with female-biased expression throughout the starvation period in both males and females (*hsl*, *wun2*). Sex-biased expression of these genes remains consistent throughout the starvation period. See S1 Table for list of all comparisons and *p*-values; quantitative measurements underlying all graphs are available in S3 Data. *hsl*, *hormone-sensitive lipase*; *Lpin*, *Lipin*; STV, post-starvation; *wun2*, *wunen-2*.
(TIF)

S9 Fig Loss of *bmm* abolishes the sex difference in triglyceride storage in carcasses devoid of gonads. (A) Triglyceride levels in 5-day-old *bmm*^*1*^ mutant females fed a high fat diet (HFD) were significantly higher than in *bmm*^*1*^ mutant females fed standard fly food (*p* = 3.9 × 10^−6^; Student *t* test). (B) Triglyceride storage was significantly higher in 5-day-old virgin *bmm*^*rev*^ female carcasses lacking ovaries compared with age-matched *bmm*^*rev*^ males, whereas there was no significant difference in whole-body triglyceride levels in 5-day-old *bmm*^*1*^ mutant virgin female carcasses devoid of ovaries compared with age-matched *bmm*^*1*^ mutant virgin males (*p* = 0.00013 in *bmm*^*rev*^ animals, 0.12 in *bmm*^*1*^ mutants; one-way ANOVA followed by Tukey HSD test). Asterisks indicate a significant difference between two sexes, two genotypes, or two time points (*p < 0.05, **p < 0.01, ***p < 0.001). Error bars on graphs represent SEM. See S1 Table for list of all multiple comparisons and *p*-values; quantitative measurements underlying all graphs are available in S1 Data. *bmm*, *brummer*; F, female; HFD, high-fat diet; HSD, honest significant difference; M, male; ns, no significant difference between two sexes, two genotypes, or time points.
(TIF)

S10 Fig Male-biased effects of ubiquitous RNAi-mediated *bmm* inhibition reduces sex differences in triglyceride storage and breakdown. (A) In 5-day-old virgin *da>UAS-bmm-RNAi* males, triglyceride levels were significantly higher than in *da>+* or *+>UAS-bmm-RNAi* control males (*p* = 0.012 and 6.0 × 10^−7^, respectively; one-way ANOVA followed by Tukey HSD test). (B) Triglyceride levels in 5-day-old virgin *da>UAS-bmm-RNAi* females were not significantly different from *da>+* or *+>UAS-bmm-RNAi* control females (*p* = 0.98 and 0.16, respectively; one-way ANOVA followed by Tukey HSD test). (C) The male-biased effects of *da>UAS-bmm-RNAi* on triglyceride storage reduced the sexual dimorphism in triglyceride storage compared with *da>+* or +>*UAS-bmm-RNAi* controls. (D) In 5-day-old virgin *da>UAS-bmm-RNAi#2* (BDSC #25926) males, triglyceride levels were significantly higher than in *da>+* or *+>UAS-bmm-RNAi#2* control males (*p* = 0.0 and 0.0, respectively; one-way ANOVA followed by Tukey HSD test). (E) Triglyceride levels in 5-day-old virgin *da>UAS-bmm-RNAi#2* (BDSC #25926) females were not significantly different from *da>+* or *+>UAS-bmm-RNAi#2* control females (*p* = 5.8 × 10^−5^ and 0.14, respectively; one-way ANOVA followed by Tukey HSD test). (F) The male-biased effects of *da>UAS-bmm-RNAi#2* (BDSC #25926) on triglyceride storage reduced the sexual dimorphism in triglyceride storage compared with *da>+* or *+>UAS-bmm-RNAi#2* controls. (G) Between 0 DPE and 5 DPE, mRNA expression levels for *bmm* were not significantly increased in virgin *w*^*1118*^ females but were significantly increased in virgin *w*^*1118*^ males (*p* = 0.17 and 0.048, respectively; Student *t* test). (H) Between 0 and 12 hours STV, we observed no triglyceride breakdown in *da>+*, *+>UAS-bmm-RNAi*, or *da>UAS-bmm-RNAi* males (*p* = 0.3, 0.053, and 0.18, respectively; one-way ANOVA followed by Tukey HSD test); however, between 12 and 24 hours STV, the magnitude of triglyceride breakdown in *da>UAS-bmm-RNAi* males was lower than *da>+* and *+>UAS-bmm-RNAi* control males (*p* = 0.038, 3.1 × 10^−6^, and 0.00046, respectively; one-way ANOVA followed by Tukey HSD test). (I) Between 0 and 12 hours STV, and between 12 and 24 hours STV, we observed little change in triglyceride levels in *da>+*, *+>UAS-bmm-RNAi*, or *da>UAS-bmm-RNAi* females (*p* = 0.36, 0.0024, and 0.64, respectively, for 0–12 hours STV and 0.52, 0.046, and 0.045, respectively, for 12–24 hours STV; one-way ANOVA followed by Tukey HSD test). (J) Between 0 and 12 hours STV we observed similar magnitudes of triglyceride breakdown between *da>+*, *+>UAS-bmm-RNAi#2*, and *da>UAS-bmm-RNAi#2* (BDSC #25926) males (*p* = 8.1 × 10^−6^, 1.8 × 10^−6^, and 7.1 × 10^−5^, respectively; one-way ANOVA followed by Tukey HSD test); however, between 12 and 24 hours STV, triglyceride breakdown in *da>UAS-bmm-RNAi#2* (BDSC #25926) males was blocked, whereas triglyceride levels decreased in *da>+* and *+>UAS-bmm-RNAi#2* control males (*p* = 0.069, 1.3 × 10^−6^, and 2 × 10^−7^, respectively; one-way ANOVA followed by Tukey HSD test). (K) Between 0 and 12 hours STV, triglyceride breakdown in *da>UAS-bmm-RNAi#2* (BDSC #25926) females was blocked, whereas triglyceride levels decreased in *da>+* and *+>UAS-bmm-RNAi#2* control females (*p* = 0.18, 0.036, and 9.9 × 10^−5^, respectively; one-way ANOVA followed by Tukey HSD test), whereas we observed similar magnitudes of triglyceride breakdown between 12 and 24 hours STV in *da>+*, *+>UAS-bmm-RNAi#2*, and *da>UAS-bmm-RNAi#2* (BDSC #25926) females (*p* = 0.032, 0.28, and 0.022, respectively; one-way ANOVA followed by Tukey HSD test). Asterisks indicate a significant difference between two sexes, two genotypes, or two time points (*p < 0.05, **p < 0.01, ***p < 0.001). Error bars on graphs depicting percent body fat or mRNA expression level represent SEM; error bars on graphs depicting the change in percent body fat represent COE. See S1 Table for list of all multiple comparisons and *p*-values; quantitative measurements underlying all graphs are available in S1 and S3 Datas. *bmm*, *brummer*; BDSC, Bloomington *Drosophila* Stock Center; COE, coefficient of error; *da*, *daughterless*; DPE, days post-eclosion; F, female; HSD, honest significant difference; M, male; ns, no significant difference between two sexes, two genotypes, or time points; STV, post-starvation; *UAS*, *upstream activation sequence*; *w*, *white*.
(TIF)

S11 Fig Inhibition of *bmm* in the abdominal fat body does not abolish sex differences in whole-body triglyceride storage and breakdown. (A) Whole-body triglyceride storage in 5-day-old virgin males overexpressing *UAS-bmm-RNAi* in the fat body (*cg>UAS-bmm-RNAi*) was significantly higher than age-matched control males (*cg>+* and *+>UAS-bmm-RNAi*) (*p* = 1.0 × 10^−4^ and 8.0 × 10^−7^, respectively; one-way ANOVA followed by Tukey HSD test). (B) Whole-body triglyceride storage in 5-day-old virgin females overexpressing *UAS-bmm-RNAi* in the fat body (*cg>UAS-bmm-RNAi*) was significantly higher than age-matched control females (*cg>+* and *+>UAS-bmm-RNAi*) (*p* = 5.5 × 10^−4^ and 1.0 × 10^−7^, respectively; one-way ANOVA followed by Tukey HSD test). (C) There was a significant reduction in control female and male triglyceride levels (*cg>+*) between 0 and 12 hours post-starvation (*p* = 2.8 × 10^−6^ and 1.0 × 10^−4^, respectively; one-way ANOVA followed by Tukey HSD test); however, we observed no significant reduction in whole-body triglyceride levels in 5-day-old virgin *cg>UAS-bmm-RNAi* females and males between 0 and 12 hours post-starvation (*p* = 0.54 and 0.92, respectively; one-way ANOVA followed by Tukey HSD test). (D) There was a significant reduction in triglyceride storage in control females and males (*cg>+* and *+>UAS-bmm-RNAi)* between 12 and 24 hours post-starvation (*p* = 1 × 10^−7^ and 2.7 × 10^−4^ (females) and *p =* 4.0 × 10^−4^ and 2 × 10^−7^ (males), respectively; one-way ANOVA followed by Tukey HSD test); however, there was no significant change in whole-body triglyceride levels in 5-day-old virgin *cg>UAS-bmm-RNAi* females between 12 and 24 hours post-starvation (*p* = 1.0; one-way ANOVA followed by Tukey HSD test). In males, there was a significant but blunted decrease in triglyceride levels in 5-day-old *cg>UAS-bmm-RNAi* virgin males between 12 and 24 hours post-starvation (*p* = 0.0011; one-way ANOVA followed by Tukey HSD). (E) Whole-body triglyceride storage in 5-day-old *r4>UAS-bmm-RNAi* males was significantly higher than *r4>+* and *+>UAS-bmm-RNAi* control males (*p* = 0.0 and 0.0, respectively; one-way ANOVA followed by Tukey HSD test). (F) *r4>UAS-bmm-RNAi* females did not show a significant difference in whole-body triglyceride storage compared with *r4>+* and +>*UAS-bmm-RNAi* control females (*p* = 0.95 and 0.00015, respectively; one-way ANOVA followed by Tukey HSD test). (G) Between 0 and 12 hours post-starvation, there was no significant decrease in triglyceride levels in either 5-day-old *r4>UAS-bmm-RNAi* or +>*UAS-bmm-RNAi* females (*p* = 0.42 and 0.096, respectively; one-way ANOVA followed by Tukey HSD test) and a modest but significant reduction in triglyceride level in *r4>+* during the same interval (*p =* 0.00014; one-way ANOVA followed by Tukey HSD test). In males, we observed similar trends for *r4>+*, +>*UAS-bmm-RNAi*, and *r4>UAS-bmm-RNAi* males (*p* = 1.0 × 10^−7^, 0.057, and 0.0012, respectively; one-way ANOVA followed by Tukey HSD test). (H) Between 12 and 24 hours post-starvation, we observed that the magnitude of the decrease in triglyceride levels in 5-day-old *r4>UAS-bmm-RNAi* females and males was blunted compared with the decrease in *r4>+* and *+>UAS-bmm-RNAi* controls for each sex (*p* = 0.04, 0.00048, and 2.0 × 10^−7^ for females; 0.0013, 0.12, and 8.2 × 10^−5^ for males, respectively; one-way ANOVA followed by Tukey HSD test). Asterisks indicate a significant difference between two sexes, two genotypes, or two time points (*p < 0.05, **p < 0.01, ***p < 0.001). Error bars on graphs depicting Percent Body Fat represent SEM; error bars on graphs depicting the change in percent body fat represent COE. See S1 Table for list of all multiple comparisons and *p*-values; quantitative measurements underlying all graphs are available in S1 Data. *bmm*, *brummer*; *cg*, *collagen*; F, female; HSD, honest significant difference; M, male; ns, no significant difference between two sexes, two genotypes, or time points; *UAS*, *upstream activation sequence*.
(TIF)

S12 Fig Inhibition of *bmm* in the gut, muscle, or glia does not alter whole-body triglyceride storage in either sex. (A) Whole-body triglyceride storage in 5-day-old virgin females overexpressing *UAS-bmm-RNAi* in the gut (*Mex>UAS-bmm-RNAi*) was not significantly different from age-matched control females (*Mex>+* and *+>UAS-bmm-RNAi*) (*p* = 0.31 and 0.0073, respectively; one-way ANOVA followed by Tukey HSD test). (B) Whole-body triglyceride storage in 5-day-old virgin males overexpressing *UAS-bmm-RNAi* in the gut (*Mex>UAS-bmm-RNAi*) was not significantly different from age-matched control males (*Mex>+* and *+>UAS-bmm-RNAi*) (*p* = 0.17 and 0.079, respectively; one-way ANOVA followed by Tukey HSD test). (C) Whole-body triglyceride storage in 5-day-old virgin females overexpressing *UAS-bmm-RNAi* in the muscle (*dMef2>UAS-bmm-RNAi*) was not significantly different from age-matched control females (*dMef2>+* and *+>UAS-bmm-RNAi*) (*p* = 0.50 and 0.70, respectively; one-way ANOVA followed by Tukey HSD test). (D) Whole-body triglyceride storage in 5-day-old virgin males overexpressing *UAS-bmm-RNAi* in the muscle (d*Mef2>UAS-bmm-RNAi*) was not significantly different from age-matched control males (d*Mef2>+* and *+>UAS-bmm-RNAi*) (*p* = 0.54 and 0.34, respectively; one-way ANOVA followed by Tukey HSD test). (E) Whole-body triglyceride level in 5-day-old virgin females overexpressing *UAS-bmm-RNAi* in the glia (*repo>UAS-bmm-RNAi*) was not significantly different from age-matched control females (*repo>+* and *+>UAS-bmm-RNAi*) (*p* = 3.2 × 10^−5^ and 0.26, respectively; one-way ANOVA followed by Tukey HSD test). (F) Whole-body triglyceride levels in 5-day-old virgin males overexpressing *UAS-bmm-RNAi* in the glia (*repo>UAS-bmm-RNAi*) were not significantly different from age-matched control males (*repo>+* and *+>UAS-bmm-RNAi*) (*p* = 0.016 and 0.8, respectively; one-way ANOVA followed by Tukey HSD test). The *p*-values are listed in the following order: difference between the *GAL4*/*UAS* genotype and the *GAL4* control and difference between the *GAL4*/*UAS* genotype and the *UAS* control, respectively. Asterisks indicate a significant difference between two sexes, two genotypes, or two time points (*p < 0.05, **p < 0.01, ***p < 0.001). Error bars on graphs represent SEM. See S1 Table for a list of all multiple comparisons and *p*-values; quantitative measurements underlying all graphs are available in S1 Data. *bmm*, *brummer*; F, female; HSD, honest significant difference; M, male; *Mex*, *midgut expression 1*; *Mef2*, *myocyte enhancer factor 2*; ns, no significant difference between two sexes, two genotypes, or time points; *repo*, *reversed polarity*; *UAS*, *upstream activation sequence*.
(TIF)

S13 Fig Loss of *bmm* function in the somatic cells of the gonad with an independent RNAi line affects whole-body triglyceride storage and breakdown in males. (A) Whole-body triglyceride storage in males with *c587-GAL4*-mediated overexpression of an additional *UAS-bmm-RNAi#2* (VDRC #37877) transgene in the somatic cells of the male gonad was significantly higher than in control males (*c587-GAL4>+* and *+>UAS-bmm-RNAi#2*) (*p* = 2.3 × 10^−5^ and 1.1 × 10^−5^, respectively; one-way ANOVA followed by Tukey HSD test). (B) The decrease in whole-body triglyceride levels in *c587-GAL4>UAS-bmm-RNAi#2* (VDRC #37877) males was blunted compared with *c587-GAL4>+* and *+>UAS-bmm-RNAi#2* control males between 0 and 12 hours STV (*p* = 0.47, 5.8 × 10^−6^, and 0.065, respectively; one-way ANOVA followed by Tukey HSD test) but not between 12 and 24 hours STV (*p* = 8.2 × 10^−4^, 6.0 × 10^−7^, and 0.0033, respectively; one-way ANOVA followed by Tukey HSD test). (C) Whole-body triglyceride levels in 5-day-old virgin *c587>UAS-bmm;UAS-bmm-RNAi* males were not significantly different to *c587-GAL4>+* and *+>UAS-bmm;UAS-bmm-RNAi* controls, demonstrating that re-expression of *UAS-bmm* rescued the increased fat storage caused by loss of *bmm* in the somatic cells of the male gonad (*p* = 0.87 and 1.0, respectively; one-way ANOVA followed by Tukey HSD test). (D) Triglyceride breakdown post-starvation among 5-day-old virgin *c587>UAS-bmm;UAS-bmm-RNAi* males and control males (*c587>+* and *+>UAS-bmm;UAS-bmm-RNAi*) was decreased by a similar magnitude between both 0 and 12 hours or 12 and 24 hours STV, demonstrating that re-expression of *UAS-bmm* rescued the effects of *bmm* loss in the somatic cells of the male gonad post-starvation (*p* = 2.2 × 10^−6^, 1.7 × 10^−6^, and 0.0 for 0–12 hours and 0.0, 0.0, and 0.0 for 12–24 hours, respectively; one-way ANOVA followed by Tukey HSD test). Asterisks indicate a significant difference between two sexes, two genotypes, or two time points (*p < 0.05, **p < 0.01, ***p < 0.001). Error bars on graphs depicting percent body fat represent SEM; error bars on graphs depicting the change in percent body fat represent COE. See S1 Table for a list of all multiple comparisons and *p*-values; quantitative measurements underlying all graphs are available in S1 Data. *bmm*, *brummer*; COE, coefficient of error; HSD, honest significant difference; M, male; ns, no significant difference between two sexes, two genotypes, or time points; STV, post-starvation; *UAS*, *upstream activation sequence*; VDRC, Vienna *Drosophila* Resource Center.
(TIF)

S14 Fig Loss of *bmm* in the somatic cells of the gonad with an independent RNAi line has no effect on triglyceride storage or breakdown in females. (A) Whole-body triglyceride storage in 5-day-old virgin females overexpressing *UAS-bmm-RNAi* in the somatic cells of the gonad (*c587>UAS-bmm-RNAi*) was not significantly different from age-matched control females (*c587>+* and *+>UAS-bmm-RNAi*) (*p* = 0.083 and 0.96, respectively; one-way ANOVA followed by Tukey HSD test). (B) There was a modest but significant reduction in whole-body triglyceride levels in 5-day-old *c587>+*, *+>UAS-bmm-RNAi*, and *c587>UAS-bmm-RNAi* females between 0 and 12 hours STV (*p* = 2.3 × 10^−4^, 0.0094, and 0.0051, respectively; one-way ANOVA followed by Tukey HSD test) and between 12 and 24 hours STV (*p* = 0.0, 0.016, and 1.6 × 10^−5^, respectively; one-way ANOVA followed by Tukey HSD test). (C) Whole-body triglyceride storage in females with *c587-GAL4*-mediated overexpression of an additional *UAS-bmm-RNAi#2* (VDRC #37877) transgene in the somatic cells of the gonad was not significantly different from control females (*c587-GAL4>+* and *+>UAS-bmm-RNAi#2*) (*p* = 0.98 and 0.78, respectively; one-way ANOVA followed by Tukey HSD test). (D) Triglyceride breakdown post-starvation among *c587-GAL4>UAS-bmm-RNAi#2* (VDRC #37877) females and *c587-GAL4>+* and *+>UAS-bmm-RNAi#*2 controls showed a modest decrease of similar magnitude between both 0 and 12 hours and 12 and 24 hours STV (*p* = 0.096, 1.3 × 10^−4^, and 0.0013 for 0–12 hours and 0.004, 0.0049, and 0.022 for 12–24 hours, respectively; one-way ANOVA followed by Tukey HSD test). (E) Whole-body triglyceride levels in 5-day-old virgin *c587>UAS-bmm;UAS-bmm-RNAi* females were not significantly different among *c587-GAL4>+* and *+>UAS-bmm;UAS-bmm-RNAi* controls (*p* = 8.5 × 10^−4^ and 0.38, respectively; one-way ANOVA followed by Tukey HSD test). (F) Triglyceride breakdown post-starvation among 5-day-old virgin *c587>UAS-bmm;UAS-bmm-RNAi* females and control females (*c587*>+ and *+>UAS-bmm;UAS-bmm-RNAi*) showed a modest decrease of a similar magnitude at both 0–12 hours or 12–24 hours STV (*p* = 0.0043, 1.7 × 10^−6^, and 0.0027 for 0–12 hours and 0.0, 2.6 × 10^−6^, and 2.9 × 10^−4^ for 12–24 hours, respectively; one-way ANOVA followed by Tukey HSD test). Asterisks indicate a significant difference between two sexes, two genotypes, or two time points (*p < 0.05, **p < 0.01, ***p < 0.001). Error bars on graphs depicting percent body fat represent SEM; error bars on graphs depicting the change in percent body fat represent COE. See S1 Table for a list of all multiple comparisons and *p*-values; quantitative measurements underlying all graphs are available in S1 Data. *bmm*, *brummer*; COE, coefficient of error; F, female; HSD, honest significant difference; ns, no significant difference between two sexes, two genotypes, or time points; STV, post-starvation; *UAS*, *upstream activation sequence*; VDRC, Vienna *Drosophila* Resource Center.
(TIF)

S15 Fig Loss of *bmm* function in the somatic cells of the gonad with an independent GAL4 line affects whole-body triglyceride breakdown in males but not females. (A) Whole-body triglyceride storage in 5-day-old virgin males overexpressing *UAS-bmm-RNAi* in the somatic cells of the gonad (*tj>UAS-bmm-RNAi*) was not significantly different from age-matched control males (*tj>+* and *+>UAS-bmm-RNAi*) (*p* = 0.098 and 0.97, respectively; one-way ANOVA followed by Tukey HSD test). (B) Whole-body triglyceride storage in 5-day-old virgin females overexpressing *UAS-bmm-RNAi* in the somatic cells of the gonad (*tj>UAS-bmm-RNAi*) was not significantly different from age-matched control females (*tj>+* and *+>UAS-bmm-RNAi*) (*p* = 0.2 and 0.66, respectively; one-way ANOVA followed by Tukey HSD test). (C) Between 0 and 12 hours STV, the magnitude of triglyceride breakdown in *tj>UAS-bmm-RNAi* was similar to *tj>+* and *+>UAS-bmm-RNAi* control males; however, between 12 and 24 hours STV, there was no significant decrease in triglyceride levels in *tj>UAS-bmm-RNAi* males, in contrast to *tj>+* and *+>UAS-bmm-RNAi* control males, in which we observed a significant decrease in triglyceride storage STV (*p* = 9.7 × 10^−4^, 0.0018, and 0.099 for 0–12 hours and 0.37, 1.9 × 10^−5^, and 0.028 for 12–24 hours, respectively; one-way ANOVA followed by Tukey HSD test). (D) Triglyceride breakdown post-starvation among 5-day-old virgin *tj>UAS-bmm-RNAi* females and *tj>+* and *+>UAS-bmm-RNAi* control females was modestly decreased by a similar magnitude at both 0–12 hours or 12–24 hours STV (*p* = 0.058, 0.026, and 0.022 for 0–12 hours and 0.042, 0.0093, and 3.7 × 10^−4^ for 12–24 hours, respectively; one-way ANOVA followed by Tukey HSD test). (E–G) We used *tj-GAL4* to drive the expression of a membrane-bound GFP (*UAS-mCD8*::*GFP*) in the somatic cells of the gonad. The presence of lipid droplets within the GFP-marked boundary of the somatic cell indicates that lipid droplets are present in the somatic cells of the gonad. Non-GFP-positive droplets (arrow) likely represent lipid droplets in the germline cells. The image represents a single confocal slice from the *Drosophila* male testis. Scale bars = 50 μm, except for inset images, in which scale bars = 12.5 μm. Asterisks indicate a significant difference between two sexes, two genotypes, or two time points (*p < 0.05; **p < 0.01, ***p < 0.001). Error bars on graphs depicting percent body fat represent SEM; error bars on graphs depicting the change in percent body fat represent COE. See S1 Table for a list of all multiple comparisons and *p*-values; quantitative measurements underlying all graphs are available in S1 Data. Original image files corresponding to all images acquired from genotype-matched individuals presented in panels E–G are available upon request. *bmm*, *brummer*; COE, coefficient of error; F, female; GFP, green fluorescent protein; *tj*, *traffic jam*; HSD, honest significant difference; M, male; ns, no significant difference between two sexes, two genotypes, or time points; STV, post-starvation; *UAS*, *upstream activation sequence*.
(TIF)

S16 Fig Loss of *bmm* function in neurons with an independent RNAi line and additional neuronal GAL4 line affects whole-body triglyceride breakdown in males. (A) Whole-body triglyceride storage in males with *elav-GAL4*-mediated overexpression of an additional *UAS-bmm-RNAi#2* (VDRC #37877) transgene in neurons was not significantly different than in control males (*elav-GAL4>+* and *+>UAS-bmm-RNAi#2*) (*p* = 0.11 and 0.91, respectively; one-way ANOVA followed by Tukey HSD test). (B) Whole-body triglyceride storage in males with *nSyb-GAL4*-mediated overexpression of the *UAS-bmm-RNAi* transgene in neurons was not significantly different than in control males (*nSyb-GAL4>+* and *+>UAS-bmm-RNAi*) (*p* = 0.19 and 2.1 × 10^−4^, respectively; one-way ANOVA followed by Tukey HSD test). (C) The decrease in whole-body triglyceride levels in *elav-GAL4>UAS-bmm-RNAi#2* (VDRC #37877) males was similar to *elav-GAL4>+* and *+>UAS-bmm-RNAi#2* control males between 0 and 12 hours STV (*p* = 0.01, 4.0 × 10^−7^, and 1.0 × 10^−5^, respectively; one-way ANOVA followed by Tukey HSD test). Triglyceride breakdown between 12 and 24 hours STV was blocked in *elav-GAL4>UAS-bmm-RNAi#2* males, whereas triglyceride levels in control males during this interval significantly decreased (*p* = 0.084, 2.6 × 10^−5^, and 3.3 × 10^−4^, respectively; one-way ANOVA followed by Tukey HSD test). (D) The decrease in whole-body triglyceride levels in *nSyb-GAL4>UAS-bmm-RNAi* males was blunted compared with *nSyb-GAL4>+* and *+>UAS-bmm-RNAi* control males between 0 and 12 hours STV (*p* = 0.86, 0.051, and 4.3 × 10^−5^, respectively; one-way ANOVA followed by Tukey HSD test); however, triglyceride breakdown between 12 and 24 hours STV in *nSyb-GAL4>UAS-bmm-RNAi* males was similar in magnitude to control males (*nSyb>+* and *+>UAS-bmm-RNAi*) (*p* = 0.002, 1.2 × 10^−4^, and 7.0 × 10^−4^, respectively; one-way ANOVA followed by Tukey HSD test). (E) In dissected brains from *elav>UAS-bmm-RNAi* males, we found that *bmm* transcript levels were undetectable in three out of four samples, whereas we observed amplification at a higher cycle number in three *elav>+* samples. (§) Because of this dramatic decrease in *bmm* transcript levels in most *elav>UAS-bmm-RNAi* samples, we were unable to perform a statistical analysis to quantify this effect. (F) Whole-body triglyceride levels in 5-day-old virgin *elav>UAS-bmm;UAS-bmm-RNAi* males were not significantly different to *elav>+* and *+>UAS-bmm;UAS-bmm-RNAi* controls (*p* = 0.84 and 0.92, respectively; one-way ANOVA followed by Tukey HSD test). (G) Whole-body triglyceride levels post-starvation among 5-day-old virgin *elav>UAS-bmm;UAS-bmm-RNAi* males and control males (*elav>+* and *+>UAS-bmm;UAS-bmm-RNA*) were significantly decreased by a similar magnitude at both 0–12 hours or 12–24 hours STV, demonstrating that re-expression of *UAS-bmm* rescued the effects of *bmm* loss in neurons STV (*p* = 2.3 × 10^−6^, 1.2 × 10^−4^, and 0.0 for 0–12 hours and 2.0 × 10^−7^, 1.0 × 10^−7^, and 0.0 for 12–24 hours, respectively; one-way ANOVA followed by Tukey HSD test). Asterisks indicate a significant difference between two sexes, two genotypes, or two time points (*p < 0.05, **p < 0.01, ***p < 0.001). Error bars on graphs depicting percent body fat or mRNA expression level represent SEM; error bars on graphs depicting the change in percent body fat represent COE. See S1 Table for a list of all multiple comparisons and *p*-values; quantitative measurements underlying all graphs are available in S1 and S3 Datas. *bmm*, *brummer*; COE, coefficient of error; *elav*, *embryonic lethal abnormal vision*; HSD, honest significant difference; M, male; ns, no significant difference between two sexes, two genotypes, or time points; *nSyb*, *neuronal Synaptobrevin*; STV, post-starvation; *UAS*, *upstream activation sequence*; VDRC, Vienna *Drosophila* Resource Center.
(TIF)

S17 Fig Inhibition of *bmm* in glia does not alter whole-body triglyceride breakdown. (A) Triglyceride breakdown post-starvation was modestly decreased by a similar magnitude in 5-day-old virgin *repo>UAS-bmm-RNAi* females compared with *repo-GAL4>+* and *+>UAS-bmm-RNAi* control females at 0–12 hours post-starvation (*p* = 0.047, 2.3 × 10^−5^, and 0.096, respectively; one-way ANOVA followed by Tukey HSD test) and between *repo>UAS-bmm-RNAi* males and *repo-GAL4>+* and *+>UAS-bmm-RNAi* control males during the same interval (*p* = 0.085, 6.9 × 10^−6^, and 0.057, respectively; one-way ANOVA followed by Tukey HSD test). (B) Between 12 and 24 hours post-starvation, we observed only a modest reduction in female triglyceride levels post-starvation in all genotypes (*repo>UAS-bmm-RNAi*, *repo-GAL4>+*, and *+>UAS-bmm-RNAi*), and the magnitude of this reduction was similar between genotypes (*p* = 0.0019, 4.8 × 10^−6^, and 2.0 × 10^−7^, respectively; one-way ANOVA followed by Tukey HSD test). Similarly, although there was a significant decrease in triglyceride levels post-starvation in *repo>UAS-bmm-RNAi* males, *repo-GAL4>+*, and *+>UAS-bmm-RNAi* controls (*p* = 8.8 × 10^−6^, 0.0, and 8.2 × 10^−5^, respectively; one-way ANOVA followed by Tukey HSD test), the magnitude of this decrease was similar for all genotypes. Asterisks indicate a significant difference between two sexes, two genotypes, or two time points (*p < 0.05, **p < 0.01, ***p < 0.001). See S1 Table for list of all multiple comparisons and *p*-values. Error bars on graphs represent COE. Quantitative measurements underlying all graphs are available in S1 Data. *bmm*, *brummer*; COE, coefficient of error; HSD, honest significant difference; ns indicates no significant difference between two sexes, two genotypes, or time points; *repo*, *reversed polarity*; *UAS*, *upstream activation sequence*.
(TIF)

S18 Fig Loss of *bmm* in the neurons with an independent RNAi line has no effect on triglyceride storage or breakdown in females. (A) Whole-body triglyceride storage in 5-day-old virgin females overexpressing *UAS-bmm-RNAi* in the postmitotic neurons (*elav>UAS-bmm-RNAi*) was not significantly different from age-matched control females (*elav>+* and *+>UAS-bmm-RNAi*) (*p* = 0.54 and 0.95, respectively; one-way ANOVA followed by Tukey HSD test). (B) There was a significant reduction in whole-body triglyceride levels in 5-day-old *elav>+*, *+>UAS-bmm-RNAi*, and *elav>UAS-bmm-RNAi* females between 0 and 12 hours STV (*p* = 0.026, 0.0038, and 0.0013, respectively; one-way ANOVA followed by Tukey HSD test). Between 12 and 24 hours STV, 5-day-old *elav>+*, *+>UAS-bmm-RNAi*, and *elav>UAS-bmm-RNAi* females modestly decreased triglyceride levels by similar magnitudes (*p* = 1.2 × 10^−4^, 0.15, and 0.0012, respectively; one-way ANOVA followed by Tukey HSD test). (C) Whole-body triglyceride storage in females with *elav-GAL4*-mediated overexpression of an additional *UAS-bmm-RNAi#2* (VDRC #37877) transgene in neurons was not significantly different from control females (*elav-GAL4>+* and *+>UAS-bmm-RNAi#2*) (*p* = 0.56 and 0.035, respectively; one-way ANOVA followed by Tukey HSD test). (D) There was a modest decrease in triglyceride levels between 0 and 12 hours and 12 and 24 hours STV among *elav-GAL4>UAS-bmm-RNAi#2* (VDRC #37877) females and *elav-GAL4>+* and *+>UAS-bmm-RNAi#*2 controls (*p* = 0.011, 0.0027, and 1.4 × 10^−6^ for 0–12 hours STV and 4.6 × 10^−4^, 3.4 × 10^−4^, and 0.002 for 12–24 hours STV, respectively; one-way ANOVA followed by Tukey HSD test). (E) Whole-body triglyceride levels in 5-day-old virgin *nSyb>UAS-bmm-RNAi* females were not significantly different to *nSyb-GAL4>+* and *+>UAS-bmm-RNAi* controls (*p* = 0.85 and 0.52, respectively; one-way ANOVA followed by Tukey HSD test). (F) Triglyceride breakdown post-starvation was modestly decreased by a similar magnitude among genotypes in 5-day-old virgin *nSyb>UAS-bmm-RNAi* females and control females (*nSyb>+* and *+>UAS-bmm-RNAi*) between 0 and 12 hours STV (*p* = 0.21, 0.017, and 0.43, respectively; one-way ANOVA followed by Tukey HSD test). Between 12 and 24 hours STV, the decrease in triglyceride levels in *nSyb>UAS-bmm-RNAi* females was blocked, whereas triglyceride levels decreased in control *nSyb-GAL4>+* and *+>UAS-bmm-RNAi* females during this interval (*p* = 0.093, 3.0 × 10^−5^, and 0.0019, respectively; one-way ANOVA followed by Tukey HSD test). (G) Whole-body triglyceride levels in 5-day-old virgin *elav>UAS-bmm;UAS-bmm-RNAi* females were not significantly different to *elav-GAL4>+* and *+>UAS-bmm;UAS-bmm-RNAi* controls (*p* = 0.41 and 0.66, respectively; one-way ANOVA followed by Tukey HSD test). (H) Whole-body triglyceride levels post-starvation among 5-day-old virgin *elav>UAS-bmm;UAS-bmm-RNAi* females and control females (*elav>+* and *+>UAS-bmm;UAS-bmm-RNAi*) decreased by a similar magnitude between 0 and 12 hours or 12 and 24 hours STV, demonstrating that re-expression of *UAS-bmm* rescued the effects of *bmm* loss in neurons STV (*p* = 0.0023, 0.0012, and 0.0027 for 0–12 hours and 1.0 × 10^−6^, 5.2 × 10^−6^, and 2.9 × 10^−4^ for 12–24 hours, respectively; one-way ANOVA followed by Tukey HSD test). Asterisks indicate a significant difference between two sexes, two genotypes, or two time points (*p < 0.05, **p < 0.01, ***p < 0.001). Error bars on graphs depicting percent body fat represent SEM; error bars on graphs depicting the change in percent body fat represent COE. See S1 Table for a list of all multiple comparisons and *p*-values; quantitative measurements underlying all graphs are available in S1 Data. *bmm*, *brummer*; COE, coefficient of error; *elav*, *embryonic lethal abnormal vision*; F, female; HSD, honest significant difference; ns, no significant difference between two sexes, two genotypes, or time points; *nSyb*, *neuronal Synaptobrevin*; STV, post-starvation; *UAS*, *upstream activation sequence*; VDRC, Vienna *Drosophila* Resource Center.
(TIF)

S19 Fig *bmm* inhibition in the whole-body affects starvation resistance in both sexes. (A) Median survival post-starvation was significantly higher in 5-day-old virgin *Oregon-R* females than in virgin *Oregon-R* males (*p* = 2 × 10^−16^; Log-rank test with Bonferroni correction for multiple comparison; *n* > 156). (B) Median survival post-starvation was significantly higher in 5-day-old virgin *CMW* wild-caught females than in virgin *CMW* males (*p* = 6 × 10^−10^; Log-rank test with Bonferroni correction for multiple comparison; *n* > 118). (C, D) Median survival post-starvation was significantly higher in 5-day-old virgin females than males in two isofemale strains (*Mel c2*.*2*: *p* = 4.4 × 10^−11^; Log-rank test with Bonferroni correction for multiple comparison; *n* > 55 and *Mel c2*.*3*: *p =* 1.4 × 10^−13^; Log-rank test with Bonferroni correction for multiple comparison; *n* > 110). (E, F) Median survival post-starvation was significantly higher in virgin males (E) and females (F), with ubiquitous overexpression of *UAS-bmm-RNAi* compared with control males (*da>+* and *+>UAS-bmm-RNAi*) (*p* = 2 × 10^−16^ and 2 × 10^−16^ respectively; Log-rank test with Bonferroni correction for multiple comparison; n > 223) and females (*p* = 2 × 10^−16^ and 1.2 × 10^−13^ respectively; Log-rank test with Bonferroni correction for multiple comparisons; n > 176). (G, H) Median survival post-starvation was significantly higher in virgin males (G) and significantly lower in females (H) with ubiquitous overexpression of *UAS-bmm-RNAi#2* (BDSC #25926) compared to control males (*da>+* and *+>UAS-bmm-RNAi#2*) (*p* = 7.7 × 10^−11^ and 2 × 10^−16^, respectively; Log-rank test with Bonferroni correction for multiple comparison; *n* > 97) and control females (*p* = 9.6 × 10^−9^ and 4.9 × 10^−5^, respectively; Log-rank test with Bonferroni correction for multiple comparisons; *n* > 91). The *p*-values are listed in the following order: difference between the *GAL4*/*UAS* genotype and the *GAL4* control/difference between the *GAL4*/*UAS* genotype and the *UAS* control. Asterisks indicate a significant difference between two sexes, two genotypes, or two time points (*p < 0.05, **p < 0.01, ***p < 0.001). Shaded areas represent the 95% confidence interval. See S1 Table for a list of all multiple comparisons and *p*-values; quantitative measurements underlying all graphs are available in S4 Data. *bmm*, *brummer*; BDSC, Bloomington *Drosophila* Stock Center; *CMW*, *Country Mill Winery*; *da*, *daughterless*; F, female; M, male; ns, no significant difference between two sexes, two genotypes, or time points; *UAS*, *upstream activation sequence*.
(TIF)

S20 Fig *brummer* expression in males is necessary for maintaining male fertility. (A) Males with whole-body loss of *brummer* have a significantly decreased number of progeny after 2 days, 4 days, and 6 days of mating (*p* = 2.2 × 10^−16^, 9.7 × 10^−12^, and 4.6 × 10^−6^, respectively; Student *t* test at each time point). Asterisks indicate a significant difference between two sexes, two genotypes, or two time points (*p < 0.05, **p < 0.01, ***p < 0.001). See S1 Table for list of all multiple comparisons and *p*-values. Error bars on graphs represent SEM. Quantitative measurements underlying all graphs are available in S4 Data. M, male; ns, no significant difference between two sexes, two genotypes, or time points.
(TIF)

S21 Fig *bmm* inhibition in the fat body affects starvation resistance in both sexes. (A, B) Median survival post-starvation was significantly higher in virgin females (A) and males (B) with fat body–specific *bmm* inhibition (*cg>UAS-bmm-RNAi*) compared with control females (*cg>+* and *+>UAS-bmm-RNAi*) (*p* = 2 × 10^−16^ and 2 × 10^−16^, respectively; Log-rank test with Bonferroni correction for multiple comparisons; *n* > 279) and males (*p* = 2 × 10^−16^ and 2 × 10^−16^, respectively; Log-rank test with Bonferroni correction for multiple comparisons; *n* > 365). (C, D) Median survival post-starvation was significantly higher in virgin females (C) and males (D) with fat body–specific *bmm* inhibition using a second GAL4 driver (*r4>UAS-bmm-RNAi*) compared with control females (*r4>+* and *+>UAS-bmm-RNAi*) (*p* = 2 × 10^−16^ and 2 × 10^−16^, respectively; Log-rank test with Bonferroni correction for multiple comparisons; *n* > 314) and males (*p* = 2 × 10^−16^ and 2 × 10^−16^, respectively; Log-rank test with Bonferroni correction for multiple comparisons; *n* > 195). The *p*-values are listed in the following order: difference between the *GAL4*/*UAS* genotype and the *GAL4* control/difference between the *GAL4*/*UAS* genotype and the *UAS* control. Asterisks indicate a significant difference between two sexes, two genotypes, or two time points (*p < 0.05, **p < 0.01, ***p < 0.001). Shaded areas represent the 95% confidence interval. See S1 Table for list of all multiple comparisons and *p*-values; quantitative measurements underlying all graphs are available in S4 Data. *bmm*, *brummer*; *cg*, *collagen*; F, female; M, male; ns, no significant difference between two sexes, two genotypes, or time points; *UAS*, *upstream activation sequence*.
(TIF)

S22 Fig *bmm* inhibition in the somatic cells of the gonad affects starvation resistance in males. (A, B) Median survival post-starvation was significantly higher in virgin males (A) but not significantly different in virgin females (B) with *bmm* inhibition in the somatic cells of the gonad (*c587>UAS-bmm-RNAi#2* [VDRC #37877]) compared with control males (*c587>+* and *+>UAS-bmm-RNAi#2*) (*p* = 2 × 10^−16^ and 2 × 10^−16^, respectively; Log-rank test with Bonferroni correction for multiple comparisons; *n* > 212) and females (*p* = 2 × 10^−16^ and 1.0, respectively; Log-rank test with Bonferroni correction for multiple comparisons; *n* > 201). (C, D) Median survival post-starvation was not significantly changed when *UAS-bmm* and *UAS-bmm-RNAi* were simultaneously overexpressed by a driver for the somatic cells of the gonad (*c587>UAS-bmm;UAS-bmm-RNAi*) in both males (C) and females (D) compared with control males (*c587>+* and *+>UAS-bmm;UAS-bmm-RNAi*) (*p* = 2 × 10^−16^ and 0.57, respectively; Log-rank test with Bonferroni correction for multiple comparisons; *n* > 249) and females (*p* = 9.6 × 10^−16^ and 0.0035, respectively; Log-rank test with Bonferroni correction for multiple comparisons; *n* > 207). (E, F) Median survival post-starvation was significantly increased when *UAS-bmm-RNAi* was overexpressed by a second driver for the somatic cells of the gonad (*tj>UAS-bmm-RNAi*) in both females (E) and males (F) compared with control males (*tj>+* and +>*UAS-bmm-RNAi*) (*p* = 2 × 10^−16^ and 1.3 × 10^−6^, respectively; Log-rank test with Bonferroni correction for multiple comparisons; *n* > 165) and females (*p* = 2 × 10^−16^ and 0.00012, respectively; Log-rank test with Bonferroni correction for multiple comparisons; *n* > 177). The *p*-values are listed in the following order: difference between the *GAL4*/*UAS* genotype and the *GAL4* control/difference between the *GAL4*/*UAS* genotype and the *UAS* control. Asterisks indicate a significant difference between two sexes, two genotypes, or two time points (*p < 0.05, **p < 0.01, ***p < 0.001). Shaded areas represent the 95% confidence interval. See S1 Table for a list of all multiple comparisons and *p*-values; quantitative measurements underlying all graphs are available in S4 Data. *bmm*, *brummer*; F, female; M, male; ns, no significant difference between two sexes, two genotypes, or time points; *tj*, *traffic jam*; *UAS*, *upstream activation sequence*; VDRC, Vienna *Drosophila* Resource Center.
(TIF)

S23 Fig *bmm* inhibition in neurons affects starvation resistance in males. (A, B) Median survival post-starvation was significantly higher in virgin males (A) but not significantly different in virgin females (B) with *bmm* inhibition in the neurons (*elav>UAS-bmm-RNAi#2* [VDRC #37877]) compared with control males (*elav>+* and *+>UAS-bmm-RNAi#2*) (*p* = 2 × 10^−16^ and 2 × 10^−16^, respectively; Log-rank test with Bonferroni correction for multiple comparisons; *n* > 90) and females (*p* = 3.4 × 10^−6^ and 0.41, respectively; Log-rank test with Bonferroni correction for multiple comparisons; *n* > 81). (C, D) Median survival post-starvation was not significantly changed when *UAS-bmm* and *UAS-bmm-RNAi* were simultaneously overexpressed by a neuronal driver (*elav>UAS-bmm;UAS-bmm-RNAi*) in both males (C) and females (D) compared with control males (*elav>+* and *+>UAS-bmm;UAS-bmm-RNAi*) (*p* = 4.6 × 10^−5^ and 1, respectively; Log-rank test with Bonferroni correction for multiple comparisons; *n* > 101) and females (*p* = 8.9 × 10^−6^ and 1, respectively; Log-rank test with Bonferroni correction for multiple comparisons; *n* > 23). (E, F) Median survival post-starvation was significantly increased when *UAS-bmm-RNAi* was overexpressed by a second neuronal driver (*nSyb>UAS-bmm-RNAi*) in both males (E) and females (F) compared with control males (*nSyb>+* and +>*UAS-bmm-RNAi*) (*p* = 2 × 10^−16^ and 2 × 10^−16^, respectively; Log-rank test with Bonferroni correction for multiple comparisons; *n* > 157) and females (*p* = 2 × 10^−16^ and 2 × 10^−16^, respectively; Log-rank test with Bonferroni correction for multiple comparisons; *n* > 157). The *p*-values are listed in the following order: difference between the *GAL4*/*UAS* genotype and the *GAL4* control/difference between the *GAL4*/*UAS* genotype and the *UAS* control. Asterisks indicate a significant difference between two sexes, two genotypes, or two time points (*p < 0.05, **p < 0.01, ***p < 0.001). Shaded areas represent the 95% confidence interval. See S1 Table for list of all multiple comparisons and *p*-values; quantitative measurements underlying all graphs are available in S4 Data. *bmm*, *brummer*; *elav*, *embryonic lethal abnormal vision*; F, female; M, male; ns indicates no significant difference between two sexes, two genotypes, or time points; *nSyb*, *neuronal Synaptobrevin*; *UAS*, *upstream activation sequence*; VDRC, Vienna *Drosophila* Resource Center.
(TIF)

S24 Fig *bmm* function in the gut, muscle, and glia do not affect starvation resistance. (A, B) Median survival post-starvation showed no significant change in virgin females (A) and males (B) with gut-specific *bmm* inhibition (*Mex>UAS-bmm-RNAi*) compared with control females (*Mex>+* and *+>UAS-bmm-RNAi*) (*p* = 2 × 10^−16^ and 1.1 × 10^−7^, respectively; Log-rank test with Bonferroni correction for multiple comparison; *n* > 444) and males (*p* = 2 × 10^−16^ and 0.13 respectively; Log-rank test with Bonferroni correction for multiple comparison; *n* > 456). (C, D) Median survival post-starvation was unchanged in virgin females (C) and slightly increased in males (D) with muscle-specific *bmm* inhibition (*dMef2>UAS-bmm-RNAi*) compared with control females (*dMef2>+* and *+>UAS-bmm-RNAi*) (*p* = 2 × 10^−16^ and 0.4, respectively; Log-rank test with Bonferroni correction for multiple comparison; *n* > 340) and males (*p* = 2 × 10^−16^ and 2 × 10^−16^, respectively; Log-rank test with Bonferroni correction for multiple comparison; *n* > 382). (E, F) Median survival post-starvation showed no significant change in virgin females (E) and males (F) with glia-specific *bmm* inhibition (*repo>UAS-bmm-RNAi*) compared with control females (*repo>+* and *+>UAS-bmm-RNAi*) (*p* = 0.43 and 2 × 10^−16^, respectively; Log-rank test with Bonferroni correction for multiple comparison; *n* > 191) and males (*p* = 2 × 10^−16^ and 0.02, respectively; Log-rank test with Bonferroni correction for multiple comparison; *n* > 207). The *p*-values are listed in the following order: difference between the *GAL4*/*UAS* genotype and the *GAL4* control/difference between the *GAL4*/*UAS* genotype and the *UAS* control. Asterisks indicate a significant difference between two sexes, two genotypes, or two time points (*p < 0.05, **p < 0.01, ***p < 0.001). Shaded areas represent the 95% confidence interval. See S1 Table for a list of all multiple comparisons and *p*-values; quantitative measurements underlying all graphs are available in S4 Data. *bmm*, *brummer*; F, female; M, male; *Mex*, *midgut expression 1*; *dMef2*, *myocyte enhancer factor 2*, ns, no significant difference between two sexes, two genotypes, or time points; *repo*, *reversed polarity*; *UAS*, *upstream activation sequence*.
(TIF)

S1 Table Details of the statistical analysis for all figures. (XLSX)

S2 Table Details of statistical analysis for fat storage in wild-type genotypes. (XLSX)

S3 Table Description of GAL4-driven GFP expression in multiple tissues. GFP, green fluorescent protein.
(XLSX)

S1 Data Quantitative measurements underlying all macronutrient and lipid droplet data. (XLSX)

S2 Data Quantitative measurements for all CO_2_ production and O_2_ consumption experiments. (CSV)

S3 Data Quantitative measurements underlying all gene expression experiments. (XLSX)

S4 Data Quantitative measurements underlying all life span, fertility, and starvation resistance experiments. (XLSX)

S1 Materials and Methods Additional methodological detail for all experimental procedures. (DOCX)

## Abbreviations

AGPAT: 1-acylglycerol-3-phosphate *O*-acyltransferase
AKH: adipokinetic hormone
ATGL: adipose triglyceride lipase
bmm: brummer
BODIPY: boron-dipyrromethene
cg: collagen
CMW: Country Mill Winery
CNS: central nervous system
CS: Canton-S
da: daughterless
DAGAT: diacylglycerol acyltransferase
DGAT: diacylglycerol *O*-acyltransferase
dob: doppelganger von brummer
DPE: days post-eclosion
dsx: doublesex
EcR: ecdysone receptor
elav: embryonic lethal abnormal vision
foxo: forkhead box O
fru: fruitless
GFP: green fluorescent protein
GPAT: glycerol-3-phosphate acyltransferase
HNF4: hepatocyte nuclear factor 4
hsl: hormone-sensitive lipase
IIS: insulin/insulin-like growth factor signaling
IPC: insulin-producing cell
Klar: Klarsicht
LD-GFP: lipid droplet–targeted GFP
Lpin: Lipin
lsd-1: lipid storage droplet-1
lsd-2: lipid storage droplet-2
mdy: midway
mino: minotaur
nSyb: neuronal Synaptobrevin
PDF: pigment dispersing factor
PGC-1: peroxisome proliferator–activated receptor γ coactivator 1
PLIN: perilipin
qPCR: quantitative real-time PCR
ROS: reactive oxygen species
RQ: respiratory quotient
sNPF: short neuropeptide F
SREBP: sterol response element binding protein
srl: spargel
tj: traffic jam
UAS: upstream activation sequence
w: white
