# Supplementary figures and images for: A role for triglyceride lipase brummer in the regulation of sex differences in Drosophila fat storage and breakdown
Source: PLoS Biol. 2020 Jan 21;18(1):e3000595. doi: 10.1371/journal.pbio.3000595 (PMC6994176; doi:10.1371/journal.pbio.3000595)

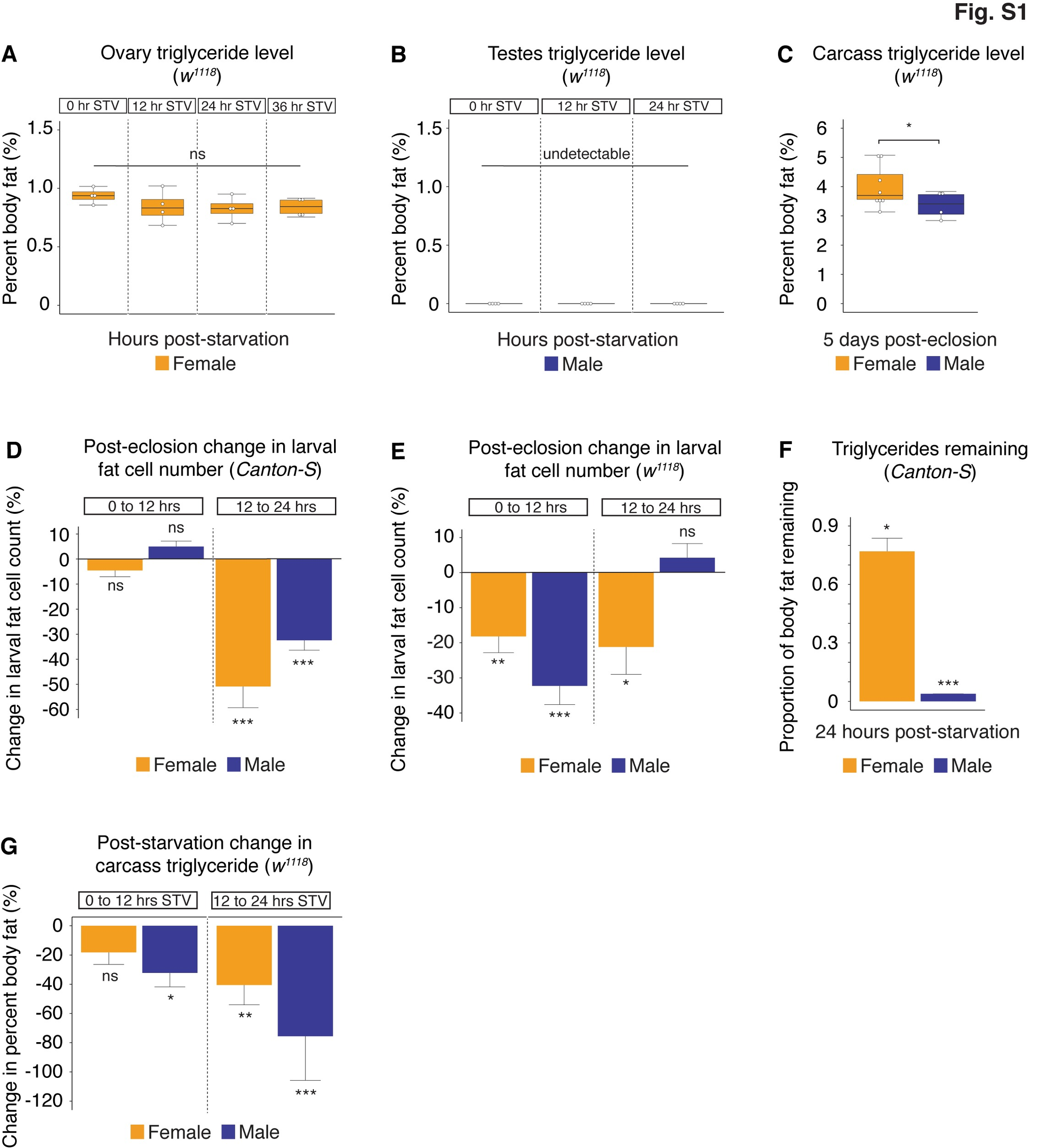

Supplement: S1 Fig — (A) Ovary triglyceride levels were not significantly different between fed virgin w1118 females and starved virgin w1118 females at all time points STV (p = 0.56, 0.44, 0.55, respectively; one-way ANOVA followed by Tukey HSD test). (B) The amount of triglyceride contained in the testes of 5-day-old virgin w1118 males is below the limit of detection for the coupled colorimetric assay; therefore, statistics could not be performed. (C) Triglyceride levels in 5-day-old virgin w1118 female carcasses devoid of ovaries were significantly higher than in age-matched male carcasses devoid of testes (p = 0.022; Student t test). (D) Larval fat cells in newly eclosed CS virgin females and males showed no significant change between 0 and 12 hours post-eclosion (p = 0.53 and 0.43 for females and males, respectively; one-way ANOVA followed by Tukey HSD test), but there was a significant decrease in the larval fat cell number in both sexes between 12 and 24 hours post-eclosion (p = 0.0 and 0.0 for females and males, respectively; one-way ANOVA followed by Tukey HSD test). (E) The number of larval fat cells significantly decreased in w1118 virgin females and males between 0 and 12 hours post-eclosion (p = 0.0071 and 1.0 × 10^−7^ for females and males, respectively; one-way ANOVA followed by Tukey HSD test) and decreased further in females but not males between 12 and 24 hours post-eclosion (p = 0.011 and 0.8 for females and males, respectively; one-way ANOVA followed by Tukey HSD test). (F) After 24 hours of starvation, triglyceride levels in virgin CS females remain at 77% of the triglyceride level in a fed virgin female (p = 0.034; one-way ANOVA followed by Tukey HSD test), whereas virgin CS males have only 4% of the triglyceride level in a fed male remaining (p = 6 × 10^−7^; one-way ANOVA followed by Tukey HSD test). (G) In 5-day-old virgin female w1118 carcasses devoid of ovaries, there was no significant decrease in triglyceride levels between 0 and 12 hours STV, whereas there was a [file pbio.3000595.s001.tif]

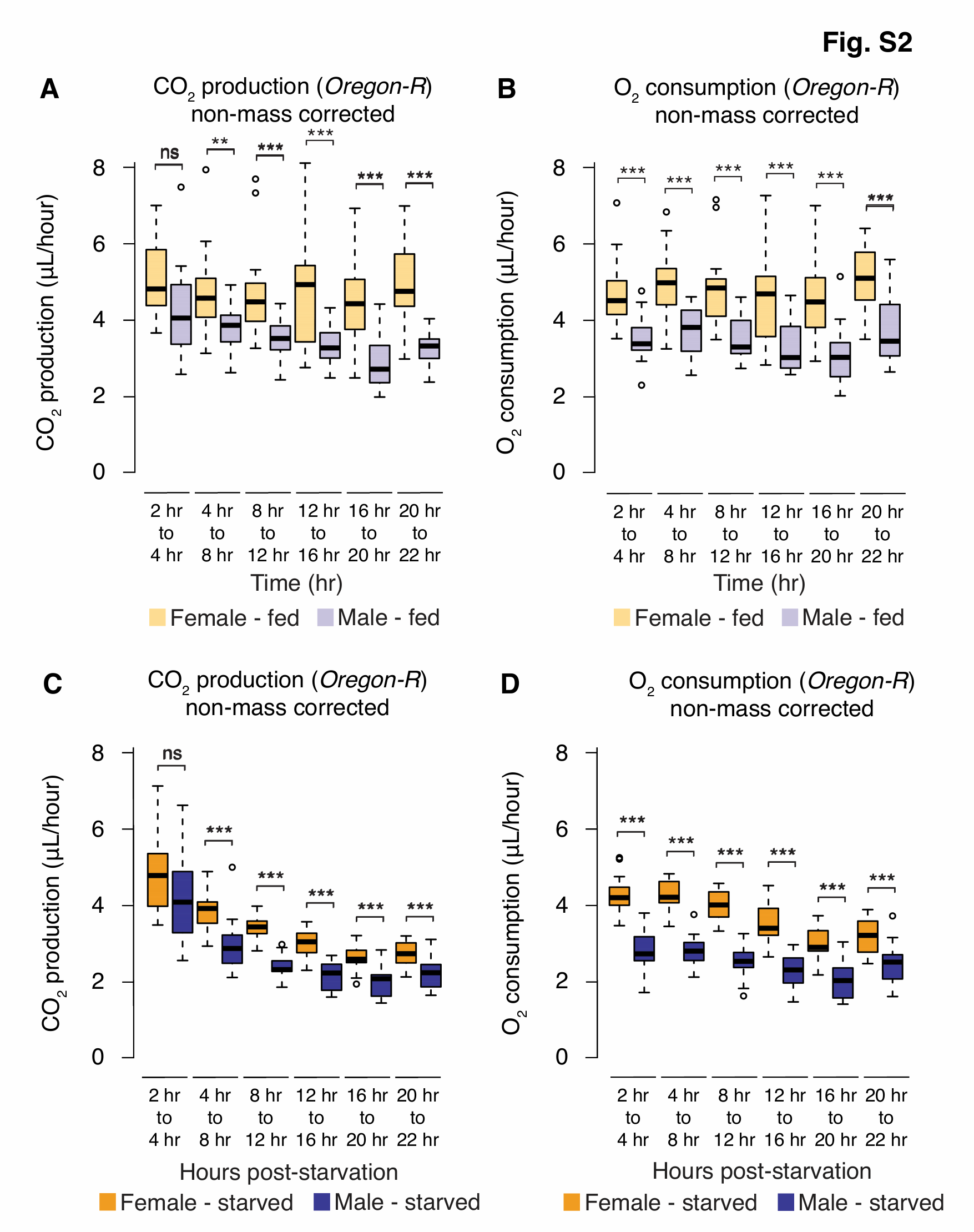

Supplement: S2 Fig — (A) Non-mass-corrected CO2 production was significantly higher in Oregon-R fed females compared with fed males for the majority of the intervals during the 24-hour observation period (p = 0.067, 0.0031, 2.4 × 10^−4^, 4.5 × 10^−4^, 1.4 × 10^−5^, 1.7 × 10^−7^, respectively; Student t test at each time interval). (B) Non-mass-corrected O2 consumption was significantly higher in fed females compared with fed males at all intervals during the observation period (p = 1.5 × 10^−5^, 1.6 × 10^−5^, 6.0 × 10^−6^, 5.8 × 10^−5^, 1.8 × 10^−5^, 3.8 × 10^−5^, respectively; Student t test at each time interval). (C) Non-mass-corrected CO2 production was significantly higher in starved females at every interval post-starvation from 4 hours onward (p = 0.44, 1.3 × 10^−5^, 5.9 × 10^−13^, 2.4 × 10^−9^, 1.9 × 10^−7^, 1.5 × 10^−4^, respectively; Student t test at each time interval). (D) Non-mass-corrected O2 consumption was significantly higher in starved females compared with starved males at all time intervals post-starvation (p = 6.0 × 10^−12^, 1.1 × 10^−15^, 1.2 × 10^−14^, 4.3 × 10^−10^, 1.7 × 10^−8^, 2.5 × 10^−5^, respectively; Student t test at each time interval). For indirect calorimetry measurements, the p-values are listed in the following order: difference between the sexes at 2–4 hours, 4–8 hours, 8–12 hours, 12–16 hours, 16–20 hours, and 20–22 hours. Asterisks indicate a significant difference between two sexes, two genotypes, or two time points (*p < 0.05, **p < 0.01, ***p < 0.001). Error bars on graphs represent SEM. Quantitative measurements underlying all graphs are available in S2 Data. ns, no significant difference between two sexes, two genotypes, or time points. (TIF) [file pbio.3000595.s002.tif]

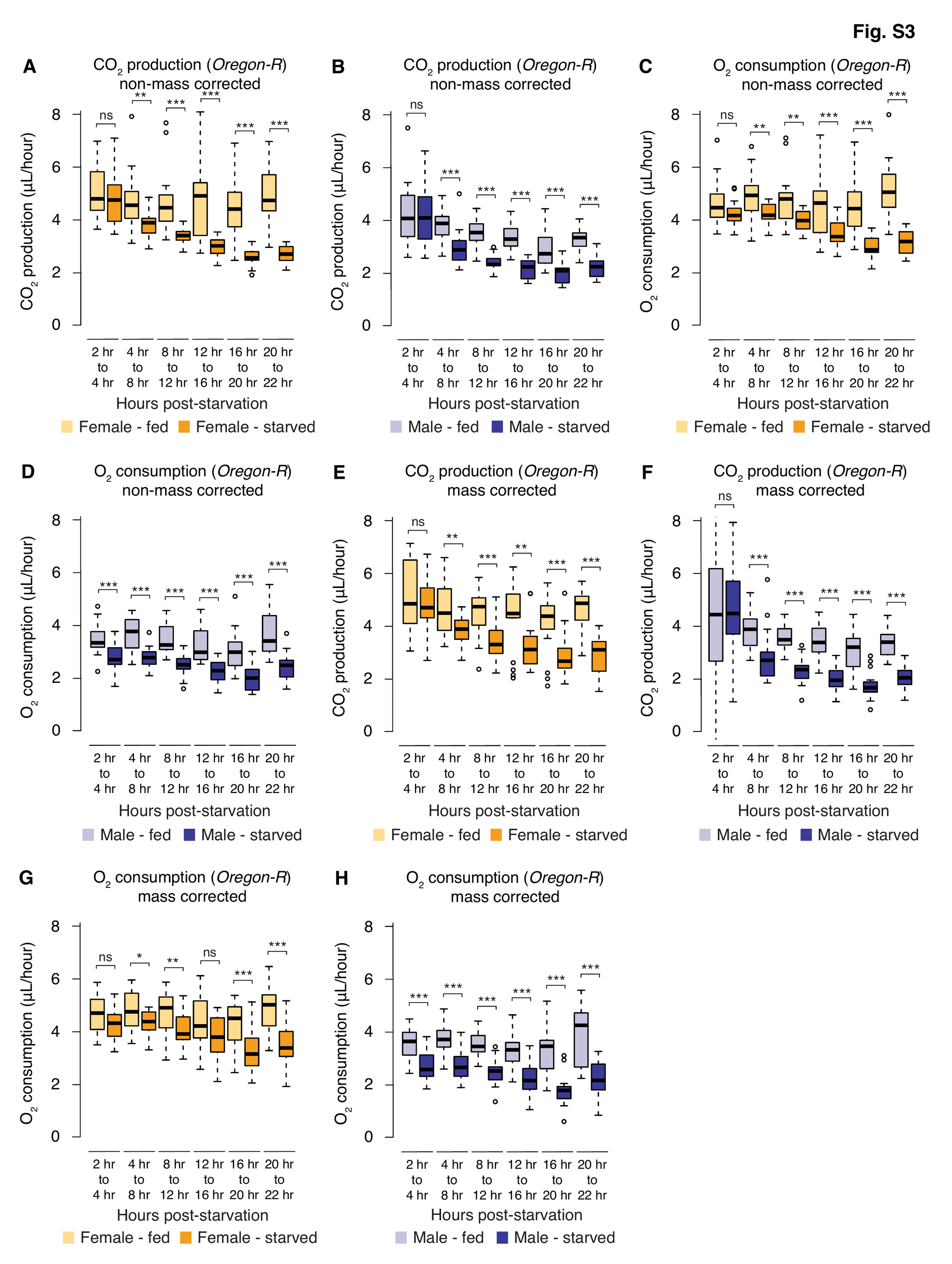

Supplement: S3 Fig — (A) Non-mass-corrected CO2 production was significantly higher in Oregon-R fed females compared with starved females for most intervals post-starvation during the observation period (p = 0.20, 0.0024, 4.9 × 10^−5^, 5.2 × 10^−5^, 1.4 × 10^−6^, 1.6 × 10^−9^, respectively; Student t test at each time interval). (B) Non-mass-corrected CO2 production was significantly higher in Oregon-R fed males compared with starved males for most intervals post-starvation during the observation period (p = 0.99, 6.1 × 10^−5^, 4.7 × 10^−10^, 2.2 × 10^−9^, 1.3 × 10^−5^, 4.1 × 10^−9^, respectively; Student t test at each time interval). (C) Non-mass-corrected O2 consumption was significantly higher in fed females compared with starved females for most intervals post-starvation during the observation period (p = 0.072, 0.0080, 0.0013, 8.1 × 10^−4^, 7.7 × 10^−6^, 6.6 × 10^−8^, respectively; Student t test at each time interval). (D) Non-mass-corrected O2 consumption was significantly higher in fed males compared with starved males at all intervals post-starvation (p = 1.6 × 10^−4^, 9.6 × 10^−6^, 1.5 × 10^−7^, 3.6 × 10^−6^, 9.3 × 10^−6^, 4.8 × 10^−6^, respectively; Student t test at each time interval). (E) Mass-corrected CO2 production was significantly higher in fed females compared with starved females for most intervals post-starvation during the observation period (p = 0.55, 0.0026, 4.9 × 10^−5^, 0.0016, 1.3 × 10^−4^, 8.1 × 10^−9^, respectively; Student t test at each time interval). (F) Mass-corrected CO2 production was significantly higher in fed males compared with starved males for most intervals post-starvation during the observation period (p = 0.59, 4.4 × 10^−4^, 7.5 × 10^−10^, 2.0 × 10^−9^, 7.0 × 10^−7^, 3.0 × 10^−10^, respectively; Student t test at each time interval). (G) Mass-corrected O2 consumption was significantly higher in fed females compared with starved females for most intervals post-starvation during the observation period (p = 0.053, 0.014, 0.0098, 0.063, 7.6 × 10^−4^, 6.2 × 10^−6^, respectively; Student t test at eac [file pbio.3000595.s003.tif]

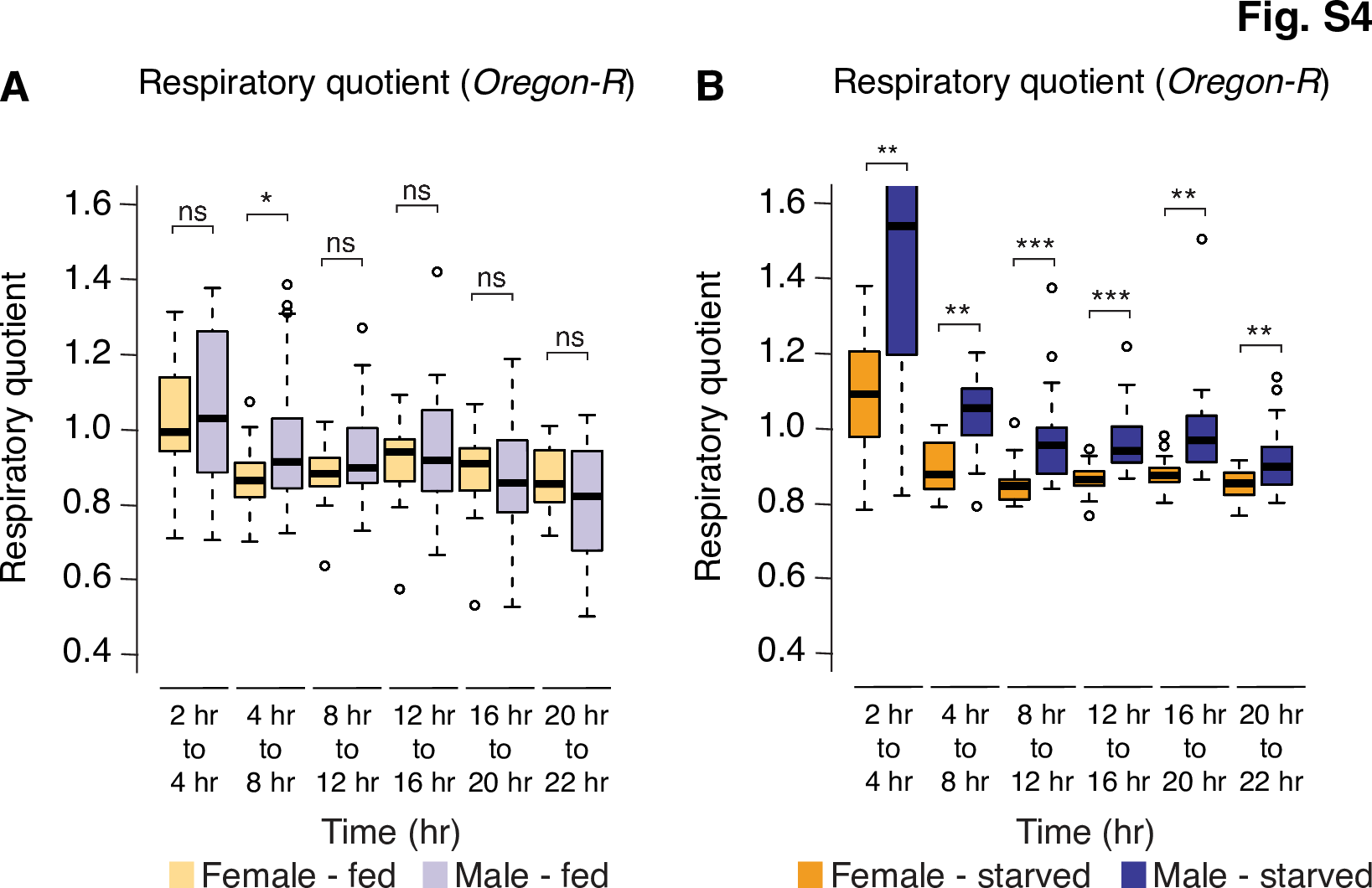

Supplement: S4 Fig — (A) In fed Oregon-R females and males, we observed no significant differences in the RQ throughout most of the observation period, with the exception of the 4- to 8-hour interval (p = 0.17, 0.031, 0.13, 0.43, 0.58, 0.15, respectively; Student t test at each time interval). (B) In starved Oregon-R females and males, starved males have a significantly higher RQ at all time intervals post-starvation (p = 0.0012, 0.0013, 7.7 × 10^−4^, 6.6 × 10^−5^, 0.0013, 0.0032, respectively; Student t test at each time interval). For indirect calorimetry measurements, the p-values are listed in the following order: difference between the sexes at 2–4 hours, 4–8 hours, 8–12 hours, 12–16 hours, 16–20 hours, and 20–22 hours. Asterisks indicate a significant difference between two sexes, two genotypes, or two time points (*p < 0.05, **p < 0.01, ***p < 0.001). Error bars on graphs represent SEM. Quantitative measurements underlying all graphs are available in S2 Data. ns, no significant difference between two sexes, two genotypes, or time points; RQ, respiratory quotient. (TIF) [file pbio.3000595.s004.tif]

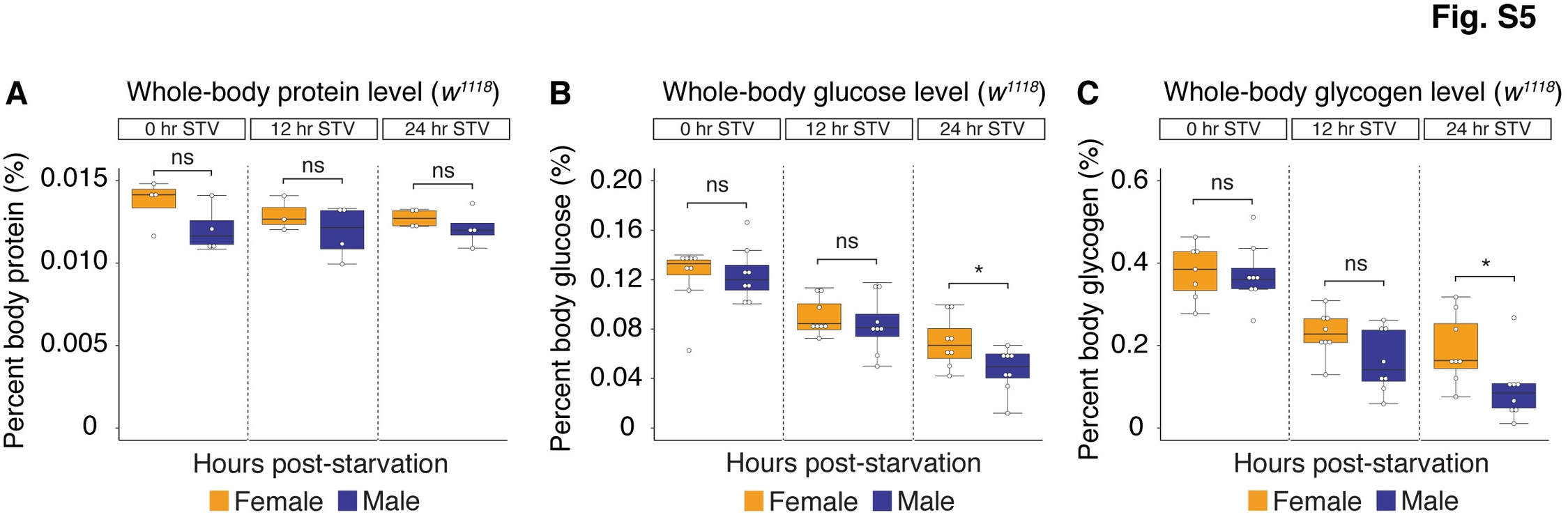

Supplement: S5 Fig — (A) Whole-body protein levels were not significantly different between 5-day-old virgin w1118 males and females at any time point STV (p = 0.16, 0.19, 0.37, respectively; Student t test at each time point). (B) Whole-body glucose levels were not significantly different between the sexes at 0 and 12 hours STV but were significantly higher in females compared with males by 24 hours STV (p = 0.87, 0.48, 0.034, respectively; Student t test at each time point). (C) Whole-body glycogen levels were not significantly different between the sexes at 0 or 12 hours STV but were significantly higher in females compared with males by 24 hours STV (p = 0.86, 0.063, 0.033, respectively; Student t test at each time point). The p-values are listed in the following order: difference between females and males at 0 hours, 12 hours, and 24 hours STV. Asterisks indicate a significant difference between two sexes, two genotypes, or two time points (*p < 0.05; **p < 0.01, ***p < 0.001). Error bars on graphs represent SEM. Quantitative measurements underlying all graphs are available in S1 Data. ns, no significant difference between two sexes, two genotypes, or time points; STV, post-starvation; w, white. (TIF) [file pbio.3000595.s005.tif]

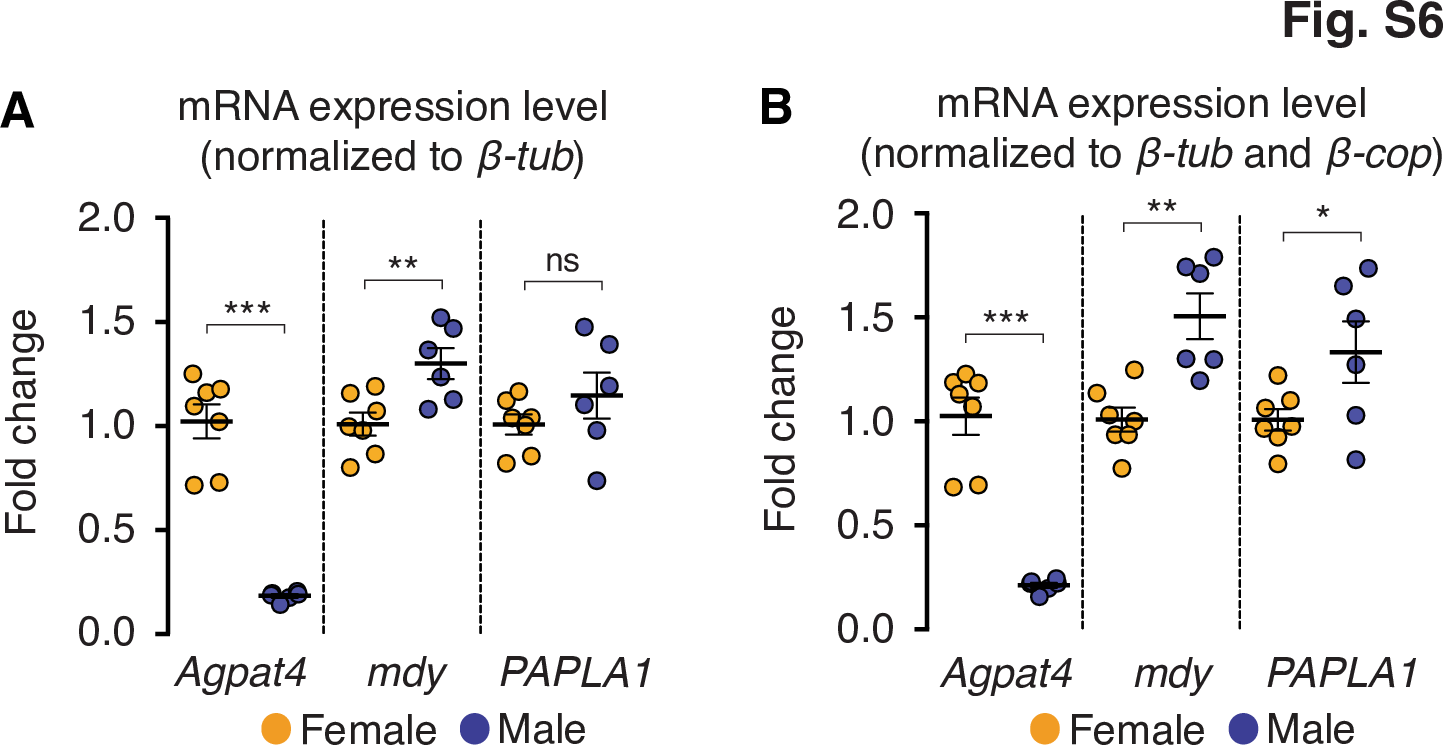

Supplement: S6 Fig — (A) In normal culture conditions, Agpat4 is female-biased, mdy is male-biased, and PAPLA1 is not sex biasedly expressed when normalized to β-tubulin (p = <0.0001, 0.0079, and 0.25, respectively; Student t test for each gene). (B) In normal culture conditions, Agpat4 is female biased and mdy and PAPLA1 are male biased when normalized to both β-tubulin and β-cop (p = <0.0001, 0.0015, and 0.048, respectively; Student t test for each gene). Asterisks indicate a significant difference between two sexes, two genotypes, or two time points (*p < 0.05; **p < 0.01, ***p < 0.001). Error bars on graphs represent SEM. Quantitative measurements underlying all graphs are available in S3 Data. β-cop, Coat Protein (coatomer) β; Agpat, 1-acylglycerol-3-phosphate O-acyltransferase; ns, no significant difference between two sexes, two genotypes, or time points; mdy, midway; PAPLA1, phosphatidic acid phospholipase A1. (TIF) [file pbio.3000595.s006.tif]

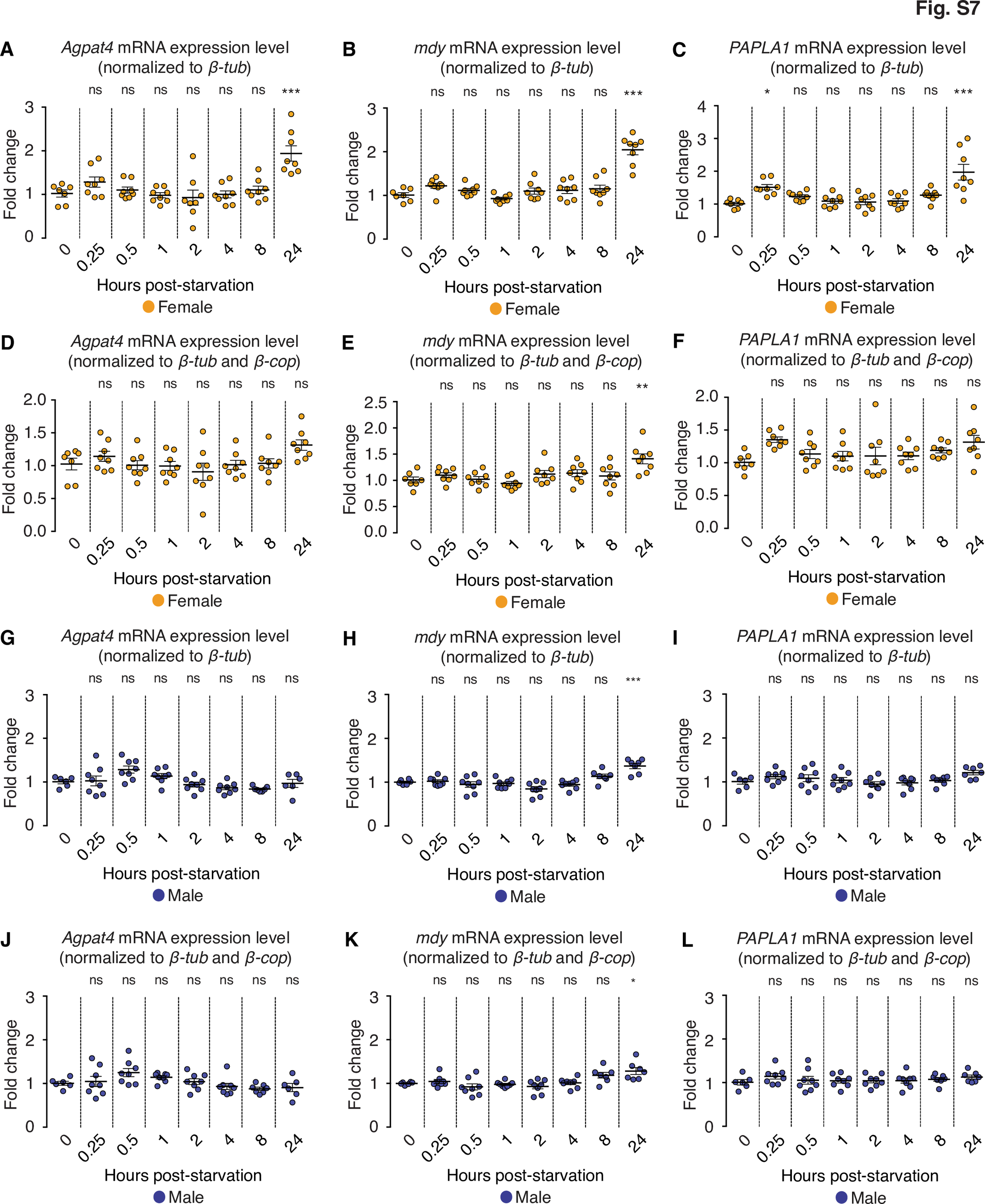

Supplement: S7 Fig — (A–C) In starvation conditions, Agpat4, mdy, and PAPLA1 female gene expression is significantly increased at 24 hours post-starvation when normalized to β-tubulin (p = <0.0001, <0.0001, and <0.0001, respectively at 24 hours post-starvation; one-way ANOVA followed by Tukey HSD test for each gene). (D–F) In starvation conditions, Agpat4 and PAPLA1 female gene expression is not significantly increased at 24 hours post-starvation, whereas mdy is significantly increased at 24 hours post-starvation when normalized to β-tubulin and β-cop (p = >0.05, >0.05, and <0.01 respectively at 24 hours post-starvation; one-way ANOVA followed by Tukey HSD test for each gene). (G–I) In starvation conditions, Agpat4 and PAPLA1 male gene expression is not significantly increased at 24 hours post-starvation, whereas mdy is significantly increased at 24 hours post-starvation when normalized to β-tubulin (p = >0.05, >0.05, and <0.001, respectively, at 24 hours post-starvation; one-way ANOVA followed by Tukey HSD test for each gene). (J–L) In starvation conditions, Agpat4 and PAPLA1 male gene expression is not significantly increased at 24 hours post-starvation, whereas mdy is significantly increased at 24 hours post-starvation when normalized to β-tubulin and β-cop (p = >0.05, >0.05, and <0.05, respectively, at 24 hours post-starvation; one-way ANOVA followed by Tukey HSD test for each gene). Asterisks indicate a significant difference between two sexes, two genotypes, or two time points (*p < 0.05, **p < 0.01, ***p < 0.001). Error bars on graphs represent SEM. See S1 Table for list of all comparisons and p-values; quantitative measurements underlying all graphs are available in S3 Data. β-cop, Coat Protein (coatomer) β; Agpat, 1-acylglycerol-3-phosphate O-acyltransferase; HSD, honest significant difference; mdy, midway; ns, no significant difference between two sexes, two genotypes, or time points; PAPLA1, phosphatidic acid phospholipase A1. (TIF) [file pbio.3000595.s007.tif]

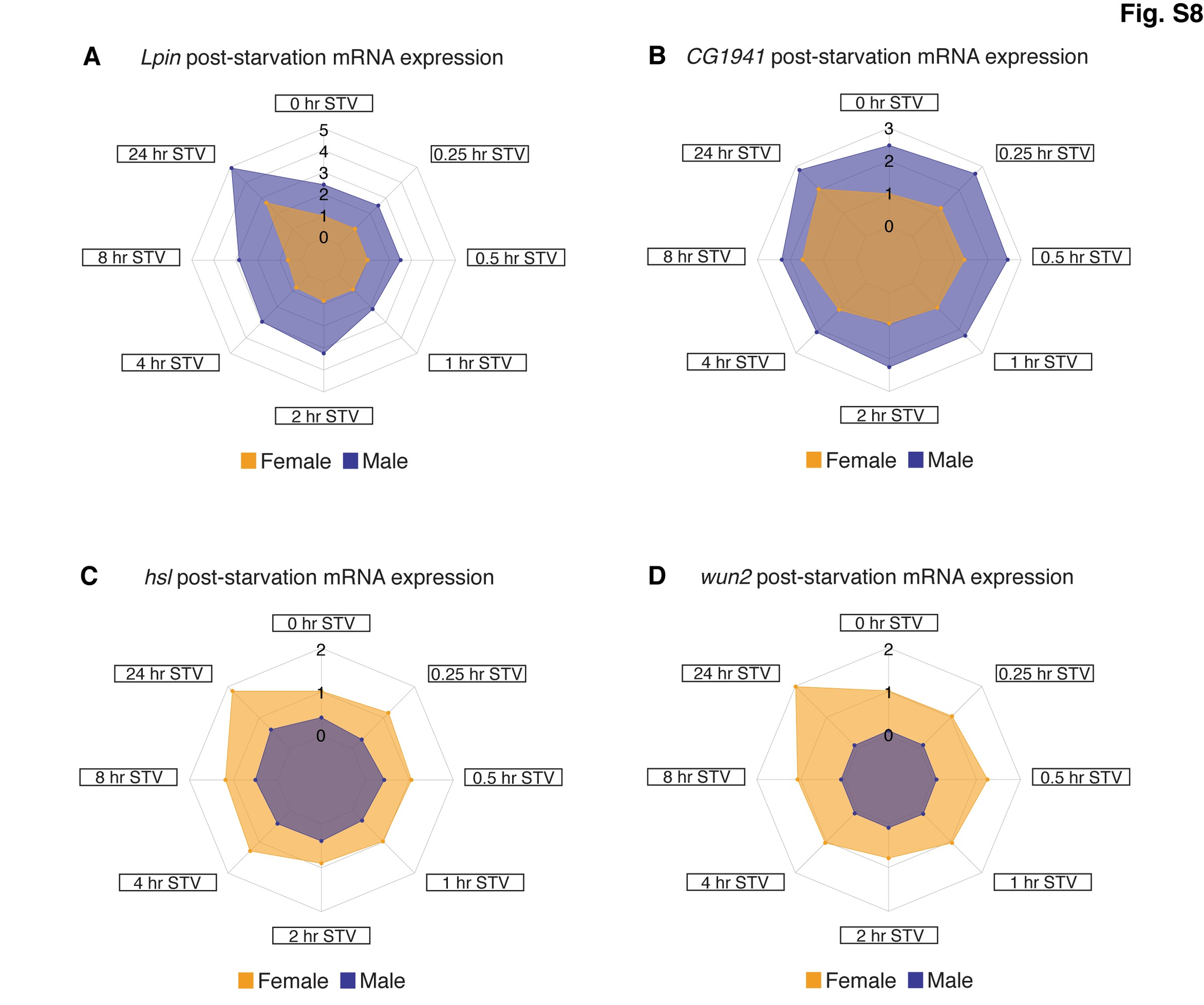

Supplement: S8 Fig — Radar plots demonstrating gene expression in males and females throughout the starvation period for representative genes. (A, B) mRNA levels of two genes with male-biased expression throughout the starvation period in both males and females (Lpin, CG1941). (C, D) mRNA levels of two genes with female-biased expression throughout the starvation period in both males and females (hsl, wun2). Sex-biased expression of these genes remains consistent throughout the starvation period. See S1 Table for list of all comparisons and p-values; quantitative measurements underlying all graphs are available in S3 Data. hsl, hormone-sensitive lipase; Lpin, Lipin; STV, post-starvation; wun2, wunen-2. (TIF) [file pbio.3000595.s008.tif]

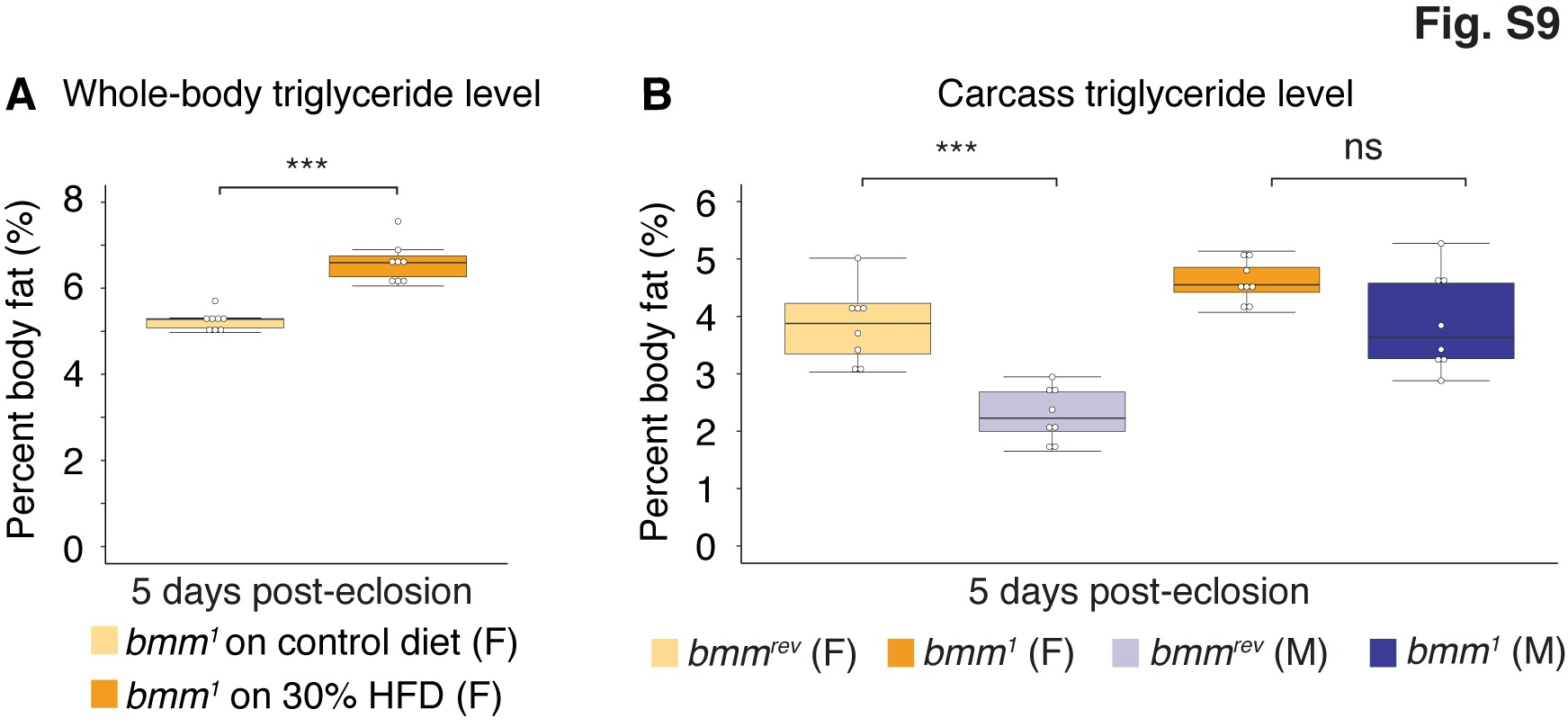

Supplement: S9 Fig — (A) Triglyceride levels in 5-day-old bmm1 mutant females fed a high fat diet (HFD) were significantly higher than in bmm1 mutant females fed standard fly food (p = 3.9 × 10^−6^; Student t test). (B) Triglyceride storage was significantly higher in 5-day-old virgin bmmrev female carcasses lacking ovaries compared with age-matched bmmrev males, whereas there was no significant difference in whole-body triglyceride levels in 5-day-old bmm1 mutant virgin female carcasses devoid of ovaries compared with age-matched bmm1 mutant virgin males (p = 0.00013 in bmmrev animals, 0.12 in bmm1 mutants; one-way ANOVA followed by Tukey HSD test). Asterisks indicate a significant difference between two sexes, two genotypes, or two time points (*p < 0.05, **p < 0.01, ***p < 0.001). Error bars on graphs represent SEM. See S1 Table for list of all multiple comparisons and p-values; quantitative measurements underlying all graphs are available in S1 Data. bmm, brummer; F, female; HFD, high-fat diet; HSD, honest significant difference; M, male; ns, no significant difference between two sexes, two genotypes, or time points. (TIF) [file pbio.3000595.s009.tif]

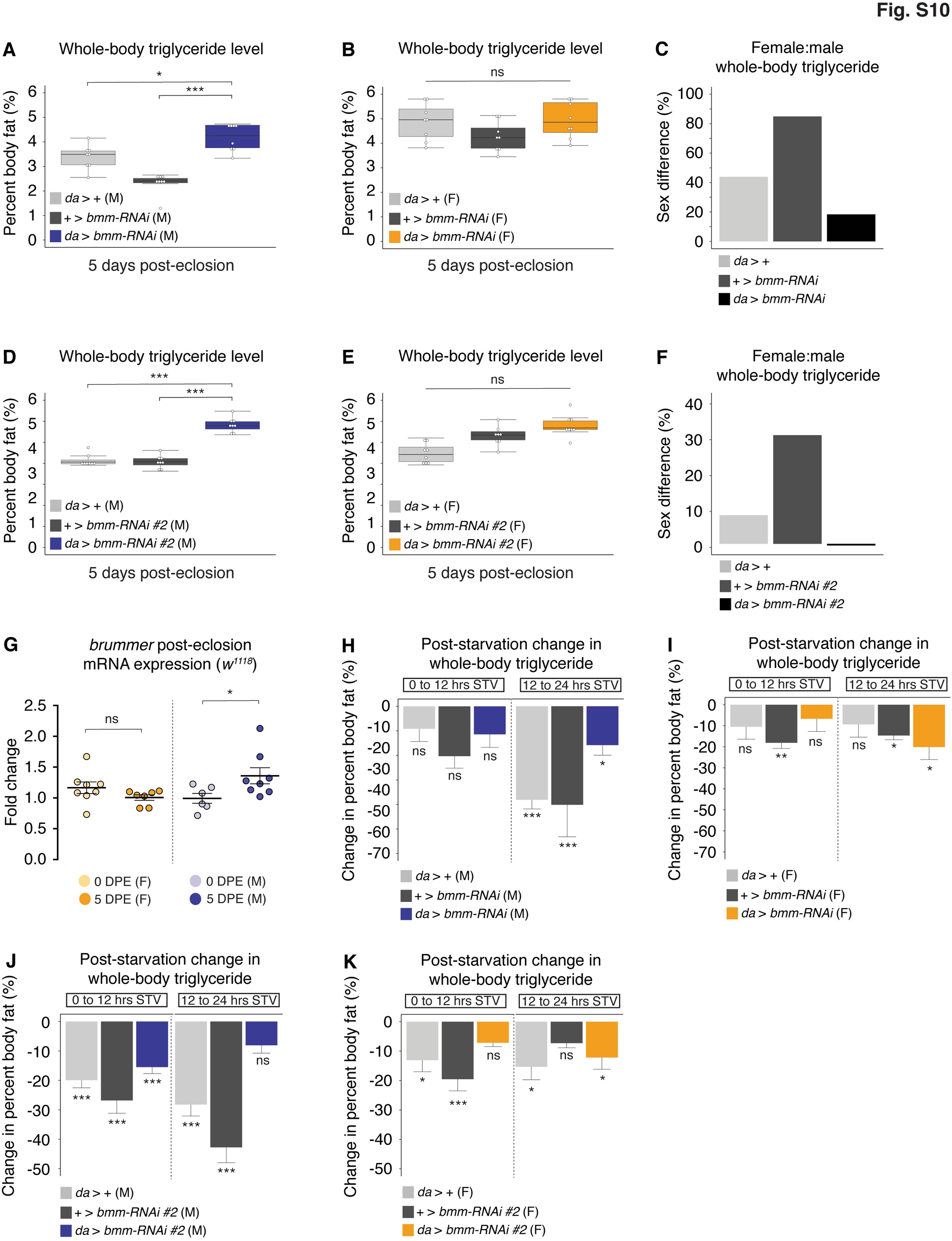

Supplement: S10 Fig — (A) In 5-day-old virgin da>UAS-bmm-RNAi males, triglyceride levels were significantly higher than in da>+ or +>UAS-bmm-RNAi control males (p = 0.012 and 6.0 × 10^−7^, respectively; one-way ANOVA followed by Tukey HSD test). (B) Triglyceride levels in 5-day-old virgin da>UAS-bmm-RNAi females were not significantly different from da>+ or +>UAS-bmm-RNAi control females (p = 0.98 and 0.16, respectively; one-way ANOVA followed by Tukey HSD test). (C) The male-biased effects of da>UAS-bmm-RNAi on triglyceride storage reduced the sexual dimorphism in triglyceride storage compared with da>+ or +>UAS-bmm-RNAi controls. (D) In 5-day-old virgin da>UAS-bmm-RNAi#2 (BDSC #25926) males, triglyceride levels were significantly higher than in da>+ or +>UAS-bmm-RNAi#2 control males (p = 0.0 and 0.0, respectively; one-way ANOVA followed by Tukey HSD test). (E) Triglyceride levels in 5-day-old virgin da>UAS-bmm-RNAi#2 (BDSC #25926) females were not significantly different from da>+ or +>UAS-bmm-RNAi#2 control females (p = 5.8 × 10^−5^ and 0.14, respectively; one-way ANOVA followed by Tukey HSD test). (F) The male-biased effects of da>UAS-bmm-RNAi#2 (BDSC #25926) on triglyceride storage reduced the sexual dimorphism in triglyceride storage compared with da>+ or +>UAS-bmm-RNAi#2 controls. (G) Between 0 DPE and 5 DPE, mRNA expression levels for bmm were not significantly increased in virgin w1118 females but were significantly increased in virgin w1118 males (p = 0.17 and 0.048, respectively; Student t test). (H) Between 0 and 12 hours STV, we observed no triglyceride breakdown in da>+, +>UAS-bmm-RNAi, or da>UAS-bmm-RNAi males (p = 0.3, 0.053, and 0.18, respectively; one-way ANOVA followed by Tukey HSD test); however, between 12 and 24 hours STV, the magnitude of triglyceride breakdown in da>UAS-bmm-RNAi males was lower than da>+ and +>UAS-bmm-RNAi control males (p = 0.038, 3.1 × 10^−6^, and 0.00046, respectively; one-way ANOVA followed by Tukey HSD test). (I) Between 0 and 12 hours STV, and bet [file pbio.3000595.s010.tif]

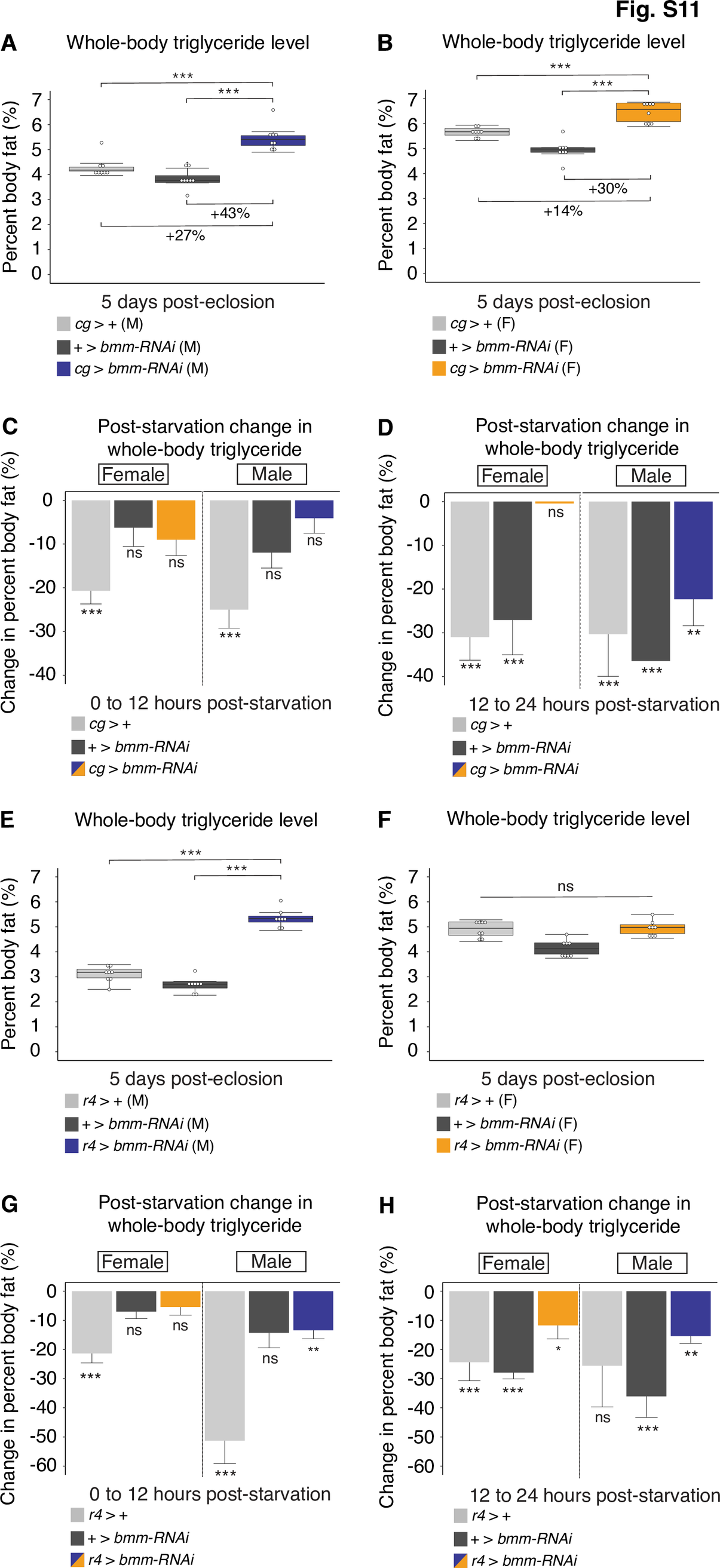

Supplement: S11 Fig — (A) Whole-body triglyceride storage in 5-day-old virgin males overexpressing UAS-bmm-RNAi in the fat body (cg>UAS-bmm-RNAi) was significantly higher than age-matched control males (cg>+ and +>UAS-bmm-RNAi) (p = 1.0 × 10^−4^ and 8.0 × 10^−7^, respectively; one-way ANOVA followed by Tukey HSD test). (B) Whole-body triglyceride storage in 5-day-old virgin females overexpressing UAS-bmm-RNAi in the fat body (cg>UAS-bmm-RNAi) was significantly higher than age-matched control females (cg>+ and +>UAS-bmm-RNAi) (p = 5.5 × 10^−4^ and 1.0 × 10^−7^, respectively; one-way ANOVA followed by Tukey HSD test). (C) There was a significant reduction in control female and male triglyceride levels (cg>+) between 0 and 12 hours post-starvation (p = 2.8 × 10^−6^ and 1.0 × 10^−4^, respectively; one-way ANOVA followed by Tukey HSD test); however, we observed no significant reduction in whole-body triglyceride levels in 5-day-old virgin cg>UAS-bmm-RNAi females and males between 0 and 12 hours post-starvation (p = 0.54 and 0.92, respectively; one-way ANOVA followed by Tukey HSD test). (D) There was a significant reduction in triglyceride storage in control females and males (cg>+ and +>UAS-bmm-RNAi) between 12 and 24 hours post-starvation (p = 1 × 10^−7^ and 2.7 × 10^−4^ (females) and p = 4.0 × 10^−4^ and 2 × 10^−7^ (males), respectively; one-way ANOVA followed by Tukey HSD test); however, there was no significant change in whole-body triglyceride levels in 5-day-old virgin cg>UAS-bmm-RNAi females between 12 and 24 hours post-starvation (p = 1.0; one-way ANOVA followed by Tukey HSD test). In males, there was a significant but blunted decrease in triglyceride levels in 5-day-old cg>UAS-bmm-RNAi virgin males between 12 and 24 hours post-starvation (p = 0.0011; one-way ANOVA followed by Tukey HSD). (E) Whole-body triglyceride storage in 5-day-old r4>UAS-bmm-RNAi males was significantly higher than r4>+ and +>UAS-bmm-RNAi control males (p = 0.0 and 0.0, respectively; one-way ANOVA followed by Tukey HSD test). (F) r [file pbio.3000595.s011.tif]

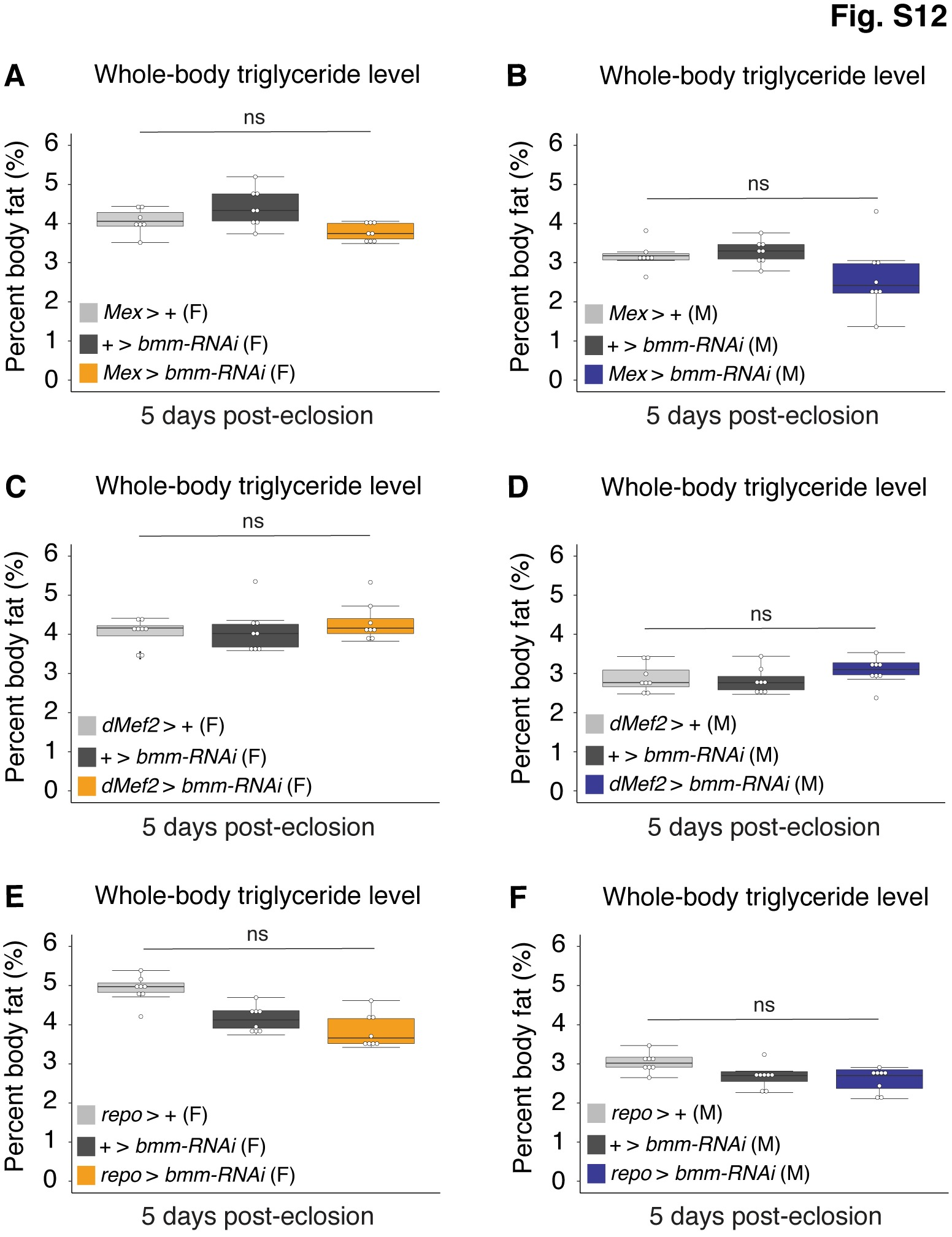

Supplement: S12 Fig — (A) Whole-body triglyceride storage in 5-day-old virgin females overexpressing UAS-bmm-RNAi in the gut (Mex>UAS-bmm-RNAi) was not significantly different from age-matched control females (Mex>+ and +>UAS-bmm-RNAi) (p = 0.31 and 0.0073, respectively; one-way ANOVA followed by Tukey HSD test). (B) Whole-body triglyceride storage in 5-day-old virgin males overexpressing UAS-bmm-RNAi in the gut (Mex>UAS-bmm-RNAi) was not significantly different from age-matched control males (Mex>+ and +>UAS-bmm-RNAi) (p = 0.17 and 0.079, respectively; one-way ANOVA followed by Tukey HSD test). (C) Whole-body triglyceride storage in 5-day-old virgin females overexpressing UAS-bmm-RNAi in the muscle (dMef2>UAS-bmm-RNAi) was not significantly different from age-matched control females (dMef2>+ and +>UAS-bmm-RNAi) (p = 0.50 and 0.70, respectively; one-way ANOVA followed by Tukey HSD test). (D) Whole-body triglyceride storage in 5-day-old virgin males overexpressing UAS-bmm-RNAi in the muscle (dMef2>UAS-bmm-RNAi) was not significantly different from age-matched control males (dMef2>+ and +>UAS-bmm-RNAi) (p = 0.54 and 0.34, respectively; one-way ANOVA followed by Tukey HSD test). (E) Whole-body triglyceride level in 5-day-old virgin females overexpressing UAS-bmm-RNAi in the glia (repo>UAS-bmm-RNAi) was not significantly different from age-matched control females (repo>+ and +>UAS-bmm-RNAi) (p = 3.2 × 10^−5^ and 0.26, respectively; one-way ANOVA followed by Tukey HSD test). (F) Whole-body triglyceride levels in 5-day-old virgin males overexpressing UAS-bmm-RNAi in the glia (repo>UAS-bmm-RNAi) were not significantly different from age-matched control males (repo>+ and +>UAS-bmm-RNAi) (p = 0.016 and 0.8, respectively; one-way ANOVA followed by Tukey HSD test). The p-values are listed in the following order: difference between the GAL4/UAS genotype and the GAL4 control and difference between the GAL4/UAS genotype and the UAS control, respectively. Asterisks indicate a significant difference betwe [file pbio.3000595.s012.tif]

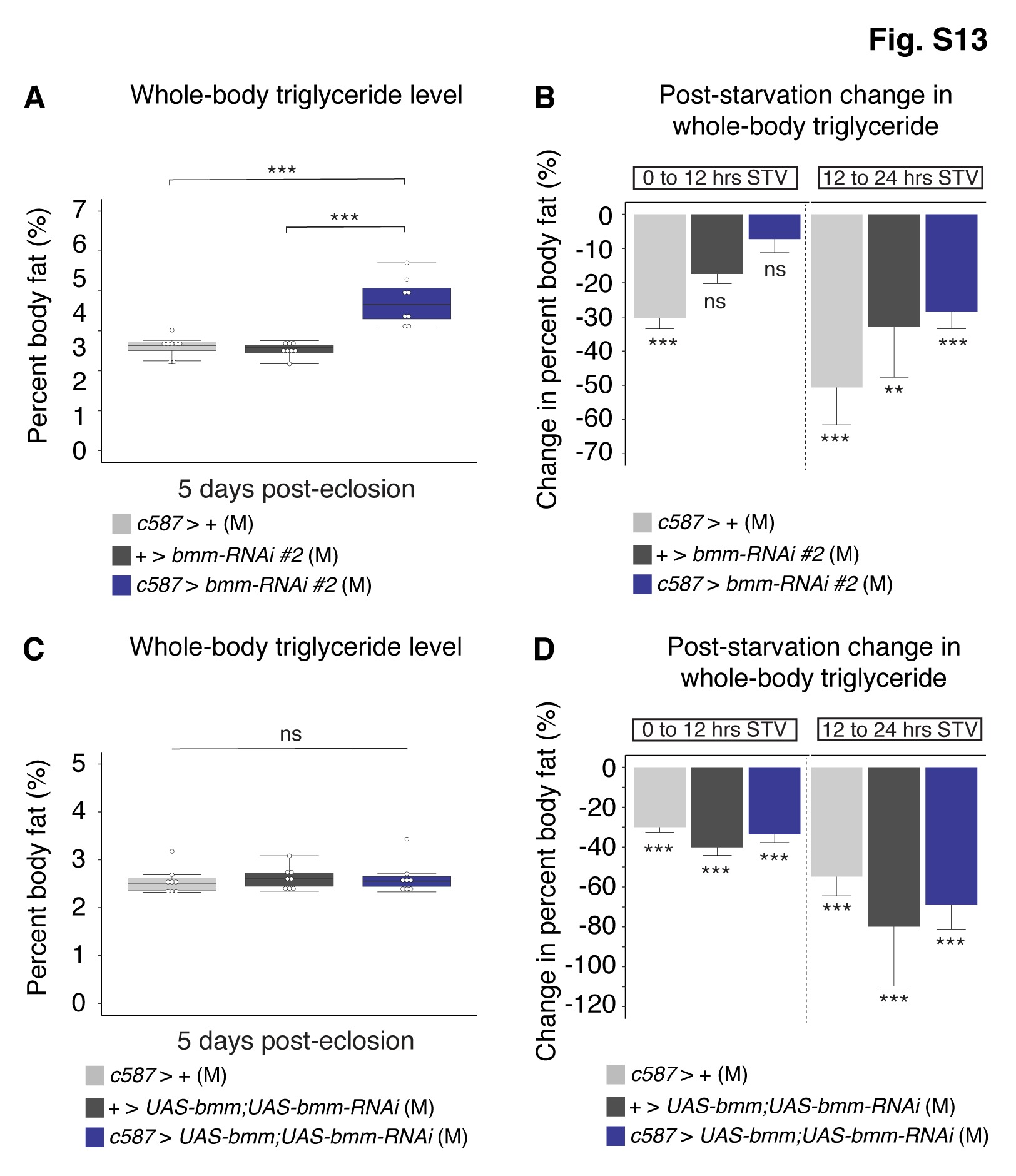

Supplement: S13 Fig — (A) Whole-body triglyceride storage in males with c587-GAL4-mediated overexpression of an additional UAS-bmm-RNAi#2 (VDRC #37877) transgene in the somatic cells of the male gonad was significantly higher than in control males (c587-GAL4>+ and +>UAS-bmm-RNAi#2) (p = 2.3 × 10^−5^ and 1.1 × 10^−5^, respectively; one-way ANOVA followed by Tukey HSD test). (B) The decrease in whole-body triglyceride levels in c587-GAL4>UAS-bmm-RNAi#2 (VDRC #37877) males was blunted compared with c587-GAL4>+ and +>UAS-bmm-RNAi#2 control males between 0 and 12 hours STV (p = 0.47, 5.8 × 10^−6^, and 0.065, respectively; one-way ANOVA followed by Tukey HSD test) but not between 12 and 24 hours STV (p = 8.2 × 10^−4^, 6.0 × 10^−7^, and 0.0033, respectively; one-way ANOVA followed by Tukey HSD test). (C) Whole-body triglyceride levels in 5-day-old virgin c587>UAS-bmm;UAS-bmm-RNAi males were not significantly different to c587-GAL4>+ and +>UAS-bmm;UAS-bmm-RNAi controls, demonstrating that re-expression of UAS-bmm rescued the increased fat storage caused by loss of bmm in the somatic cells of the male gonad (p = 0.87 and 1.0, respectively; one-way ANOVA followed by Tukey HSD test). (D) Triglyceride breakdown post-starvation among 5-day-old virgin c587>UAS-bmm;UAS-bmm-RNAi males and control males (c587>+ and +>UAS-bmm;UAS-bmm-RNAi) was decreased by a similar magnitude between both 0 and 12 hours or 12 and 24 hours STV, demonstrating that re-expression of UAS-bmm rescued the effects of bmm loss in the somatic cells of the male gonad post-starvation (p = 2.2 × 10^−6^, 1.7 × 10^−6^, and 0.0 for 0–12 hours and 0.0, 0.0, and 0.0 for 12–24 hours, respectively; one-way ANOVA followed by Tukey HSD test). Asterisks indicate a significant difference between two sexes, two genotypes, or two time points (*p < 0.05, **p < 0.01, ***p < 0.001). Error bars on graphs depicting percent body fat represent SEM; error bars on graphs depicting the change in percent body fat represent COE. See S1 Table for a list of all multiple comp [file pbio.3000595.s013.tif]

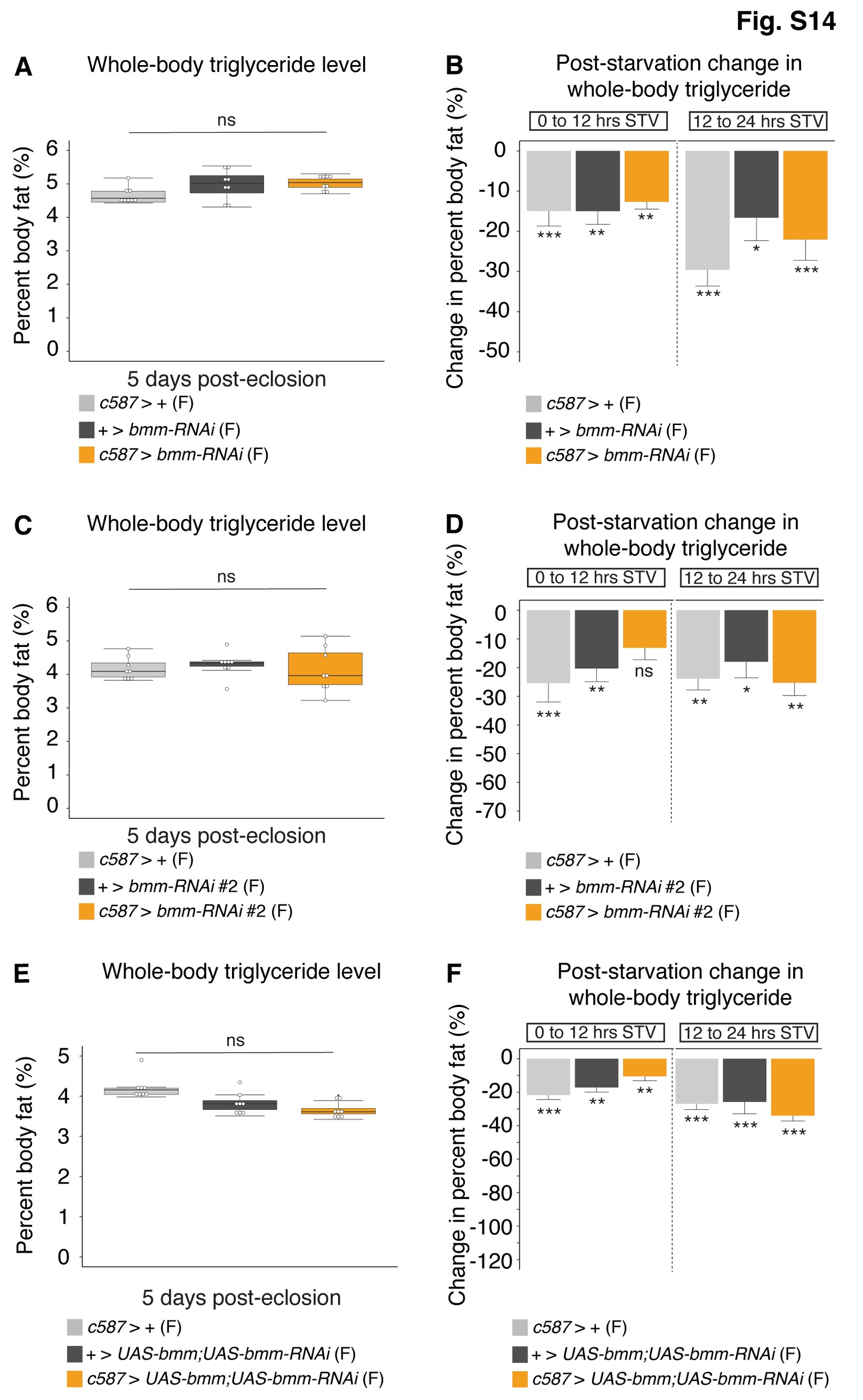

Supplement: S14 Fig — (A) Whole-body triglyceride storage in 5-day-old virgin females overexpressing UAS-bmm-RNAi in the somatic cells of the gonad (c587>UAS-bmm-RNAi) was not significantly different from age-matched control females (c587>+ and +>UAS-bmm-RNAi) (p = 0.083 and 0.96, respectively; one-way ANOVA followed by Tukey HSD test). (B) There was a modest but significant reduction in whole-body triglyceride levels in 5-day-old c587>+, +>UAS-bmm-RNAi, and c587>UAS-bmm-RNAi females between 0 and 12 hours STV (p = 2.3 × 10^−4^, 0.0094, and 0.0051, respectively; one-way ANOVA followed by Tukey HSD test) and between 12 and 24 hours STV (p = 0.0, 0.016, and 1.6 × 10^−5^, respectively; one-way ANOVA followed by Tukey HSD test). (C) Whole-body triglyceride storage in females with c587-GAL4-mediated overexpression of an additional UAS-bmm-RNAi#2 (VDRC #37877) transgene in the somatic cells of the gonad was not significantly different from control females (c587-GAL4>+ and +>UAS-bmm-RNAi#2) (p = 0.98 and 0.78, respectively; one-way ANOVA followed by Tukey HSD test). (D) Triglyceride breakdown post-starvation among c587-GAL4>UAS-bmm-RNAi#2 (VDRC #37877) females and c587-GAL4>+ and +>UAS-bmm-RNAi#2 controls showed a modest decrease of similar magnitude between both 0 and 12 hours and 12 and 24 hours STV (p = 0.096, 1.3 × 10^−4^, and 0.0013 for 0–12 hours and 0.004, 0.0049, and 0.022 for 12–24 hours, respectively; one-way ANOVA followed by Tukey HSD test). (E) Whole-body triglyceride levels in 5-day-old virgin c587>UAS-bmm;UAS-bmm-RNAi females were not significantly different among c587-GAL4>+ and +>UAS-bmm;UAS-bmm-RNAi controls (p = 8.5 × 10^−4^ and 0.38, respectively; one-way ANOVA followed by Tukey HSD test). (F) Triglyceride breakdown post-starvation among 5-day-old virgin c587>UAS-bmm;UAS-bmm-RNAi females and control females (c587>+ and +>UAS-bmm;UAS-bmm-RNAi) showed a modest decrease of a similar magnitude at both 0–12 hours or 12–24 hours STV (p = 0.0043, 1.7 × 10^−6^, and 0.0027 for 0–12 hours and 0 [file pbio.3000595.s014.tif]

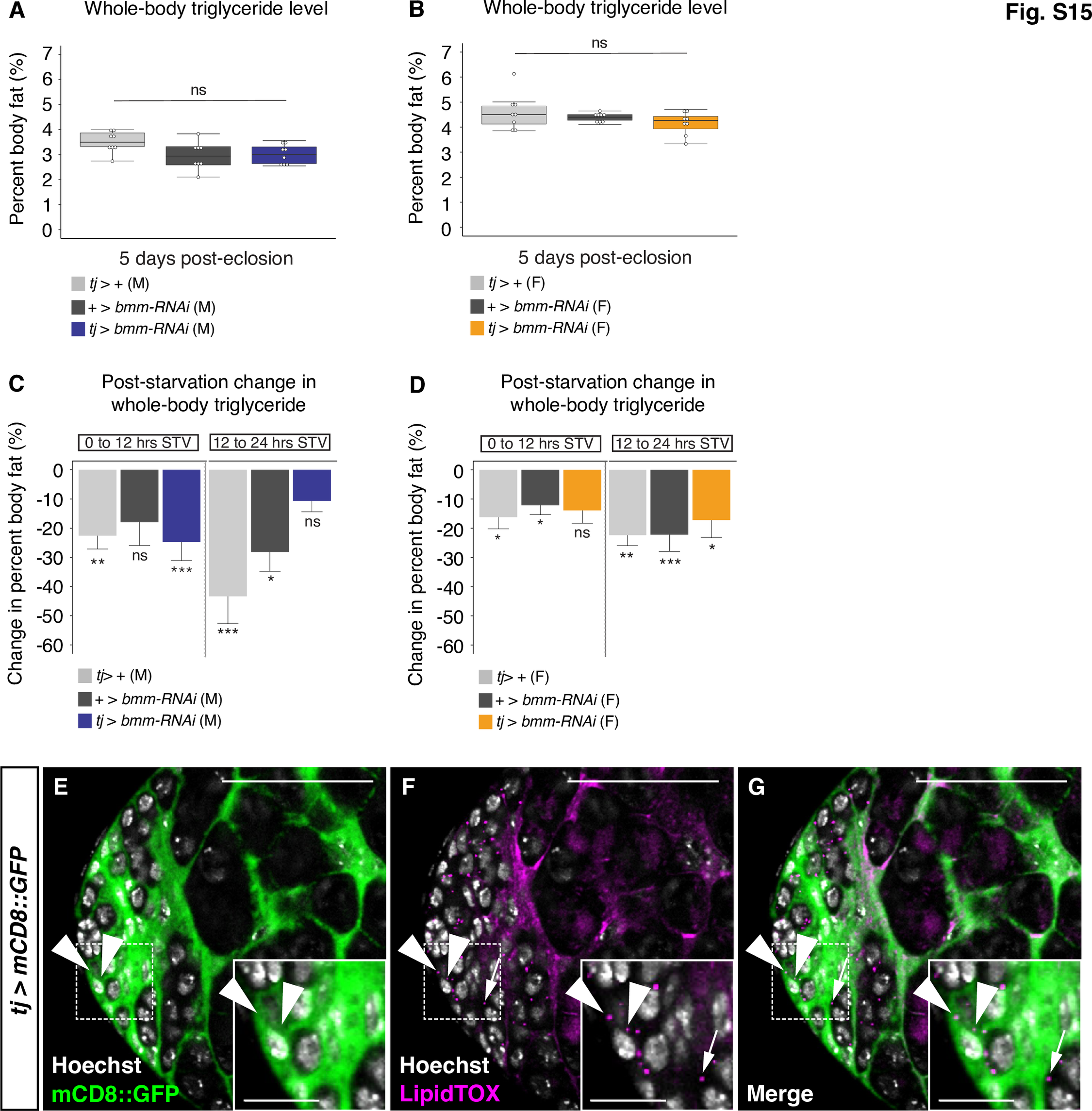

Supplement: S15 Fig — (A) Whole-body triglyceride storage in 5-day-old virgin males overexpressing UAS-bmm-RNAi in the somatic cells of the gonad (tj>UAS-bmm-RNAi) was not significantly different from age-matched control males (tj>+ and +>UAS-bmm-RNAi) (p = 0.098 and 0.97, respectively; one-way ANOVA followed by Tukey HSD test). (B) Whole-body triglyceride storage in 5-day-old virgin females overexpressing UAS-bmm-RNAi in the somatic cells of the gonad (tj>UAS-bmm-RNAi) was not significantly different from age-matched control females (tj>+ and +>UAS-bmm-RNAi) (p = 0.2 and 0.66, respectively; one-way ANOVA followed by Tukey HSD test). (C) Between 0 and 12 hours STV, the magnitude of triglyceride breakdown in tj>UAS-bmm-RNAi was similar to tj>+ and +>UAS-bmm-RNAi control males; however, between 12 and 24 hours STV, there was no significant decrease in triglyceride levels in tj>UAS-bmm-RNAi males, in contrast to tj>+ and +>UAS-bmm-RNAi control males, in which we observed a significant decrease in triglyceride storage STV (p = 9.7 × 10^−4^, 0.0018, and 0.099 for 0–12 hours and 0.37, 1.9 × 10^−5^, and 0.028 for 12–24 hours, respectively; one-way ANOVA followed by Tukey HSD test). (D) Triglyceride breakdown post-starvation among 5-day-old virgin tj>UAS-bmm-RNAi females and tj>+ and +>UAS-bmm-RNAi control females was modestly decreased by a similar magnitude at both 0–12 hours or 12–24 hours STV (p = 0.058, 0.026, and 0.022 for 0–12 hours and 0.042, 0.0093, and 3.7 × 10^−4^ for 12–24 hours, respectively; one-way ANOVA followed by Tukey HSD test). (E–G) We used tj-GAL4 to drive the expression of a membrane-bound GFP (UAS-mCD8::GFP) in the somatic cells of the gonad. The presence of lipid droplets within the GFP-marked boundary of the somatic cell indicates that lipid droplets are present in the somatic cells of the gonad. Non-GFP-positive droplets (arrow) likely represent lipid droplets in the germline cells. The image represents a single confocal slice from the Drosophila male testis. Scale bars = 50 [file pbio.3000595.s015.tif]

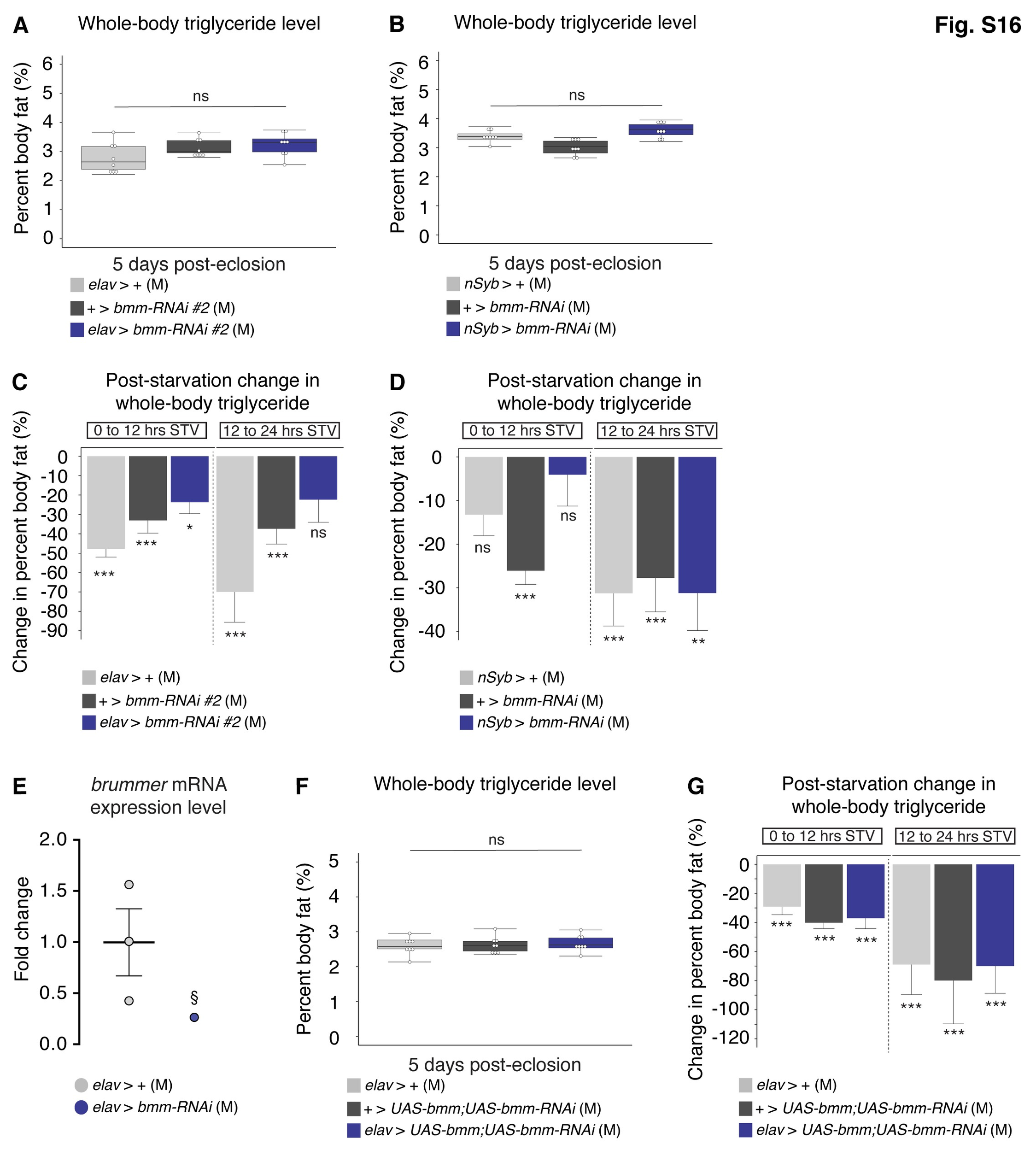

Supplement: S16 Fig — (A) Whole-body triglyceride storage in males with elav-GAL4-mediated overexpression of an additional UAS-bmm-RNAi#2 (VDRC #37877) transgene in neurons was not significantly different than in control males (elav-GAL4>+ and +>UAS-bmm-RNAi#2) (p = 0.11 and 0.91, respectively; one-way ANOVA followed by Tukey HSD test). (B) Whole-body triglyceride storage in males with nSyb-GAL4-mediated overexpression of the UAS-bmm-RNAi transgene in neurons was not significantly different than in control males (nSyb-GAL4>+ and +>UAS-bmm-RNAi) (p = 0.19 and 2.1 × 10^−4^, respectively; one-way ANOVA followed by Tukey HSD test). (C) The decrease in whole-body triglyceride levels in elav-GAL4>UAS-bmm-RNAi#2 (VDRC #37877) males was similar to elav-GAL4>+ and +>UAS-bmm-RNAi#2 control males between 0 and 12 hours STV (p = 0.01, 4.0 × 10^−7^, and 1.0 × 10^−5^, respectively; one-way ANOVA followed by Tukey HSD test). Triglyceride breakdown between 12 and 24 hours STV was blocked in elav-GAL4>UAS-bmm-RNAi#2 males, whereas triglyceride levels in control males during this interval significantly decreased (p = 0.084, 2.6 × 10^−5^, and 3.3 × 10^−4^, respectively; one-way ANOVA followed by Tukey HSD test). (D) The decrease in whole-body triglyceride levels in nSyb-GAL4>UAS-bmm-RNAi males was blunted compared with nSyb-GAL4>+ and +>UAS-bmm-RNAi control males between 0 and 12 hours STV (p = 0.86, 0.051, and 4.3 × 10^−5^, respectively; one-way ANOVA followed by Tukey HSD test); however, triglyceride breakdown between 12 and 24 hours STV in nSyb-GAL4>UAS-bmm-RNAi males was similar in magnitude to control males (nSyb>+ and +>UAS-bmm-RNAi) (p = 0.002, 1.2 × 10^−4^, and 7.0 × 10^−4^, respectively; one-way ANOVA followed by Tukey HSD test). (E) In dissected brains from elav>UAS-bmm-RNAi males, we found that bmm transcript levels were undetectable in three out of four samples, whereas we observed amplification at a higher cycle number in three elav>+ samples. (§) Because of this dramatic decrease in bmm transcript levels in [file pbio.3000595.s016.tif]

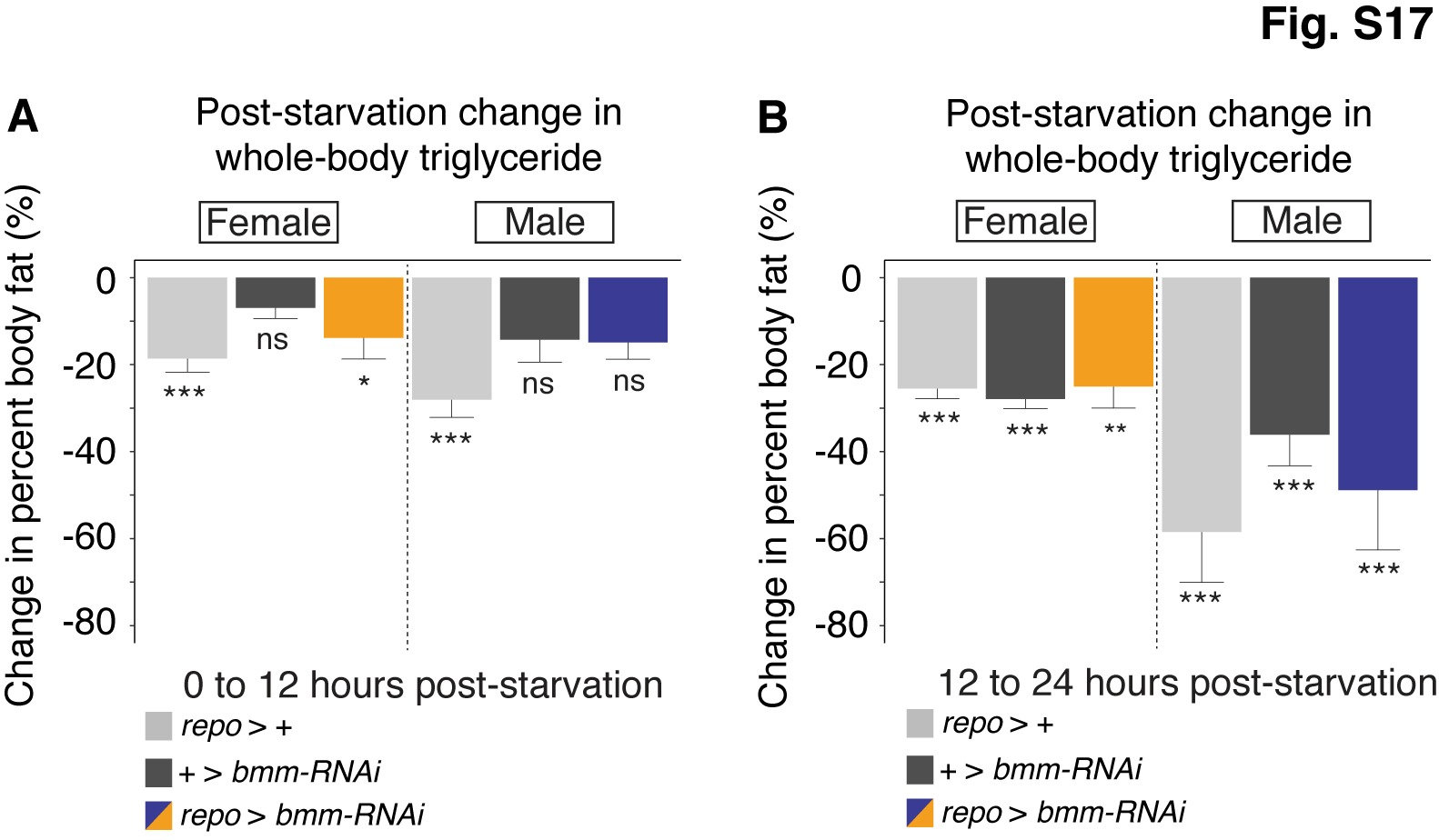

Supplement: S17 Fig — (A) Triglyceride breakdown post-starvation was modestly decreased by a similar magnitude in 5-day-old virgin repo>UAS-bmm-RNAi females compared with repo-GAL4>+ and +>UAS-bmm-RNAi control females at 0–12 hours post-starvation (p = 0.047, 2.3 × 10^−5^, and 0.096, respectively; one-way ANOVA followed by Tukey HSD test) and between repo>UAS-bmm-RNAi males and repo-GAL4>+ and +>UAS-bmm-RNAi control males during the same interval (p = 0.085, 6.9 × 10^−6^, and 0.057, respectively; one-way ANOVA followed by Tukey HSD test). (B) Between 12 and 24 hours post-starvation, we observed only a modest reduction in female triglyceride levels post-starvation in all genotypes (repo>UAS-bmm-RNAi, repo-GAL4>+, and +>UAS-bmm-RNAi), and the magnitude of this reduction was similar between genotypes (p = 0.0019, 4.8 × 10^−6^, and 2.0 × 10^−7^, respectively; one-way ANOVA followed by Tukey HSD test). Similarly, although there was a significant decrease in triglyceride levels post-starvation in repo>UAS-bmm-RNAi males, repo-GAL4>+, and +>UAS-bmm-RNAi controls (p = 8.8 × 10^−6^, 0.0, and 8.2 × 10^−5^, respectively; one-way ANOVA followed by Tukey HSD test), the magnitude of this decrease was similar for all genotypes. Asterisks indicate a significant difference between two sexes, two genotypes, or two time points (*p < 0.05, **p < 0.01, ***p < 0.001). See S1 Table for list of all multiple comparisons and p-values. Error bars on graphs represent COE. Quantitative measurements underlying all graphs are available in S1 Data. bmm, brummer; COE, coefficient of error; HSD, honest significant difference; ns indicates no significant difference between two sexes, two genotypes, or time points; repo, reversed polarity; UAS, upstream activation sequence. (TIF) [file pbio.3000595.s017.tif]

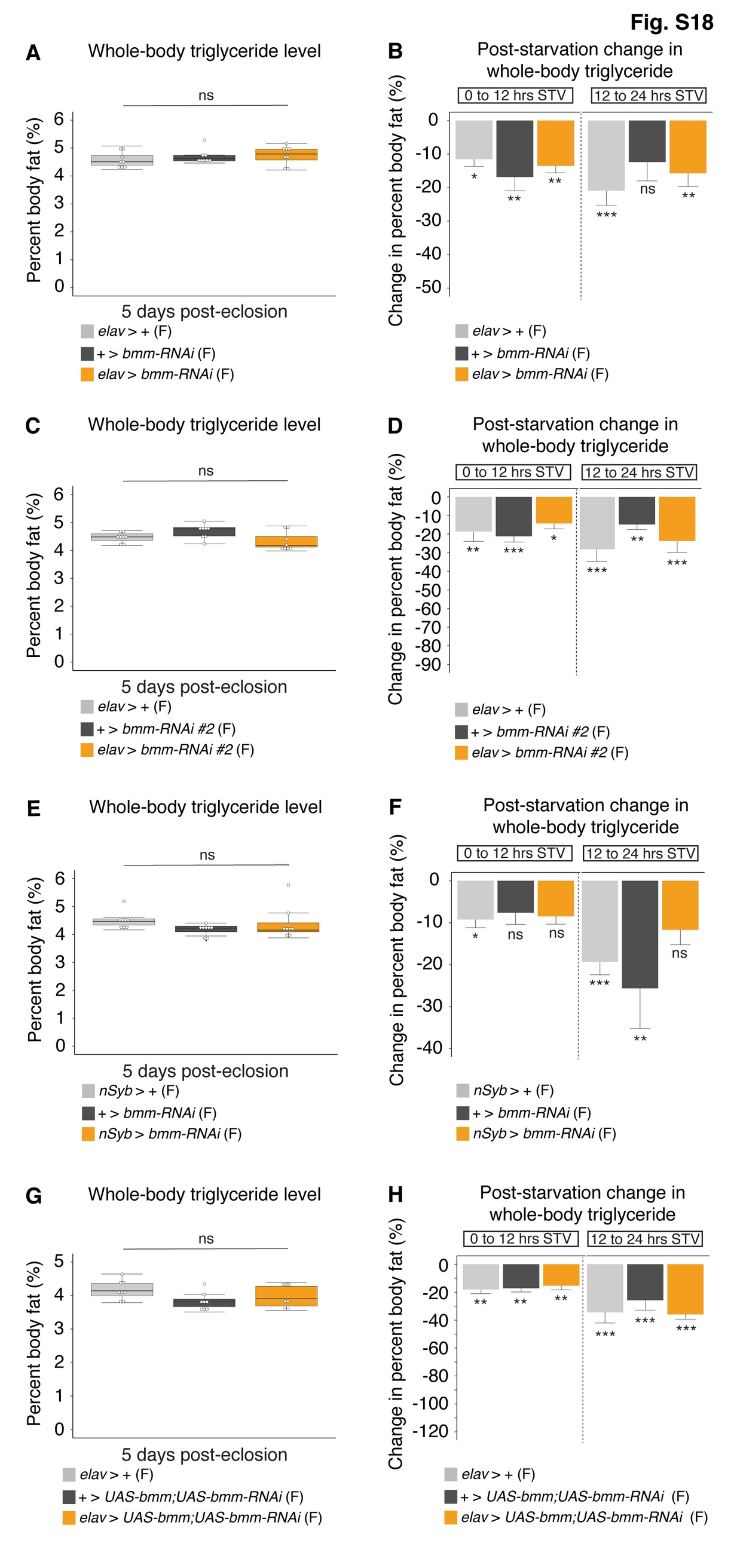

Supplement: S18 Fig — (A) Whole-body triglyceride storage in 5-day-old virgin females overexpressing UAS-bmm-RNAi in the postmitotic neurons (elav>UAS-bmm-RNAi) was not significantly different from age-matched control females (elav>+ and +>UAS-bmm-RNAi) (p = 0.54 and 0.95, respectively; one-way ANOVA followed by Tukey HSD test). (B) There was a significant reduction in whole-body triglyceride levels in 5-day-old elav>+, +>UAS-bmm-RNAi, and elav>UAS-bmm-RNAi females between 0 and 12 hours STV (p = 0.026, 0.0038, and 0.0013, respectively; one-way ANOVA followed by Tukey HSD test). Between 12 and 24 hours STV, 5-day-old elav>+, +>UAS-bmm-RNAi, and elav>UAS-bmm-RNAi females modestly decreased triglyceride levels by similar magnitudes (p = 1.2 × 10^−4^, 0.15, and 0.0012, respectively; one-way ANOVA followed by Tukey HSD test). (C) Whole-body triglyceride storage in females with elav-GAL4-mediated overexpression of an additional UAS-bmm-RNAi#2 (VDRC #37877) transgene in neurons was not significantly different from control females (elav-GAL4>+ and +>UAS-bmm-RNAi#2) (p = 0.56 and 0.035, respectively; one-way ANOVA followed by Tukey HSD test). (D) There was a modest decrease in triglyceride levels between 0 and 12 hours and 12 and 24 hours STV among elav-GAL4>UAS-bmm-RNAi#2 (VDRC #37877) females and elav-GAL4>+ and +>UAS-bmm-RNAi#2 controls (p = 0.011, 0.0027, and 1.4 × 10^−6^ for 0–12 hours STV and 4.6 × 10^−4^, 3.4 × 10^−4^, and 0.002 for 12–24 hours STV, respectively; one-way ANOVA followed by Tukey HSD test). (E) Whole-body triglyceride levels in 5-day-old virgin nSyb>UAS-bmm-RNAi females were not significantly different to nSyb-GAL4>+ and +>UAS-bmm-RNAi controls (p = 0.85 and 0.52, respectively; one-way ANOVA followed by Tukey HSD test). (F) Triglyceride breakdown post-starvation was modestly decreased by a similar magnitude among genotypes in 5-day-old virgin nSyb>UAS-bmm-RNAi females and control females (nSyb>+ and +>UAS-bmm-RNAi) between 0 and 12 hours STV (p = 0.21, 0.017, and 0.43, respectively [file pbio.3000595.s018.tif]

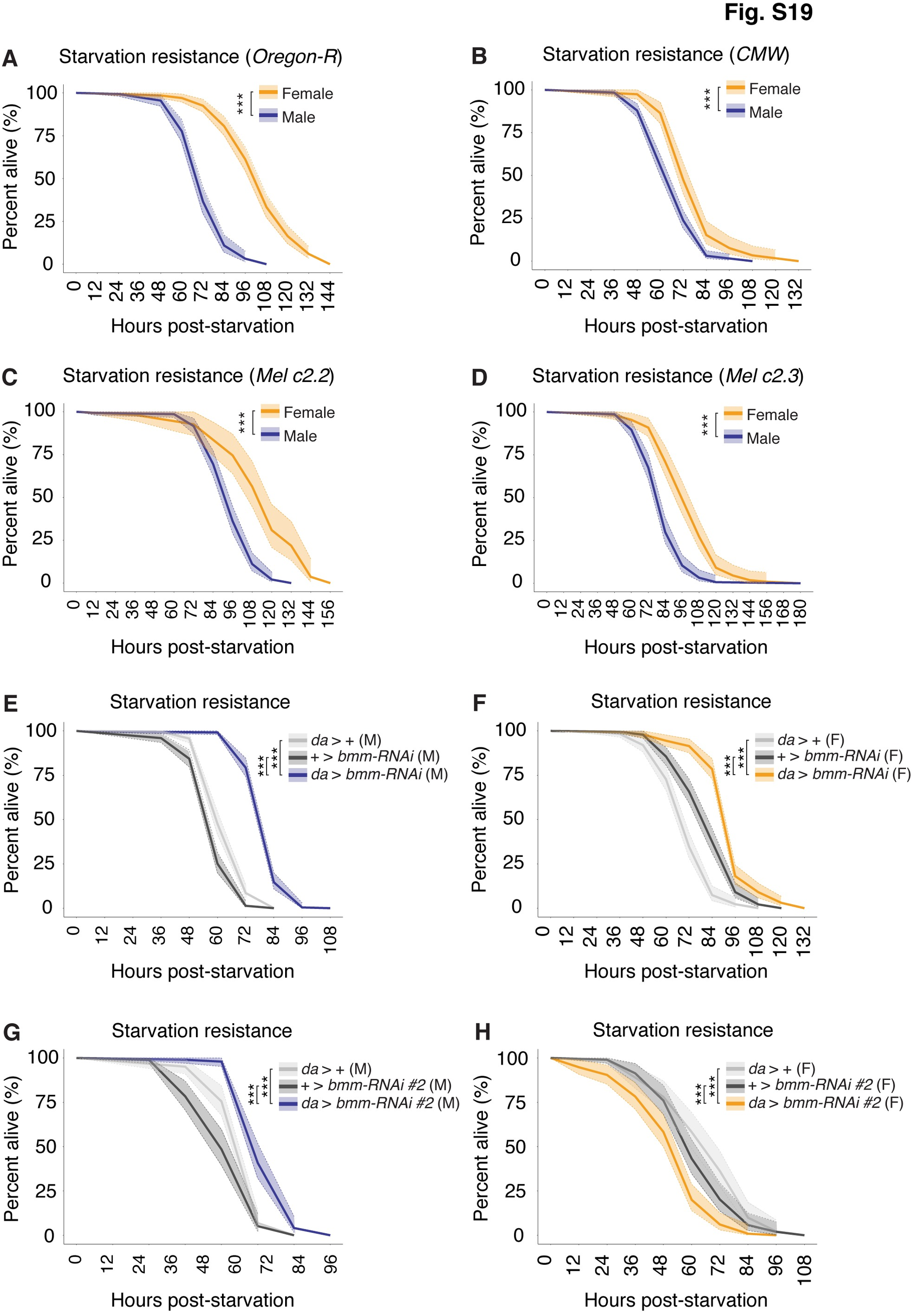

Supplement: S19 Fig — (A) Median survival post-starvation was significantly higher in 5-day-old virgin Oregon-R females than in virgin Oregon-R males (p = 2 × 10^−16^; Log-rank test with Bonferroni correction for multiple comparison; n > 156). (B) Median survival post-starvation was significantly higher in 5-day-old virgin CMW wild-caught females than in virgin CMW males (p = 6 × 10^−10^; Log-rank test with Bonferroni correction for multiple comparison; n > 118). (C, D) Median survival post-starvation was significantly higher in 5-day-old virgin females than males in two isofemale strains (Mel c2.2: p = 4.4 × 10^−11^; Log-rank test with Bonferroni correction for multiple comparison; n > 55 and Mel c2.3: p = 1.4 × 10^−13^; Log-rank test with Bonferroni correction for multiple comparison; n > 110). (E, F) Median survival post-starvation was significantly higher in virgin males (E) and females (F), with ubiquitous overexpression of UAS-bmm-RNAi compared with control males (da>+ and +>UAS-bmm-RNAi) (p = 2 × 10^−16^ and 2 × 10^−16^ respectively; Log-rank test with Bonferroni correction for multiple comparison; n > 223) and females (p = 2 × 10^−16^ and 1.2 × 10^−13^ respectively; Log-rank test with Bonferroni correction for multiple comparisons; n > 176). (G, H) Median survival post-starvation was significantly higher in virgin males (G) and significantly lower in females (H) with ubiquitous overexpression of UAS-bmm-RNAi#2 (BDSC #25926) compared to control males (da>+ and +>UAS-bmm-RNAi#2) (p = 7.7 × 10^−11^ and 2 × 10^−16^, respectively; Log-rank test with Bonferroni correction for multiple comparison; n > 97) and control females (p = 9.6 × 10^−9^ and 4.9 × 10^−5^, respectively; Log-rank test with Bonferroni correction for multiple comparisons; n > 91). The p-values are listed in the following order: difference between the GAL4/UAS genotype and the GAL4 control/difference between the GAL4/UAS genotype and the UAS control. Asterisks indicate a significant difference between two sexes, two genotypes, or two time poin [file pbio.3000595.s019.tif]

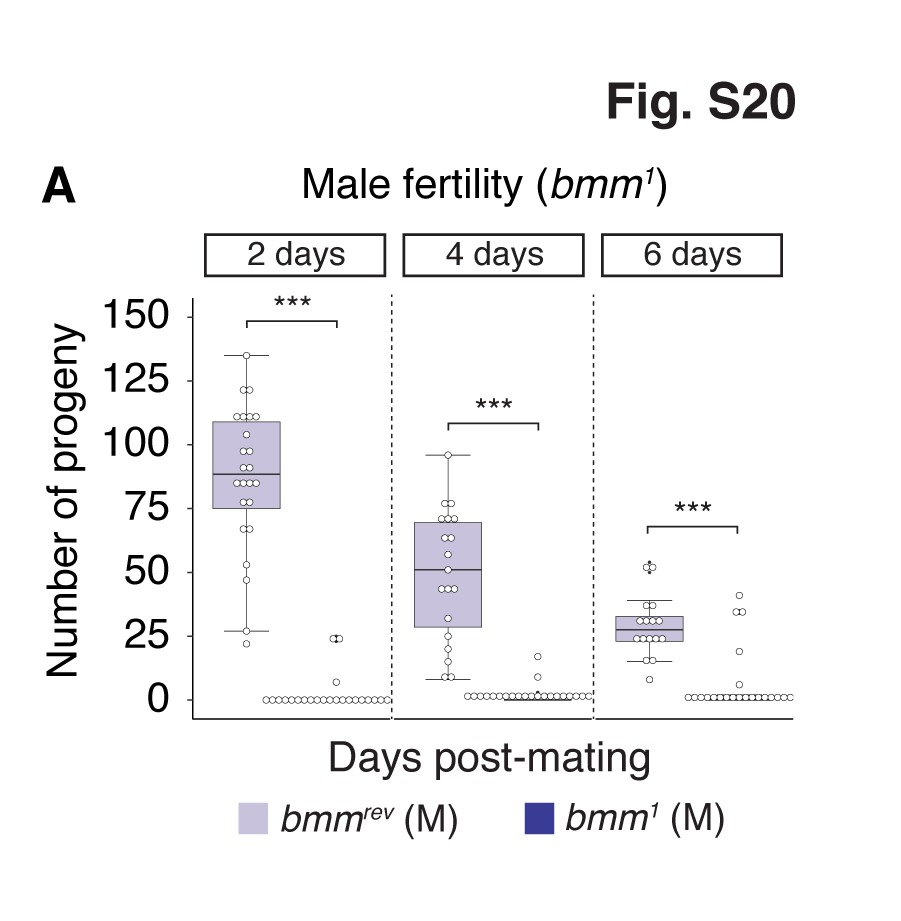

Supplement: S20 Fig — (A) Males with whole-body loss of brummer have a significantly decreased number of progeny after 2 days, 4 days, and 6 days of mating (p = 2.2 × 10^−16^, 9.7 × 10^−12^, and 4.6 × 10^−6^, respectively; Student t test at each time point). Asterisks indicate a significant difference between two sexes, two genotypes, or two time points (*p < 0.05, **p < 0.01, ***p < 0.001). See S1 Table for list of all multiple comparisons and p-values. Error bars on graphs represent SEM. Quantitative measurements underlying all graphs are available in S4 Data. M, male; ns, no significant difference between two sexes, two genotypes, or time points. (TIF) [file pbio.3000595.s020.tif]

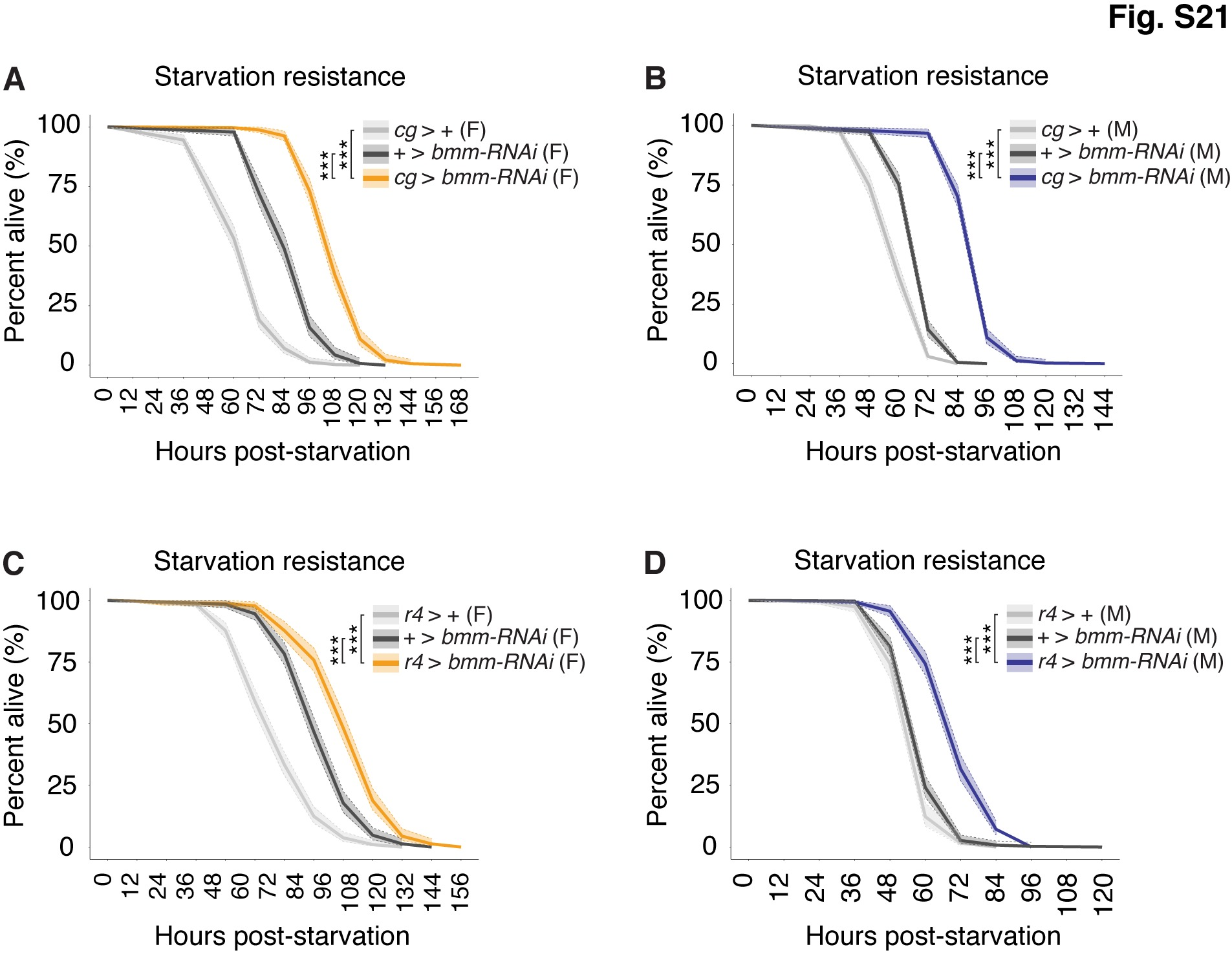

Supplement: S21 Fig — (A, B) Median survival post-starvation was significantly higher in virgin females (A) and males (B) with fat body–specific bmm inhibition (cg>UAS-bmm-RNAi) compared with control females (cg>+ and +>UAS-bmm-RNAi) (p = 2 × 10^−16^ and 2 × 10^−16^, respectively; Log-rank test with Bonferroni correction for multiple comparisons; n > 279) and males (p = 2 × 10^−16^ and 2 × 10^−16^, respectively; Log-rank test with Bonferroni correction for multiple comparisons; n > 365). (C, D) Median survival post-starvation was significantly higher in virgin females (C) and males (D) with fat body–specific bmm inhibition using a second GAL4 driver (r4>UAS-bmm-RNAi) compared with control females (r4>+ and +>UAS-bmm-RNAi) (p = 2 × 10^−16^ and 2 × 10^−16^, respectively; Log-rank test with Bonferroni correction for multiple comparisons; n > 314) and males (p = 2 × 10^−16^ and 2 × 10^−16^, respectively; Log-rank test with Bonferroni correction for multiple comparisons; n > 195). The p-values are listed in the following order: difference between the GAL4/UAS genotype and the GAL4 control/difference between the GAL4/UAS genotype and the UAS control. Asterisks indicate a significant difference between two sexes, two genotypes, or two time points (*p < 0.05, **p < 0.01, ***p < 0.001). Shaded areas represent the 95% confidence interval. See S1 Table for list of all multiple comparisons and p-values; quantitative measurements underlying all graphs are available in S4 Data. bmm, brummer; cg, collagen; F, female; M, male; ns, no significant difference between two sexes, two genotypes, or time points; UAS, upstream activation sequence. (TIF) [file pbio.3000595.s021.tif]

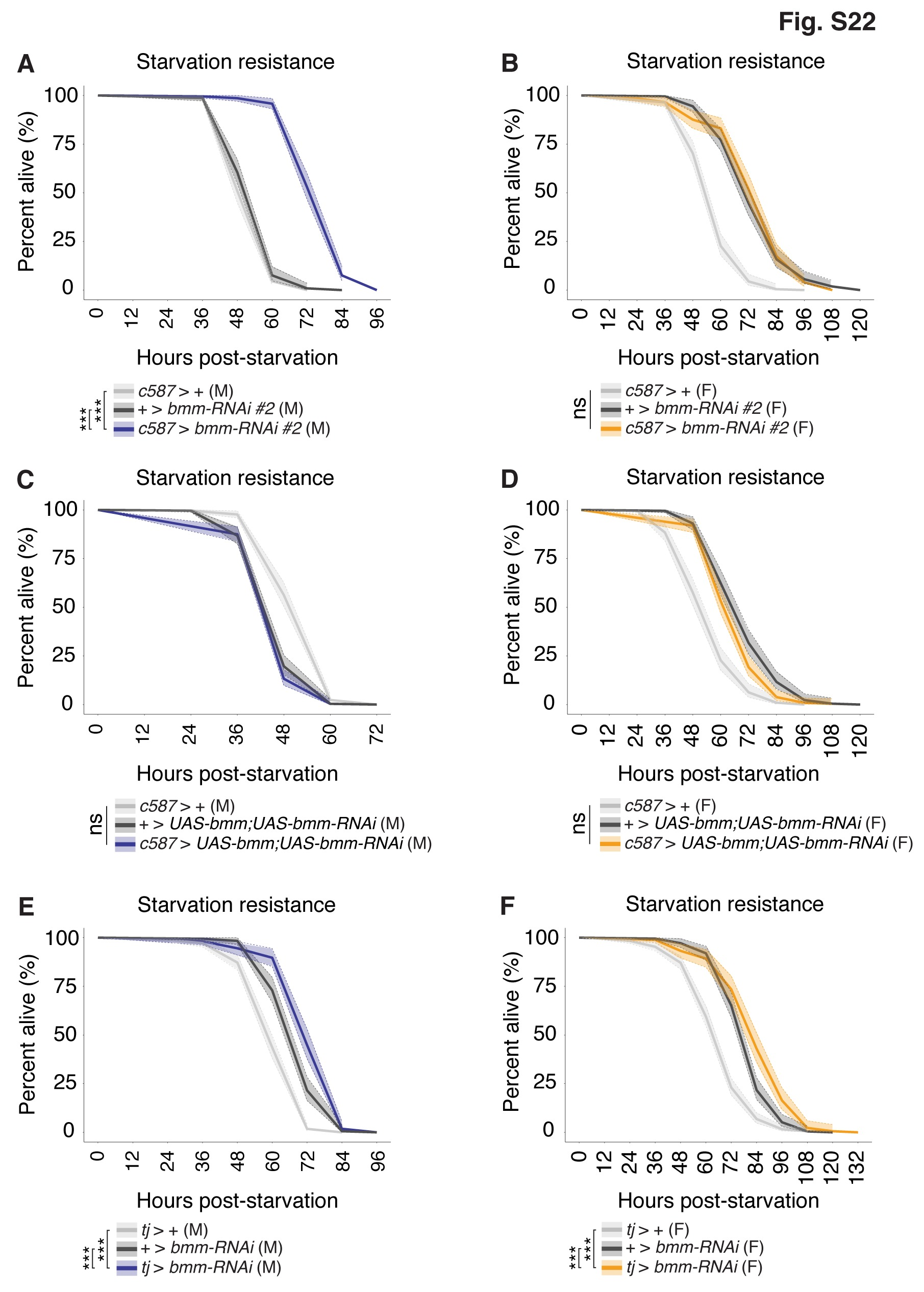

Supplement: S22 Fig — (A, B) Median survival post-starvation was significantly higher in virgin males (A) but not significantly different in virgin females (B) with bmm inhibition in the somatic cells of the gonad (c587>UAS-bmm-RNAi#2 [VDRC #37877]) compared with control males (c587>+ and +>UAS-bmm-RNAi#2) (p = 2 × 10^−16^ and 2 × 10^−16^, respectively; Log-rank test with Bonferroni correction for multiple comparisons; n > 212) and females (p = 2 × 10^−16^ and 1.0, respectively; Log-rank test with Bonferroni correction for multiple comparisons; n > 201). (C, D) Median survival post-starvation was not significantly changed when UAS-bmm and UAS-bmm-RNAi were simultaneously overexpressed by a driver for the somatic cells of the gonad (c587>UAS-bmm;UAS-bmm-RNAi) in both males (C) and females (D) compared with control males (c587>+ and +>UAS-bmm;UAS-bmm-RNAi) (p = 2 × 10^−16^ and 0.57, respectively; Log-rank test with Bonferroni correction for multiple comparisons; n > 249) and females (p = 9.6 × 10^−16^ and 0.0035, respectively; Log-rank test with Bonferroni correction for multiple comparisons; n > 207). (E, F) Median survival post-starvation was significantly increased when UAS-bmm-RNAi was overexpressed by a second driver for the somatic cells of the gonad (tj>UAS-bmm-RNAi) in both females (E) and males (F) compared with control males (tj>+ and +>UAS-bmm-RNAi) (p = 2 × 10^−16^ and 1.3 × 10^−6^, respectively; Log-rank test with Bonferroni correction for multiple comparisons; n > 165) and females (p = 2 × 10^−16^ and 0.00012, respectively; Log-rank test with Bonferroni correction for multiple comparisons; n > 177). The p-values are listed in the following order: difference between the GAL4/UAS genotype and the GAL4 control/difference between the GAL4/UAS genotype and the UAS control. Asterisks indicate a significant difference between two sexes, two genotypes, or two time points (*p < 0.05, **p < 0.01, ***p < 0.001). Shaded areas represent the 95% confidence interval. See S1 Table for a list of all multiple [file pbio.3000595.s022.tif]

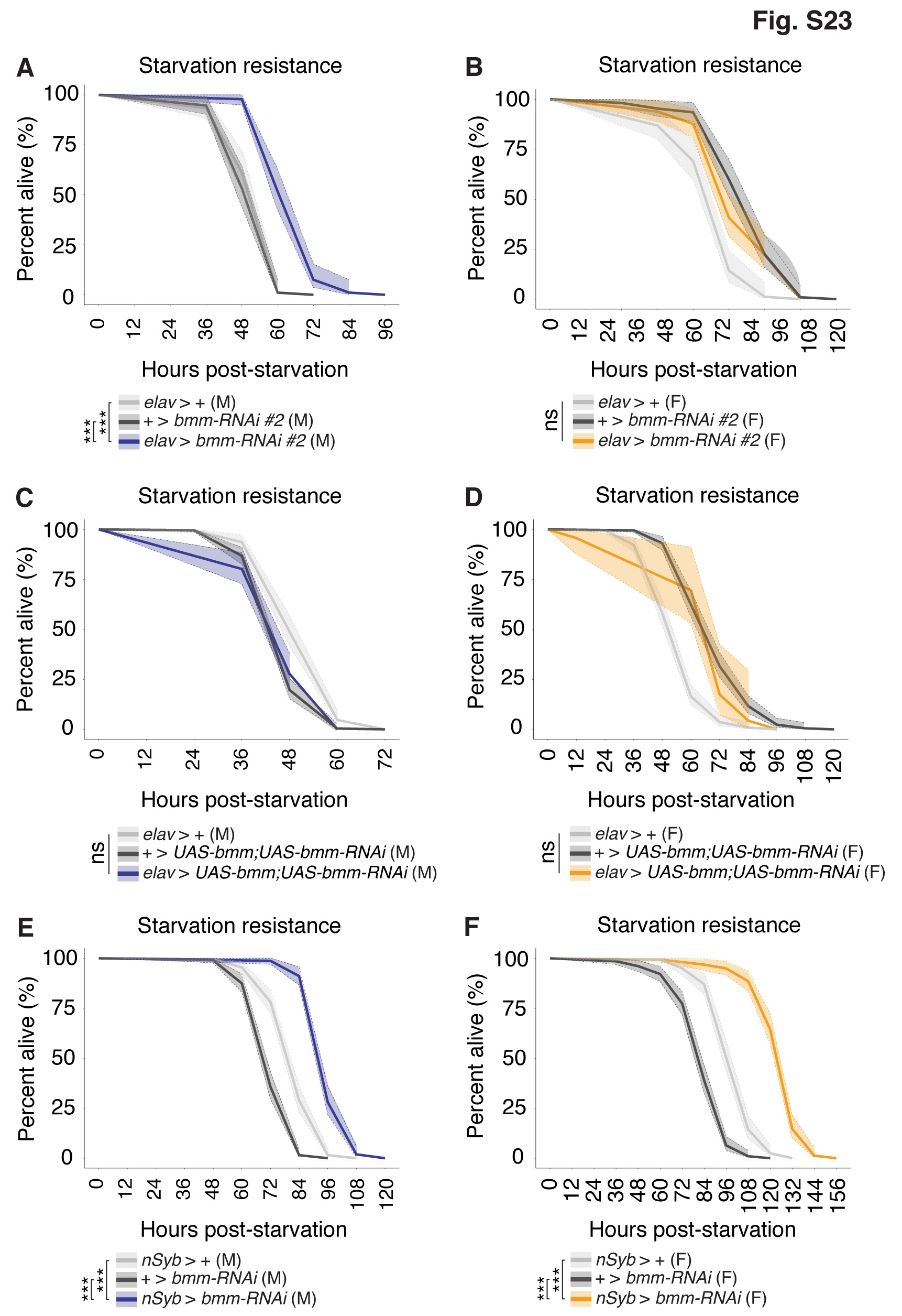

Supplement: S23 Fig — (A, B) Median survival post-starvation was significantly higher in virgin males (A) but not significantly different in virgin females (B) with bmm inhibition in the neurons (elav>UAS-bmm-RNAi#2 [VDRC #37877]) compared with control males (elav>+ and +>UAS-bmm-RNAi#2) (p = 2 × 10^−16^ and 2 × 10^−16^, respectively; Log-rank test with Bonferroni correction for multiple comparisons; n > 90) and females (p = 3.4 × 10^−6^ and 0.41, respectively; Log-rank test with Bonferroni correction for multiple comparisons; n > 81). (C, D) Median survival post-starvation was not significantly changed when UAS-bmm and UAS-bmm-RNAi were simultaneously overexpressed by a neuronal driver (elav>UAS-bmm;UAS-bmm-RNAi) in both males (C) and females (D) compared with control males (elav>+ and +>UAS-bmm;UAS-bmm-RNAi) (p = 4.6 × 10^−5^ and 1, respectively; Log-rank test with Bonferroni correction for multiple comparisons; n > 101) and females (p = 8.9 × 10^−6^ and 1, respectively; Log-rank test with Bonferroni correction for multiple comparisons; n > 23). (E, F) Median survival post-starvation was significantly increased when UAS-bmm-RNAi was overexpressed by a second neuronal driver (nSyb>UAS-bmm-RNAi) in both males (E) and females (F) compared with control males (nSyb>+ and +>UAS-bmm-RNAi) (p = 2 × 10^−16^ and 2 × 10^−16^, respectively; Log-rank test with Bonferroni correction for multiple comparisons; n > 157) and females (p = 2 × 10^−16^ and 2 × 10^−16^, respectively; Log-rank test with Bonferroni correction for multiple comparisons; n > 157). The p-values are listed in the following order: difference between the GAL4/UAS genotype and the GAL4 control/difference between the GAL4/UAS genotype and the UAS control. Asterisks indicate a significant difference between two sexes, two genotypes, or two time points (*p < 0.05, **p < 0.01, ***p < 0.001). Shaded areas represent the 95% confidence interval. See S1 Table for list of all multiple comparisons and p-values; quantitative measurements underlying all graphs ar [file pbio.3000595.s023.tif]

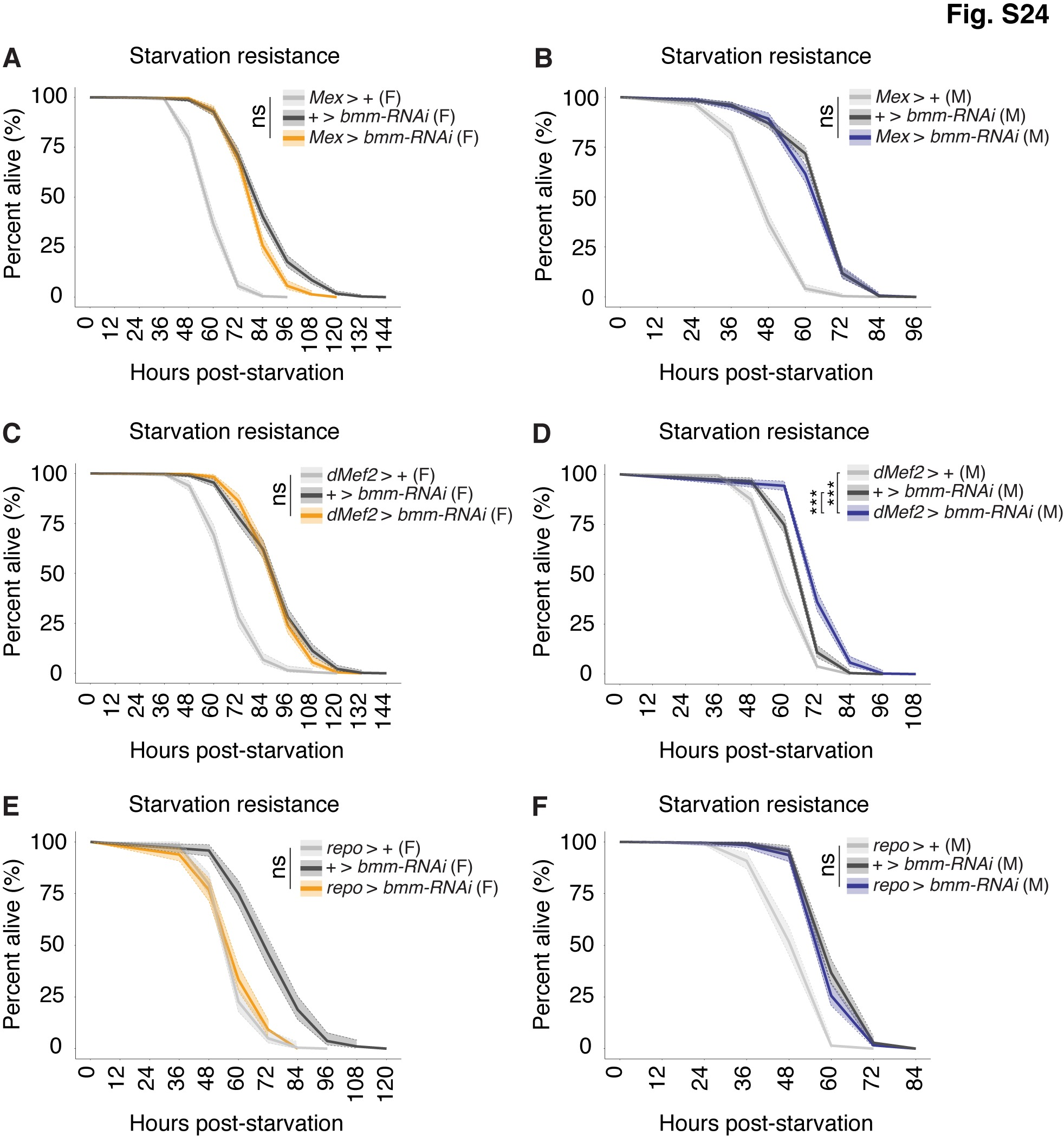

Supplement: S24 Fig — (A, B) Median survival post-starvation showed no significant change in virgin females (A) and males (B) with gut-specific bmm inhibition (Mex>UAS-bmm-RNAi) compared with control females (Mex>+ and +>UAS-bmm-RNAi) (p = 2 × 10^−16^ and 1.1 × 10^−7^, respectively; Log-rank test with Bonferroni correction for multiple comparison; n > 444) and males (p = 2 × 10^−16^ and 0.13 respectively; Log-rank test with Bonferroni correction for multiple comparison; n > 456). (C, D) Median survival post-starvation was unchanged in virgin females (C) and slightly increased in males (D) with muscle-specific bmm inhibition (dMef2>UAS-bmm-RNAi) compared with control females (dMef2>+ and +>UAS-bmm-RNAi) (p = 2 × 10^−16^ and 0.4, respectively; Log-rank test with Bonferroni correction for multiple comparison; n > 340) and males (p = 2 × 10^−16^ and 2 × 10^−16^, respectively; Log-rank test with Bonferroni correction for multiple comparison; n > 382). (E, F) Median survival post-starvation showed no significant change in virgin females (E) and males (F) with glia-specific bmm inhibition (repo>UAS-bmm-RNAi) compared with control females (repo>+ and +>UAS-bmm-RNAi) (p = 0.43 and 2 × 10^−16^, respectively; Log-rank test with Bonferroni correction for multiple comparison; n > 191) and males (p = 2 × 10^−16^ and 0.02, respectively; Log-rank test with Bonferroni correction for multiple comparison; n > 207). The p-values are listed in the following order: difference between the GAL4/UAS genotype and the GAL4 control/difference between the GAL4/UAS genotype and the UAS control. Asterisks indicate a significant difference between two sexes, two genotypes, or two time points (*p < 0.05, **p < 0.01, ***p < 0.001). Shaded areas represent the 95% confidence interval. See S1 Table for a list of all multiple comparisons and p-values; quantitative measurements underlying all graphs are available in S4 Data. bmm, brummer; F, female; M, male; Mex, midgut expression 1; dMef2, myocyte enhancer factor 2, ns, no significant diffe [file pbio.3000595.s024.tif]
