# Supplementary material for: A role for triglyceride lipase brummer in the regulation of sex differences in Drosophila fat storage and breakdown
Source: PLoS Biol. 2020 Jan 21;18(1):e3000595. doi: 10.1371/journal.pbio.3000595 (PMC6994176; doi:10.1371/journal.pbio.3000595)
Supplement: S1 Materials and Methods — (DOCX) [file pbio.3000595.s032.docx]

**SUPPLEMENTAL MATERIALS AND METHODS**

**Fly media**

For 1L of fly media:

| Ingredient | Amount |
| --- | --- |
| H_2_O | 1 L |
| White sugar | 20.5 g |
| D-glucose | 70.9 g |
| Cornmeal | 48.5 g |
| Yeast | 30.3 g |
| CaCl_2_-2H_2_O | 0.5 g |
| MgSO_4_-7H_2_O | 0.5 g |
| Agar | 4.6 g |
| Propionic acid (836 ml per 1L) | 5.9 ml |
| Phosphoric acid (83 ml per 1L) | 5.9 ml |

**Triglyceride assay**

5 flies were frozen on dry ice and stored at -80°C until processed. Flies were homogenized using 100 μl of glass beads (Sigma, 11079110) in 200 μl of 0.1% Tween (Amresco, 0777-1L) in 1X PBS at 8.0m/s for 5 seconds (OMNI International - Bead Ruptor 24). For each sample, 30 μl of homogenate was transferred in duplicate to a 96-well plate and heat treated at 70°C for 10 minutes (Bio-Rad T100 Thermal Cycler). Standard curve was prepared using glycerol standard (Sigma, G7793) for concentrations of 0 mg/ml, 0.125 mg/ml, 0.25 mg/ml, 0.5 mg/ml, 1 mg/ml, and 2 mg/ml. 30 μl of each standard curve concentration was transferred to the same 96-well plate in duplicate, after heat treatment. 30 μl of triglyceride reagent (Sigma, T2449) was added to 1 set of samples/standard curve and 0.1% Tween in 1x PBS was added to the other set of samples/standard curve. Samples were vortexed and incubated at 37°C for 40 minutes. After incubation, the 96-well plate was centrifuged at 3100 rpm for 3 min at 4°C (Mandel Scientific – Sorvall RT 6000D) to precipitate cellular debris. 7.5 μl of supernatant was transferred to a 384-well plate-reader plate and 25 μl of free glycerol reagent (Sigma, F6428) was added to each well. Plate was incubated at 37°C for 5 minutes then absorbance was read at 540 nm (Thermo Scientific – Multiskan FC Microplate Photometer).

**Protein assay**

5 flies were frozen on dry ice and stored at -80°C until processed. Flies were homogenized using 100 μl of glass beads (Sigma, 11079110) in 200 μl of 1X PBS at 8.0m/s for 5 seconds (OMNI International – Bead Ruptor 24). Samples were centrifuged at 3200 rpm for 3 min at 4°C (Thermo Scientific Heraeus Pico 21 Microcentrifuge) to precipitate cellular debris. 250 μl of 1X Bradford reagent (Bio-Rad, 500-0205) was added to each well of a 96-well plate-reader plate. 1 μl of sample was added in duplicate to wells containing 1X Bradford reagent and mixed thoroughly. 0 μl, 0.5 μl, 1 μl, 1.5 μl, 2 μl, 2.5 μl, 3.75 μl, and 6.25 μl of γ-globulin (Bio-Rad, 500-0208) was added to wells containing 1X Bradford reagent to generate a protein standard curve of concentrations 0 μg/ml, 2 μg/ml, 4 μg/ml, 6 μg/ml, 8 μg/ml, 10 μg/ml, 15 μg/ml, and 25 μg/ml. Plate was incubated for 5 minutes at room temperature then centrifuged at 3200 rpm for 3 min at 4°C (Mandel Scientific – Sorvall RT 6000D). Absorbance was read at 594 nm (Thermo Scientific – Multiskan FC Microplate Photometer).

**Glucose and glycogen assay**

5 flies were frozen on dry ice and stored at -80°C until processed. Flies were homogenized using 100 μl of glass beads (Sigma, 11079110) in 200 μl of 1X PBS at 8.0m/s for 5 seconds (OMNI International – Bead Ruptor 24). For each sample, 50 μl of homogenate was transferred to a 96-well plate and heat-treated at 70°C for 10 minutes (Bio-Rad T100 Thermal Cycler). The 96-well plate was centrifuged at 3200 rpm for 3 min at 4°C (Mandel Scientific – Sorvall RT 6000D) to precipitate cellular debris. A glucose standard curve was prepared using glucose standard from Glucose (GO) Assay Kit (Sigma, GAGO20) and a glycogen standard curve was prepared using glycogen (Thermo Fisher Scientific, AM9510) for concentrations of 0 mg/ml, 0.02 mg/ml, 0.04 mg/ml, 0.08 mg/ml, and 0.16 mg/ml. Glycogen reagent was made by adding 1 μl of Amyloglucosidase (Sigma, A1602) to 1 ml of GO reagent from GO Kit (Sigma, GAGO20). 30 μl of each standard curve concentration was added into a 96-well plate-reader plate. 6 μl of each sample and 24 μl of 1X PBS were loaded in duplicate to the same 96-well plate-reader plate. 100 μl of glycogen reagent was added to 1 set of samples and the glycogen standard curve. 100 μl of GO reagent was added to the other set of samples and the glucose standard curve. Samples were incubated at 37°C for 60 minutes. 100 μl of 12N H_2_SO_4_ was added to every well. Absorbance was read at 540 nm (Thermo Scientific – Multiskan FC Microplate Photometer).

**Larval fat cell quantification**

One newly eclosed male or female was dissected open through the abdomen in one well of a 24-well plate using 500 μl of a 1:100 dilution of BODIPY 493/503 (ThermoFisher, D3922) in 1X PBS and a 1:500 dilution of Hoechst 33342 (ThermoFisher, H3570) in 1X PBS. The larval fat cells were loosened from the carcass into the wells and the carcass was removed. The larval fat cells were incubated at room temperature for 40 min. Immediately after, images were acquired on a Zeiss AXIO ZoomV.16. The number of larval fat cells within each well was quantified using ImageJ.

**GAL4 specificity**

5-day-old virgin males and females were dissected in 1X PBS for their brains, head fat body, gut, abdominal fat body, and gonads. Tissues were fixed in 4% paraformaldehyde at room temperature for 30 min. After 2 x 5 min washes in 1X PBS, the dissected tissues were incubated in a 1:500 dilution of Hoechst 33342 (ThermoFisher, H3570) in 1X PBS for 30 min. Following 2 x 5 min washes in 1X PBS, tissues were mounted in 80% glycerol in 1X PBS. Images were acquired on a Zeiss AXIO ZoomV.16. nGFP expression was scored for each tissue on a ‘++++’ scale with ‘+’ meaning very low expression and ‘++++’ meaning very strong expression. If no nGFP expression was detected, it was scored with a ‘-‘

**High fat diet feeding**

20 newly eclosed *bmm^rev^* or *bmm^1^* females were transferred to vials containing 2 ml of either control diet or high fat diet containing 30% lard by weight. Flies were transferred to fresh food vials every other day and kept on their sides until collected and flash frozen for analysis.

**RNA extraction and cDNA synthesis**

10 flies were frozen on dry ice and stored at -80°C until processed. Flies were homogenized using 100 μl of glass beads (Sigma, 11079110) in 500 μl of TRIzol reagent (ambion, 15596018) at 8.0m/s for 5 seconds (OMNI International – Bead Ruptor 24). Homogenate was mixed vigorously with 100 μl of chloroform (Fisher Scientific, C294-1) then incubated at room temperature for 3 min before being centrifuged at 12,000g for 10 min at 4°C (Thermo Scientific Heraeus Pico 21 Microcentrifuge). The aqueous top layer was transferred to a clean 1.5 ml microcentrifuge tube. Equal volume of isopropanol (Fisher Scientific, A415-4) was added to each sample, vortexed, and incubated at room temperature for 10-20 min. Samples were centrifuged at 12,000g for 10 min at 4°C. The supernatant was removed and the pellet was washed with 500 μl of 75% ethanol. Samples were spun at 12,000g for 10 min at room temperature. Supernatant was removed and pellet was allowed to dry at room temperature. RNA pellet was re-suspended in 200 μl of molecular biology grade water (Corning, 46-000-CV) and rested at room temperature for 20 min. Extracted RNA was kept at -80°C until used.

RNA samples (0.1 - 1.25 µg per reaction) were DNase treated and reverse transcribed using the QuantiTect Reverse Transcription Kit (Qiagen, 205314), as per the manufacturer’s instructions. The generated cDNA was diluted with 90-130 μl of molecular biology grade water and then used as a template to perform qRT–PCR.

**Quantitative real-time PCR (qPCR) full primer list**

| Gene | Forward Sequence (5′-3′) | Reverse Sequence (3′-5′) |
| --- | --- | --- |
| *β-tub* | ATCATCACACACGGACAGGA | GAGCTGGATGATGGGGAGTA |
| *Agpat3* | actttccgtcctgggacttt | Taattgagttcgacgacaatcg |
| *Agpat4* | cacggttctttccattttgg | Cgtatattccaaaactgttgcag |
| *mino* | Agctgcatctatgccgaaag | Tgagcgactcacgagacatc |
| *Lpin* | gatctgtttcccgacaagga | Actggaatgtttgggtcagc |
| *Lpin* | AACCGCATCAATGACGTGTG | ACTGGAATGTTTGGGTCAGC |
| *Agpat1* | ACCAAAGCTCGCTGGATGT | CGCACACGATCGATGAAG |
| *Dgat2* | Tgtccaagttgttggtgctc | Ggcactcttcgaattctcca |
| *mdy* | cagtgccgtcttccatgaat | Ccattatgcacagaggctga |
| *seipin* | CCCGTTCACATGCAGTTCAA | GCCAACCATCAGGAGTTGC |
| *Hsl* | aaatttcacAATGttatccaacg | Ttgtcggggtctcagttctc |
| *Hsl* | GCAGTCCTACGAGATTCACG | GGTCCATGTTAAGTGTAAGTATTTTGG |
| *Lsd-1* | CCGCATCATCACAATCTCAC | TGGGTGGCTGAATAATGGTT |
| *Lsd-2* | AGTCTGGCTGTCAACGGAGT | ATTGGATAGCCGTCCAACTG |
| *PAPLA1* | agcagccatgtgtgctactg | Caacagcgagtggctaacac |
| *Gpat4* | ctacccggtggccattaagt | Ttcgcgatgacactcttcac |
| *Agpat2* | agcaccatgatggaaaaagc | Gctcccaaaaatggcttgta |
| *CG15450* | tggatgtcctggaactcctc | acaaacctctgggtcacgtc |
| *wun* | TCTATCTGGCGCTCTACCTG | GATCCTGCCAGCACATCG |
| *wun2* | CCTCTTACTGTGATGCTTGTGG | TGTTCGTGGATAGCTGCTCT |
| *CG12746* | CATTACCTTCTTCAAGTTGGAGACG | TCTAAAGTCCCGCCTGTCTC |
| *mod(mdg4)* | TTTATTAGCACCGCGGAATC | GTCCACGGTCTCGATCTTGT |
| *dob* | GTGGTTCAATTTGCCGGAGT | TCCTCGGACCTGTGGAG |
| *CG5966* | TCTTTCGAGAGCTTTAAGGACA | AGGGCTTGCTATCTCCAGTC |
| *ACC* | CAAAGTACCGAGGATATACATCTCC | GATAGCCCTCACCGAGTTCA |
| *pummelig* | CGCAGTACATACACCAGTGC | CGCTGCGACTTGATCTTCTC |
| *CG11425* | ACTATGGCCGGTGTACAACA | CGAAAGATTTGGCCGACACT |
| *CG11426* | TTTCCCAGCGGTCACTCTAG | ATGGTCCATTACACGGCTGA |
| *CG11437* | CCTTACGATCGCAGTGGTTG | AAGAAGTAGGGCCTCAGTCG |
| *CG11438* | GGAGCTCTACCAGTCCTTCC | CAGCCGAACATGACGAAGAG |
| *laza* | CCCTCCTTGAACTACAGCCA | GTCGTCCTCATTCTCTGCGT |
| *CG1941* | GGAATTTTGGGGACTGGCATTT | CGTTGGAGGTGAACCCATCA |
| *CG1946* | TGTCCAAGTTGTTGGTTCCCC | TTCATCGAACATCCTCGCCA |
| *bmm* | AATGGCGTCGAATCAGACTT | AACACAGATGGGGATTTGGA |
| *β-cop* | CTCTCCGAAAATGGACTTGG | GACACCGAGTTCCGTCAAAT |

**Metabolic rate measurements**

We pushed CO_2_-free, dry air at a flow rate of 75 ml/min to purge the chambers. To prevent fly desiccation, the air was rehydrated before passing into the respiratory chambers. We used an RM8 Flow Multiplexer to switch the airstream and sequentially purge the 7 respiratory chambers for 7 min each (Sable Systems International), returning to a baseline chamber between readings for 2 min. Each individual fly was allowed to respire for 65 min while the multiplexer cycled through the other chambers. This cycle was repeated 22 times over the course of 24 hours. The water vapor was removed from the airstream post respiratory chambers using magnesium perchlorate, and the CO_2_ in the airstream was measured using a Licor 7000 infrared CO_2_ detector (Licor). This air was then scrubbed of CO_2_ using soda lime and rescrubbed of water vapor using magnesium perchlorate. O_2_ was then measured using an Oxzilla O_2_ detector (Sable Systems). After the 22^nd^ measurement, each fly was weighed.

CO_2_ data were baselined to 0 and O_2_ data were baselined and corrected using a Catmull-Rom Spline. O_2_ was inversed so that it was on the positive scale and shifted by 33 seconds to account for the lag time between CO_2_ and O_2_ readings. We then extracted the area under the curve from 15 to 95 seconds for both CO_2_ and O_2_ to provide estimates of VCO_2_ and VO_2_ for each fly at each time point. Prior to data analysis, CO_2_ and O_2_ across time were plotted to remove outlier individuals, which were primarily associated with leaky respirometry chambers indicated by highly erratic CO_2_ readings and a steady increase in O_2_ readings across time. For each individual, we calculated the average VCO_2_ and VO_2_ across multiple measures in 4 hr windows and estimated the RQ as VCO_2_/VO_2_. We also calculated an average imputed mass for each individual in each window of time, assuming a linear decrease from initial to final mass.
